# Regional brain aging signatures predict mortality through neurovascular immune mechanisms

**DOI:** 10.1016/j.xcrm.2026.103096

**Published:** 2026-10-07

**Authors:** Pei-Lin Lee, Yi-Long Huang, Chih-Kuang Liang, Wei-Ju Lee, Li-Ning Peng, Fei-Yuan Hsiao, Ching-Po Lin, Liang-Kung Chen

**Affiliations:** 1 Center for Healthy Longevity and Aging Sciences, National Yang Ming Chiao Tung University, Taipei 112304, Taiwan; 2 Center for Geriatrics and Gerontology, Kaohsiung Veterans General Hospital, Kaohsiung 813414, Taiwan; 3 Division of Neurology, Department of Internal Medicine, Kaohsiung Veterans General Hospital, Kaohsiung 813414, Taiwan; 4 Department of Geriatric Medicine, School of Medicine, National Yang Ming Chiao Tung University, Taipei 112304, Taiwan; 5 Department of Family Medicine, Taipei Veterans General Hospital Yuanshan Branch, Yilan 26444, Taiwan; 6 Center for Geriatrics and Gerontology, Taipei Veterans General Hospital, Taipei 11217, Taiwan; 7 Graduate Institute of Clinical Pharmacy, College of Medicine, National Taiwan University, Taipei 10051, Taiwan; 8 School of Pharmacy, College of Medicine, National Taiwan University, Taipei 10051, Taiwan; 9 Department of Pharmacy, National Taiwan University Hospital, Taipei 100225, Taiwan; 10 Institute of Neuroscience, National Yang Ming Chiao Tung University, Taipei 112304, Taiwan; 11 Brain Research Center, National Yang Ming Chiao Tung University, Taipei 112304, Taiwan; 12 Department of Education and Research, Taipei City Hospital, Taipei 103212, Taiwan; 13 Taipei Municipal Gan-Dau Hospital (managed by Taipei Veterans General Hospital), Taipei 112020, Taiwan

**Keywords:** brain aging, mortality prediction, magnetic resonance imaging, proteomics, longitudinal cohort study, inflammaging

## Abstract

Brain aging provides an integrative marker of systemic health, but whether regional patterns outperform whole-brain approaches remains unknown. Here, we show that regional brain age signatures achieve superior mortality prediction through identifiable neurovascular-immune mechanisms in 1,240 community-dwelling adults followed for 10 years (120 deaths). While global brain age independently predicts mortality (hazard ratio [HR], 1.044 per year), a data-driven three-region model combining the left precentral gyrus, left parahippocampal gyrus, and right cerebellum achieves superior discrimination (C-index, 0.81 vs. 0.79, *p* = 0.032). Integration with plasma proteomics (513 proteins) reveals complement activation and adaptive immune dysregulation as convergent mechanisms. Region-specific vulnerabilities emerge: microvascular dysfunction in the motor cortex, proteostasis failure in the medial temporal structures, and iron dysregulation in the cerebellum. Longitudinal imaging (*n* = 444) identifies accelerated brain aging phenotypes predicting cognitive decline (HR, 1.558; *p* = 0.042). These findings establish regional neuroimaging-proteomic integration as a precision strategy linking network-specific vulnerabilities to systemic immune processes.

## Highlights

• Global brain age gap independently predicts 10-year mortality risk
• A three-region brain age model outperforms global measures for mortality prediction
• Multi-scale integration links regional brain aging to immune-inflammatory pathways
• Accelerated brain aging predicts cognitive decline in longitudinal follow-up

## Graphical abstract

**Figure:**
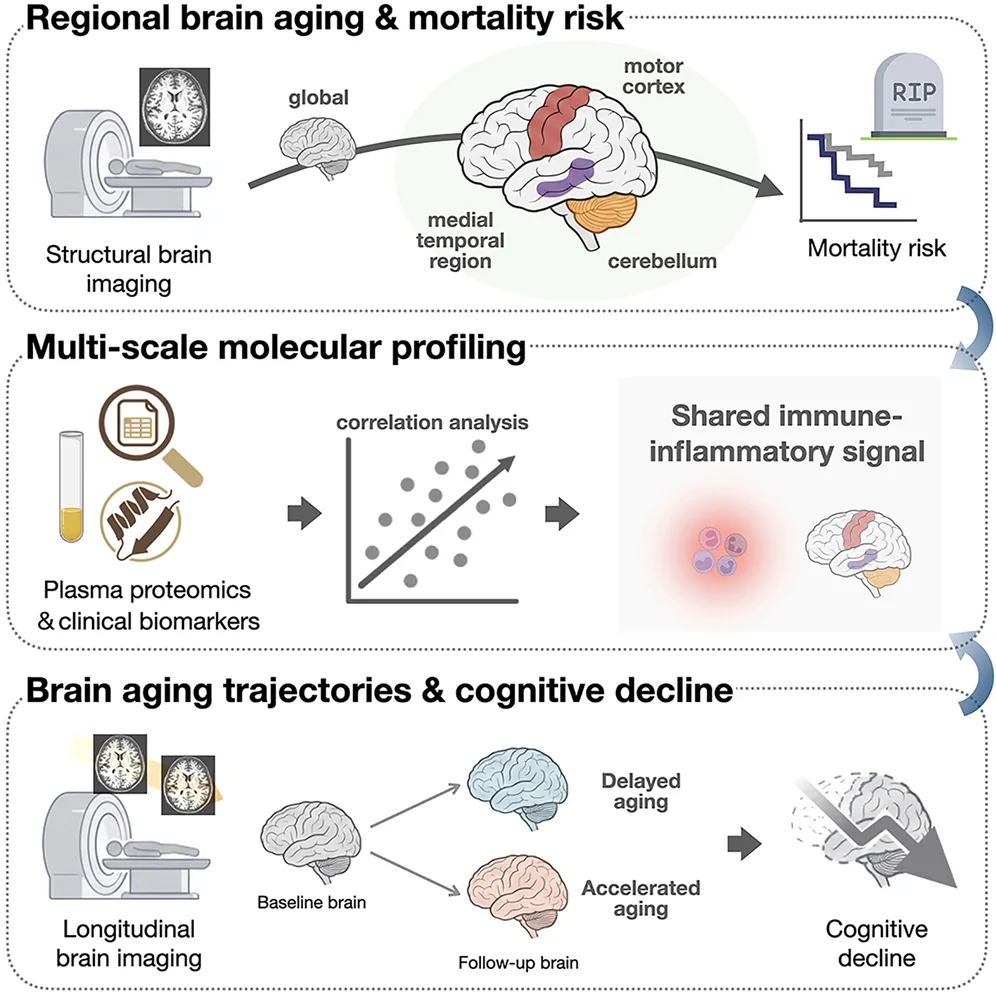

## Teaser

Lee et al. integrate structural brain MRI with plasma proteomics to identify a three-region brain age model that predicts mortality more accurately than global measures and reveals shared immune and vascular mechanisms. These findings support regional brain aging as a scalable biomarker for precision geromedicine.

## Introduction

Individuals of identical chronological age exhibit marked heterogeneity in mortality risk, with differences in accumulated health deficits accounting for much of this variation.^1^ Biological aging processes, rather than chronological time, thus govern individual vulnerability to age-related conditions, including cardiovascular disease, dementia, and frailty.^2^^,^^3^ Among various organ systems, the brain represents a particularly informative target for biological age assessment. As a metabolically demanding tissue, the brain exhibits exquisite vulnerability to systemic perturbations—including vascular dysfunction, chronic inflammation, and metabolic dysregulation—that accumulate during biological aging.^4^ These multi-system insults manifest as progressive structural changes, including tissue atrophy, synaptic loss, and network reorganization, detectable via neuroimaging years before clinical symptom onset.^4^ Despite these insights, clinical risk stratification continues to rely predominantly on chronological age, which limits precision in identifying high-risk individuals and targeting preventive interventions with precision, particularly for brain-related age-associated conditions. Recent advances in structural magnetic resonance imaging (MRI), combined with machine learning, have enabled the quantification of brain biological age from neuroimaging features, providing a data-driven measure of brain health.^5^^,^^6^ The brain age gap (BAG)—the difference between predicted and chronological brain age—quantifies deviation from normative aging, providing an integrative biomarker of biological aging independent of chronological time.

Accumulating evidence demonstrated that BAG predicted mortality risk across diverse populations, with positive values indicating elevated risk.^7^^,^^8^^,^^9^ Beyond mortality, elevated BAG predicted both progression to Alzheimer’s disease dementia and accelerated cognitive decline.^10^^,^^11^ Importantly, brain age not merely reflects localized brain pathology but serves as an integrative measure of systemic biological aging.^12^ Evidence for this systemic integration included associations with physical frailty indicators—impaired gait speed, reduced grip strength, and compromised pulmonary function^9^—and cardiometabolic disorders such as hypertension, diabetes mellitus, and cardiovascular disease.^7^ Brain age demonstrated responsiveness to modifiable lifestyle factors, with smoking cessation, regular physical activity, and moderate alcohol consumption each associated with younger brain-predicted age.^8^^,^^12^ Implementation of brain age estimation in large-scale population cohorts has established its utility as a biomarker for clinical risk stratification and as a potential therapeutic target for interventions promoting healthy brain aging.^6^

Despite these advances, three critical gaps limit mechanistic understanding and precision medicine applications. First, brain age research has predominantly utilized whole-brain summary measures, potentially obscuring regional variations that may improve mortality risk prediction. Brain aging proceeds heterogeneously across regions, with distinct neuroanatomical substrates exhibiting differential vulnerability to age-related pathology.^11^^,^^13^ Whole-brain approaches may thus overlook regional patterns containing the most robust predictive information for mortality outcomes. Regional brain age investigations have remained limited in scope and have primarily examined non-mortality outcomes; no comprehensive analysis has yet characterized how regional brain aging patterns relate to mortality risk across the full brain. Second, the mechanisms linking accelerated brain aging to mortality remain incompletely understood. The systemic processes connecting regional brain atrophy to death risk, and the pathways mediating brain-periphery communication during aging, require elucidation for rational intervention design. Blood constitutes a critical mediator, with circulating protein profiles potentially reflecting brain-periphery crosstalk.^14^^,^^15^ However, most investigations have employed targeted biomarker panels rather than unbiased proteomic profiling, with limited integration of neuroimaging-derived brain age measures and multi-domain assessments spanning inflammatory, metabolic, cardiovascular, and proteomic systems. Third, cross-sectional BAGs pose interpretational challenges, potentially reflecting historical insults or developmental variations rather than ongoing accelerated aging.^16^ Longitudinal measurements are essential to establish whether elevated BAGs represent dynamic aging trajectories conferring mortality risk. Yet, longitudinal brain age studies have remained scarce, typically involving brief follow-up intervals and lacking comprehensive phenotyping of individuals exhibiting accelerated aging trajectories.

To address these gaps, we performed a comprehensive multi-scale analysis in the I-Lan Longitudinal Aging Study (ILAS), a prospective community-based cohort in Taiwan. To derive unbiased individual brain age estimates, we developed prediction models using a large external training dataset, generating both global and regional brain age estimates across 442 brain regions encompassing cortical, subcortical, and cerebellar structures. We evaluated whether regional BAGs provided superior mortality prediction compared to whole-brain approaches and identified the optimal regional combination for mortality risk stratification. We further investigated the molecular mechanisms linking brain aging to mortality by integrating neuroimaging-derived brain age measures with comprehensive biological profiling, including circulating biomarkers spanning metabolic, inflammatory, and cardiovascular domains; physical performance assessments; cognitive performance; and untargeted plasma proteomics. In a longitudinal subset with serial neuroimaging, we characterized distinct brain aging phenotypes and examined their associations with cognitive decline trajectories and underlying proteomic signatures. We hypothesized that regional brain age patterns would exhibit heterogeneous associations with mortality risk, that systemic inflammation would emerge as a unifying molecular mechanism across brain regions, and that longitudinally defined accelerated brain aging would predict subsequent cognitive decline.

## Results

### Global brain age gap independently predicts 10-year mortality

The analysis included 1,240 participants with complete baseline data and mortality follow-up (age 63.1 ± 8.8 years, 52.9% female, 120 deaths over a median 8.75-year follow-up, Table 1). The detailed baseline characteristics stratified by mortality status are presented in Table S1. Global BAG was significantly elevated in participants who died during follow-up compared to survivors (5.11 ± 8.89 vs. 0.63 ± 9.28 years, mean difference +4.48 years, *p* < 0.001 after adjusting for age, sex, and total intracranial volume [TIV]; Figure 1A). In sex-stratified analyses, the BAG elevation was statistically significant in males (mean difference +5.17 years, *p* < 0.001) and showed a similar direction but did not reach significance in females (+3.90 years, *p* = 0.094), likely reflecting the smaller number of deaths in females.

**Table 1:** Baseline characteristics of study samples

	Cross-sectional samples (*n* = 1,240)			Longitudinal samples (*n* = 444)		
	Whole sample	Male	Female	Whole sample	Male	Female
**Demographic**						

*n*	1,240	584	656	444	206	238
Age, years	63.1 ± 8.8	64.2 ± 9.4	62.1 ± 8.1	59.2 ± 6.3	59.8 ± 6.6	58.6 ± 4.6
Education, years	6.8 ± 5.0	7.8 ± 5.1	6.0 ± 4.8	9.1 ± 4.5	9.9 ± 4.3	8.4 ± 4.6

**Lifestyle and risk factors**						

Smoking, *n* (%)	210 (16.9%)	194 (33.2%)	16 (2.4%)	53 (11.9%)	51 (24.8%)	2 (0.8%)
Alcohol drinking, *n* (%)	442 (35.6%)	312 (53.4%)	130 (19.8%)	170 (38.3%)	124 (60.2%)	46 (19.3%)
Hypertension, *n* (%)	478 (38.5%)	228 (39.0%)	250 (38.1%)	134 (30.2%)	64 (31.1%)	70 (29.4%)
Diabetes mellitus, *n* (%)	188 (15.2%)	92 (15.8%)	96 (14.6%)	46 (10.4%)	25 (12.1%)	21 (8.8%)

**Physical examination**						

Handgrip, kg	29.0 ± 9.5	36.2 ± 8.0	22.6 ± 5.2	30.7 ± 9.9	39.3 ± 6.8	23.3 ± 5.0
Gait speed, m/s	1.6 ± 0.5	1.7 ± 0.5	1.5 ± 0.4	1.7 ± 0.5	1.8 ± 0.5	1.6 ± 0.4

**Cognitive performance, scores**						

Mini-Mental State Examination	26.2 ± 3.6	26.7 ± 3.2	25.7 ± 3.9	27.5 ± 2.5	28.0 ± 1.9	27.1 ± 3.0
10-min CVVLT	6.6 ± 2.1	6.4 ± 2.1	6.8 ± 2.0	7.1 ± 1.7	7.0 ± 1.8	7.3 ± 1.6
Clock drawing test	7.7 ± 2.5	8.0 ± 2.3	7.4 ± 2.7	8.6 ± 1.9	8.8 ± 1.6	8.4 ± 2.1
Taylor Complex Figure test	30.6 ± 7.1	31.4 ± 6.0	29.8 ± 7.8	32.8 ± 4.8	33.4 ± 3.7	32.4 ± 5.6
Boston Naming test	12.4 ± 2.5	13.0 ± 2.3	11.9 ± 2.6	13.3 ± 2.0	13.8 ± 1.6	12.8 ± 2.2
Backward Digit test	3.6 ± 2.0	3.9 ± 1.8	3.4 ± 2.0	4.3 ± 1.7	4.6 ± 1.5	4.1 ± 1.8

**Neuroimaging measurement, mL**						

Total intracranial volume	1,336 ± 118	1,414 ± 97	1,266 ± 88	1,339 ± 120	1,419 ± 99	1,270 ± 90
Gray-matter volume	629 ± 61	654 ± 58	606 ± 53	645 ± 54	671 ± 50	622 ± 48
White-matter volume	416 ± 50	439 ± 48	395 ± 42	425 ± 48	450 ± 46	404 ± 39
Cerebrospinal fluid volume	292 ± 73	321 ± 70	265 ± 64	269 ± 64	298 ± 63	245 ± 55
Death, *n* (%)	120 (9.7%)	78 (13.4%)	42 (6.4%)	–	–	–
Follow-up periods of mortality tracing, years	8.66 ± 1.30	8.64 ± 1.44	8.67 ± 1.16	–	–	–
Follow-up intervals of MRI scans, years	–	–	–	7.8 ± 1.3	7.9 ± 1.2	7.8 ± 1.3

CVVLT, Chinese version Verbal Learning Test.

**Figure 1:**
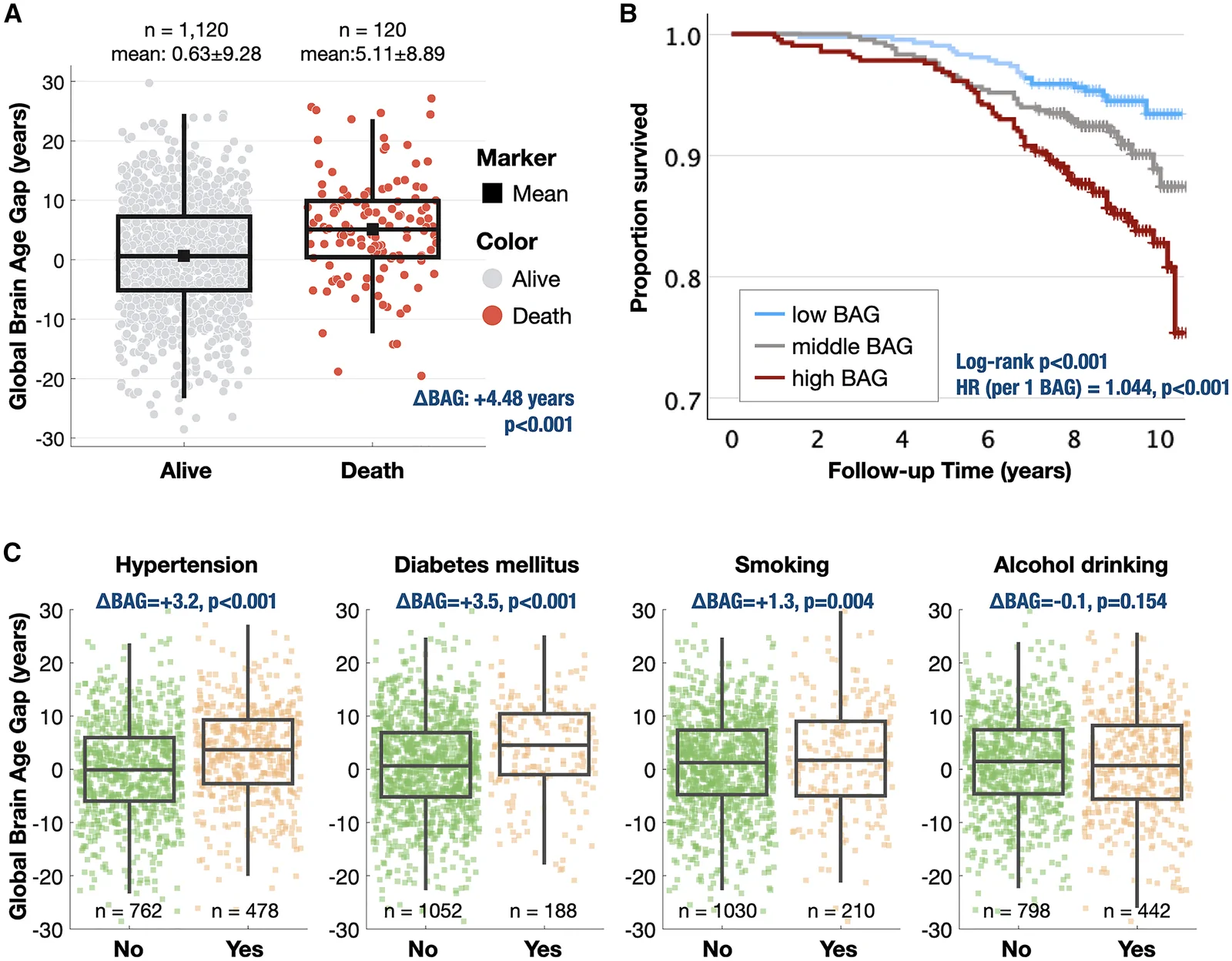
Global brain age gap associates with all-cause mortality risk (A) Global BAG distribution by survival status (alive *n* = 1,120, died *n* = 120). Boxplots show median and interquartile range; dots represent individual participants. Deceased individuals showed significantly elevated BAG (*p* < 0.001, ANCOVA adjusted for age, sex, and TIV). (B) Kaplan-Meier survival curves stratified by global BAG tertiles: low BAG (blue line), middle BAG (gray line), and high BAG (red line). Censoring events indicated by cross marks. The log-rank *p* value compares survival across tertiles; HR indicates the hazard ratio per 1-year increase in global BAG from Cox regression model. (C) Global BAG across cardiovascular risk factor groups. Boxplots compare BAG between individuals with (orange) and without (green) hypertension, diabetes, smoking, and alcohol use. The group comparison analyses were adjusted for age, sex, and TIV. BAG, brain age gap; TIV, total intracranial volume; ΔBAG, difference in mean BAG between groups.

Cox proportional hazards regression analysis established the global BAG as an independent predictor of mortality risk. In the minimally adjusted model controlling for age, sex, and TIV, each additional year of BAG was associated with a 4.4% increased hazard of death (hazard ratio [HR] = 1.044, 95% confidence interval [CI]: 1.022–1.067, *p* < 0.001; Table 2). The association remained statistically significant following comprehensive adjustment for potential confounders, including education, smoking status, alcohol consumption, hypertension, and diabetes mellitus (HR = 1.037, 95% CI: 1.015–1.059, *p* = 0.001; Table 2). To formally test whether sex modified this association, we further included a BAG × sex interaction term in the fully adjusted Cox regression model. The interaction term was not statistically significant (HR = 0.988, 95% CI: 0.944–1.034, *p* = 0.610), indicating that sex did not significantly modify the BAG-mortality association after adjustment for demographic, lifestyle, and cardiovascular risk factors. Kaplan-Meier survival curves stratified by global BAG tertiles provided visual confirmation of these dose-response relationships (Figure 1B), demonstrating progressive separation across strata (log rank test *p* < 0.001). Global BAG showed significant associations with established cardiovascular risk factors (Figure 1C). Participants with hypertension had higher BAG compared to those without (mean difference +3.2 years, *p* < 0.001). Similarly, participants with diabetes mellitus showed elevated BAG (+3.5 years, *p* < 0.001), as did current or former smokers compared to never-smokers (+1.3 years, *p* = 0.004). No significant association was observed between BAG and alcohol consumption (*p* = 0.154).

**Table 2:** Cox proportional hazards regression models for all-cause mortality risk

Variables	Model 1 Global BAG (minimal adjustment)			Model 2 Global BAG (full adjustment)			Model 3 Optimal regional BAGs (minimal adjustment)			Model 4 Optimal regional BAGs (full adjustment)		
–	HR	95% CI	*p* value	HR	95% CI	*p* value	HR	95% CI	*p* value	HR	95% CI	*p* value
Age, per year	1.096	1.075–1.118	<0.001	1.084	1.059–1.110	<0.001	1.079	1.057–1.102	<0.001	1.071	1.045–1.097	<0.001
Female (vs. male)	0.733	0.454–1.183	0.204	0.806	0.470–1.384	0.435	0.722	0.447–1.167	0.183	0.743	0.431–1.281	0.285
TIV, per 100 mL	1.151	0.946–1.400	0.161	1.156	0.950–1.407	0.148	1.293	1.064–1.570	0.010	1.251	1.032–1.517	0.023
Education, per year	–	–	–	0.983	0.937–1.031	0.486	–	–	–	0.987	0.940–1.035	0.582
Smoking (vs. no)	–	–	–	2.284	1.477–3.533	<0.001	–	–	–	2.169	1.394–3.374	<0.001
Alcohol drinking (vs. no)	–	–	–	0.655	0.429–0.998	0.049	–	–	–	0.622	0.405–0.955	0.03
Hypertension (vs. no)	–	–	–	1.757	1.180–2.617	0.006	–	–	–	1.617	1.082–2.416	0.019
Diabetes mellitus (vs. no)	–	–	–	1.286	0.835–1.981	0.254	–	–	–	1.116	0.721–1.726	0.623
Brain age metrics												
Global BAG, per year	1.044	1.022–1.067	<0.001	1.037	1.015–1.059	0.001	–	–	–	–	–	–
Regional BAG in left precentral gyrus, per year	–	–	–	–	–	–	1.016	1.005–1.026	0.003	1.014	1.004–1.025	0.007
Regional BAG in left parahippocampal, per year	–	–	–	–	–	–	1.019	1.008–1.031	<0.001	1.016	1.004–1.028	0.007
Regional BAG in right cerebellum I–IV, per year	–	–	–	–	–	–	1.022	1.007–1.037	0.004	1.021	1.006–1.036	0.007
Model performance												
Concordance index (C-index)	0.772	–	–	0.791	–	–	0.797	–	–	0.811	–	–
AIC	1,532.0	–	–	1,516.4	–	–	1,507.8	–	–	1,498.1	–	–

AIC, Akaike information criterion; BAG, brain age gap; TIV, total intracranial volume; HR, hazard ratio; CI, confidence interval.

### Three-region model achieves superior mortality discrimination

Univariate Cox regression analysis of regional BAGs across 442 brain regions identified 44 regions significantly associated with mortality after Bonferroni correction (*p* < 1.13 × 10^−4^; Figure 2A; Table S2). For anatomical interpretation, cortical regions were mapped to Harvard-Oxford anatomical labels. Network distribution showed that sensorimotor network regions comprised 34% of significant areas (15 regions), followed by default mode network (14%, 6 regions), subcortical structures (11%, 5 regions), and visual networks (11%, 5 regions; Figure 2B). Subcortical structures demonstrated the strongest associations, with the right and left thalamus showing the highest hazard ratios (HR = 1.050, 95% CI: 1.025–1.075, *p* < 0.001; HR = 1.049, 95% CI: 1.024–1.074, *p* < 0.001, respectively; Figure 2C). All 44 regions remained significant (*p* < 0.05) after full adjustment for demographics, lifestyle factors, and cardiovascular risk factors (Table S3). Forward stepwise selection identified an optimal three-region model (Figure 3A; Table 2). The selected regions represent distinct functional systems: left precentral gyrus (sensorimotor network), left parahippocampal gyrus anterior division (limbic network), and right cerebellum I–IV (cerebellar system). Inter-correlations among the global and the three regional BAGs were modest (Table S4), supporting their capture of partially independent brain aging information. Multivariate Cox regression demonstrated independent contributions from each region: left precentral gyrus (HR = 1.016, 95% CI: 1.005–1.026, *p* = 0.003), left parahippocampal gyrus (HR = 1.019, 95% CI: 1.008–1.031, *p* < 0.001), and right cerebellum I–IV (HR = 1.022, 95% CI: 1.007–1.037, *p* = 0.004). Without any covariate adjustment, the three regional BAGs jointly yielded a C-index of 0.73 (95% CI: 0.68–0.77), while the global BAG alone yielded 0.62 (95% CI: 0.57–0.67), both exceeding chance-level discrimination. Model performance comparison showed progressive improvement (Figure 3B). C-index values ranged from 0.75 (baseline demographic model) to 0.81 (three-region model with expanded covariates). The three-region model showed superior discrimination over the global BAG (C-index: 0.80 vs. 0.77, bootstrap *p* = 0.034 with basic demographics; 0.81 vs. 0.79, bootstrap *p* = 0.032 with expanded covariates).

**Figure 2:**
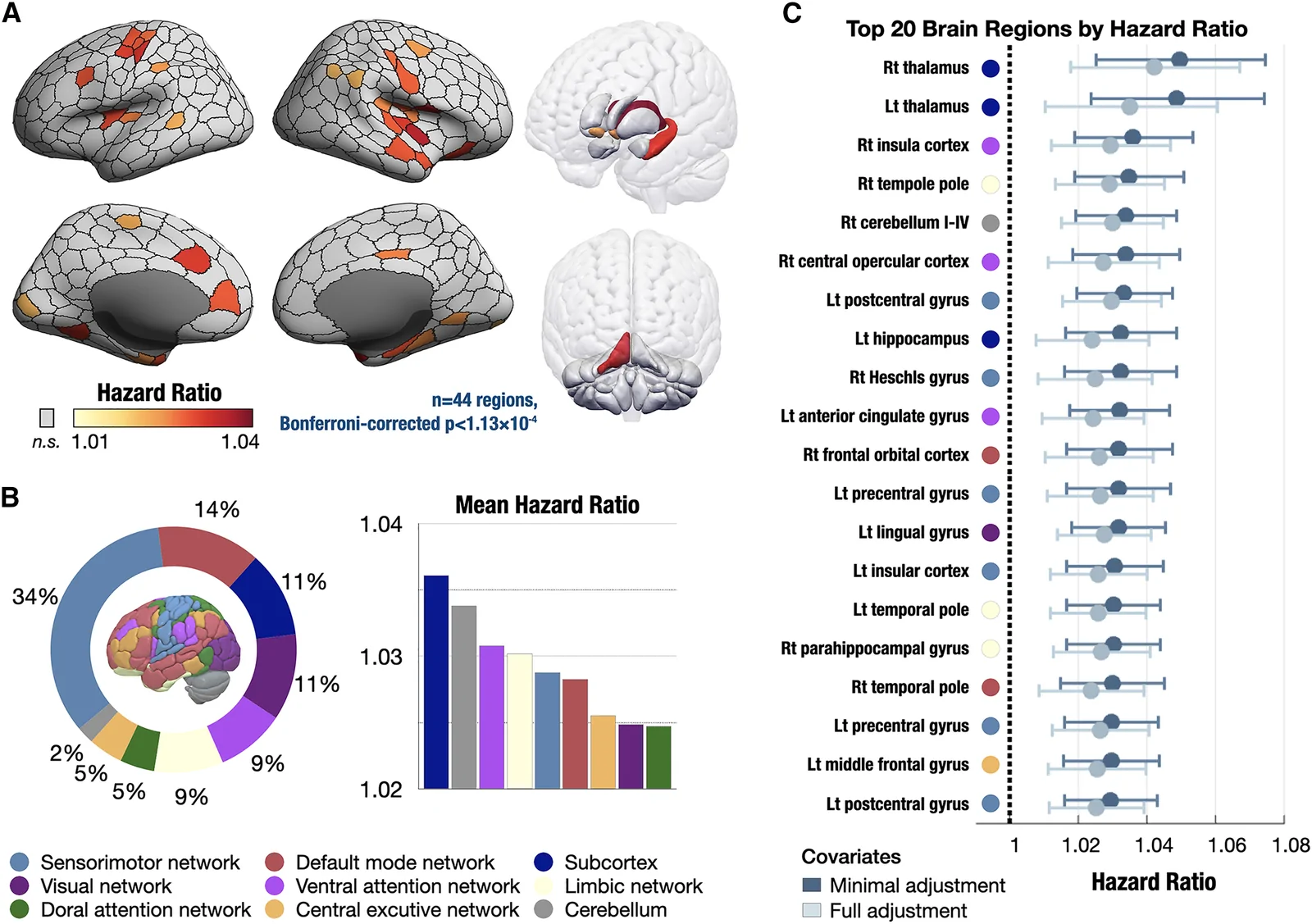
Regional brain age patterns reveal network-specific mortality associations (A) Spatial distribution of mortality-associated brain regions. Brain surface maps showing 44 regions that achieved Bonferroni-corrected significance (*p* < 1.13 × 10^−4^) from 442 regions tested. Color intensity indicates HR strength; non-significant regions are shown in light gray. All models adjusted for age, sex, and TIV. (B) Network-level distribution of 44 significant regions. Left: pie chart showing proportion of 44 regions by brain network, with sensorimotor network (34%) and default mode network (14%) predominating. Right: the bar plot shows the mean HR of those significant regions within each network. (C) Forest plot of the top 20 brain regions ranked by HR from each Cox regression model, displaying hazard ratios with 95% confidence intervals for minimally adjusted (light points: age, sex, and TIV) and fully adjusted (dark points: additional covariates including education, smoking, alcohol, hypertension, and diabetes) models. BAG, brain age gap; HR, hazard ratio; TIV, total intracranial volume; Lt/Rt, left/right hemisphere.

**Figure 3:**
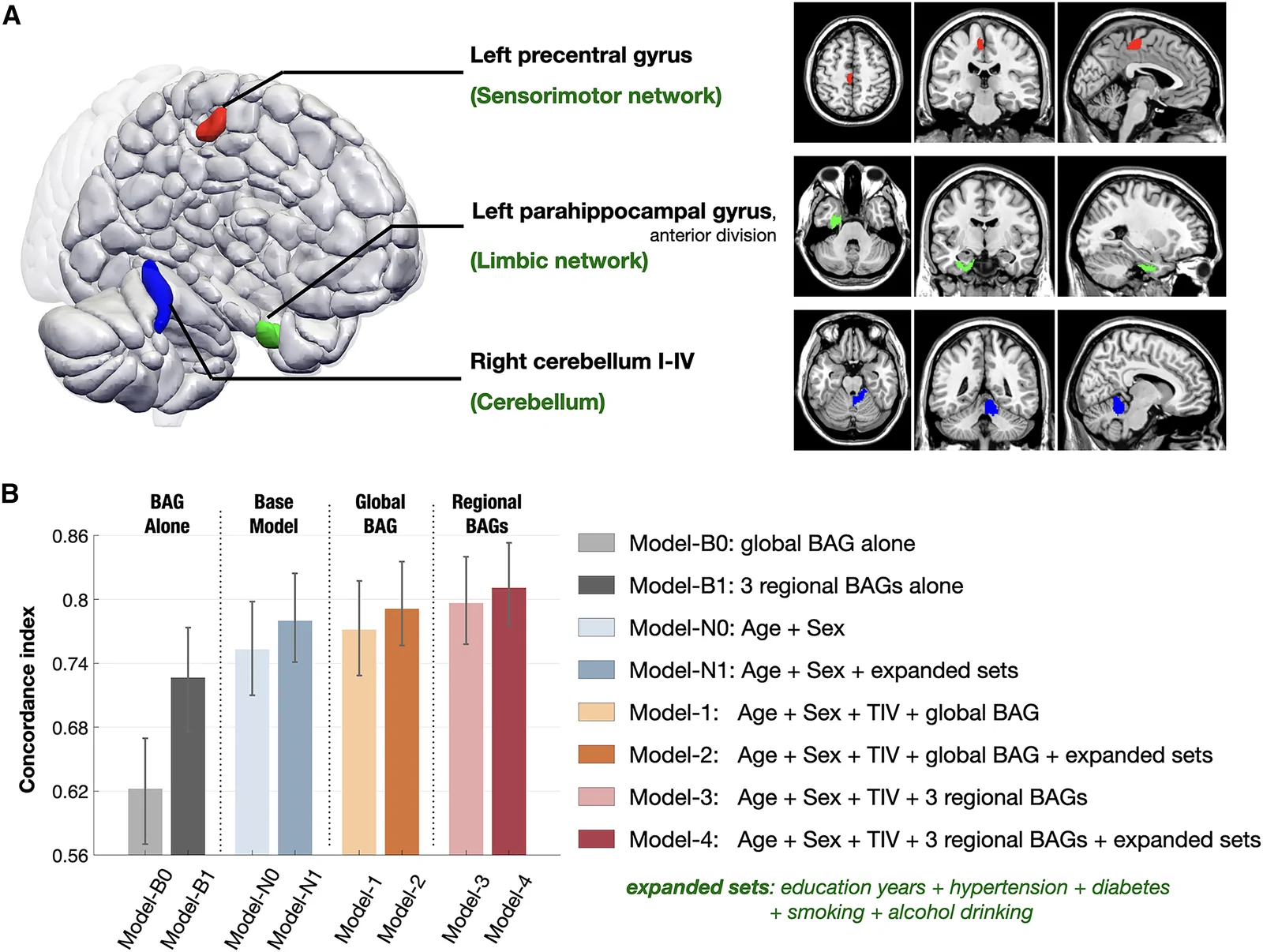
Three-region brain age model optimally predicts mortality risk (A) Anatomical localization of three optimal regions selected from 44 mortality-associated candidates via forward stepwise regression. Left: 3D brain rendering. Right: MRI slices showing each region in axial, coronal, and sagittal views. Regions: left precentral gyrus (sensorimotor, red), left parahippocampal gyrus (limbic, green), and right cerebellum I–IV (blue). (B) Model discrimination performance (concordance index). Eight models compared: (1) global BAG only (C = 0.62), (2) three regional BAGs only (C = 0.73), (3) age + sex (C = 0.75), (4) model 3 + risk factors (C = 0.78), (5) model 3 + global BAG (C = 0.77), (6) model 4 + global BAG (C = 0.79), (7) model 3 + 3 regional BAGs (C = 0.80), (8) model 4 + 3 regional BAGs (C = 0.81). Error bars: 95% CI from bootstrap. BAG, brain age gap; C, concordance index.

### Multi-scale integration reveals convergent inflammatory mechanisms

To investigate the biological mechanisms underlying regional brain aging patterns, we examined the associations between the global BAG and three regional BAGs with 46 plasma biomarkers, 3 physical function assessments, and 6 cognitive test scores (Figure 4A; Table S5). A convergent inflammatory signature emerged as the primary shared mechanism across all brain age measures. Homocysteine and neutrophil count showed consistent positive correlations with the global BAG and all three regional BAGs. Hematologic parameters, including white blood cell count and platelet count, demonstrated similar cross-regional associations. Beyond these shared patterns, region-specific biomarker associations were observed across the hepatic, renal, and endocrine domains. Cognitive performance measures demonstrated negative correlations with BAGs, with the language, visual-spatial, and executive function domains showing the strongest and most consistent associations across regions. Physical function measures (grip strength and gait speed) showed similar negative correlations across all brain regions.

**Figure 4:**
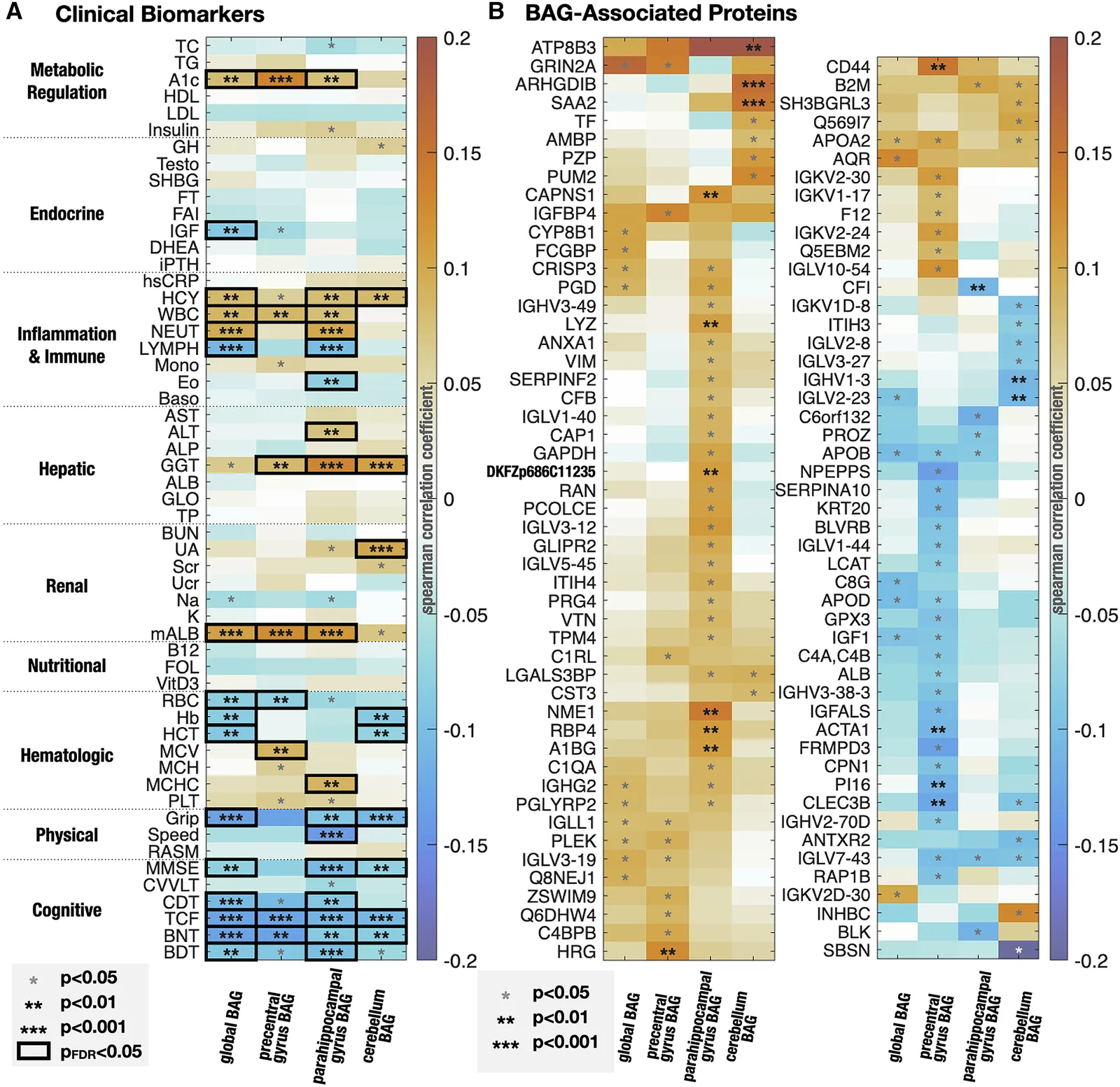
Multi-scale molecular validation of global and regional brain aging Heatmaps showing partial Spearman correlations between molecular biomarkers (rows) and brain age gaps (columns): global, left precentral gyrus, left parahippocampal gyrus, and right cerebellum. ∗*p* < 0.05, ∗∗*p* < 0.01, ∗∗∗*p* < 0.001. All correlations adjusted for age, sex, and TIV. (A) Clinical biomarkers (*n* = 1,240). Fifty-five biomarkers across nine domains: metabolic, endocrine, inflammation and immune, hepatic, renal, nutritional, hematologic, physical, and cognitive. Black boxes indicate FDR-corrected associations (*q* < 0.05). (B) Plasma proteomics (*n* = 606). Display shows 99 of 513 proteins measured, selected for uncorrected *p* < 0.05 with at least one brain age measure. Complete annotations and statistical results are provided in Table S5 for clinical biomarkers and Table S6 for plasma proteins. BAG, brain age gap; FDR, false discovery rate; TIV, total intracranial volume.

Proteomic analysis revealed 19–40 region-associated proteins per brain region (Figure 4B; Table S6; Figure S1A). Global BAG showed associations with proteins including APOD, APOB, and GRIN2A. Region-specific proteomic signatures included hemostatic and extracellular matrix-associated proteins for the precentral gyrus (HRG and CD44), metabolic and proteolytic markers associated with neurodegeneration for the parahippocampal gyrus (NME1 and CAPNS1), and inflammatory and membrane-integrity-related proteins for the cerebellum (SAA2 and ATP8B3). These differential protein signatures suggest distinct molecular pathways underlying regional brain aging vulnerabilities. Pathway enrichment analysis of brain age-associated proteins revealed convergent immune response activation across all regions, alongside region-specific signatures including coagulation pathways for the precentral gyrus, acute-phase response and proteolytic regulation for the parahippocampal gyrus, and notably, iron homeostasis for the cerebellum (Figure S1B). Cross-database validation using Reactome, KEGG, and WikiPathways confirmed the convergent immune/complement mechanism across all regions and provided independent support for the region-specific signatures identified through GO enrichment (Table S7).

### Accelerated brain aging predicts cognitive decline

Longitudinal analysis of 444 participants with repeat MRI over a median 8-year interval revealed heterogeneous brain aging trajectories (Figures 5A and 5B). Based on the annual rate of global BAG change adjusted for baseline BAG and scanner upgrade effects, participants were classified into three phenotypes: delayed aging ([DA], *n* = 72, 16.2%), normal aging ([NA], *n* = 306, 68.9%), and accelerated aging ([AA], *n* = 66, 14.9%). The baseline characteristics and aging trajectories are summarized in Table 3. Significant sex differences were observed, with females comprising 69.4% of the delayed aging group compared to 39.4% of the accelerated aging group (*p* = 0.002). No significant differences were observed in age, education, smoking, alcohol consumption, hypertension, diabetes, or MRI scanner upgrade across phenotypes. Baseline cognitive performance was also comparable among the three groups. Cox proportional hazards regression analysis, adjusting for age, sex, and education, indicated that accelerated aging was associated with an increased risk of cognitive decline compared to normal aging (HR = 1.558, 95% CI: 1.016–2.390, *p* = 0.042). Kaplan-Meier survival curves suggested a trend toward earlier onset of cognitive decline in the accelerated aging group, though this did not reach statistical significance in unadjusted log rank testing (*p* = 0.125; Figure 5C).

**Figure 5:**
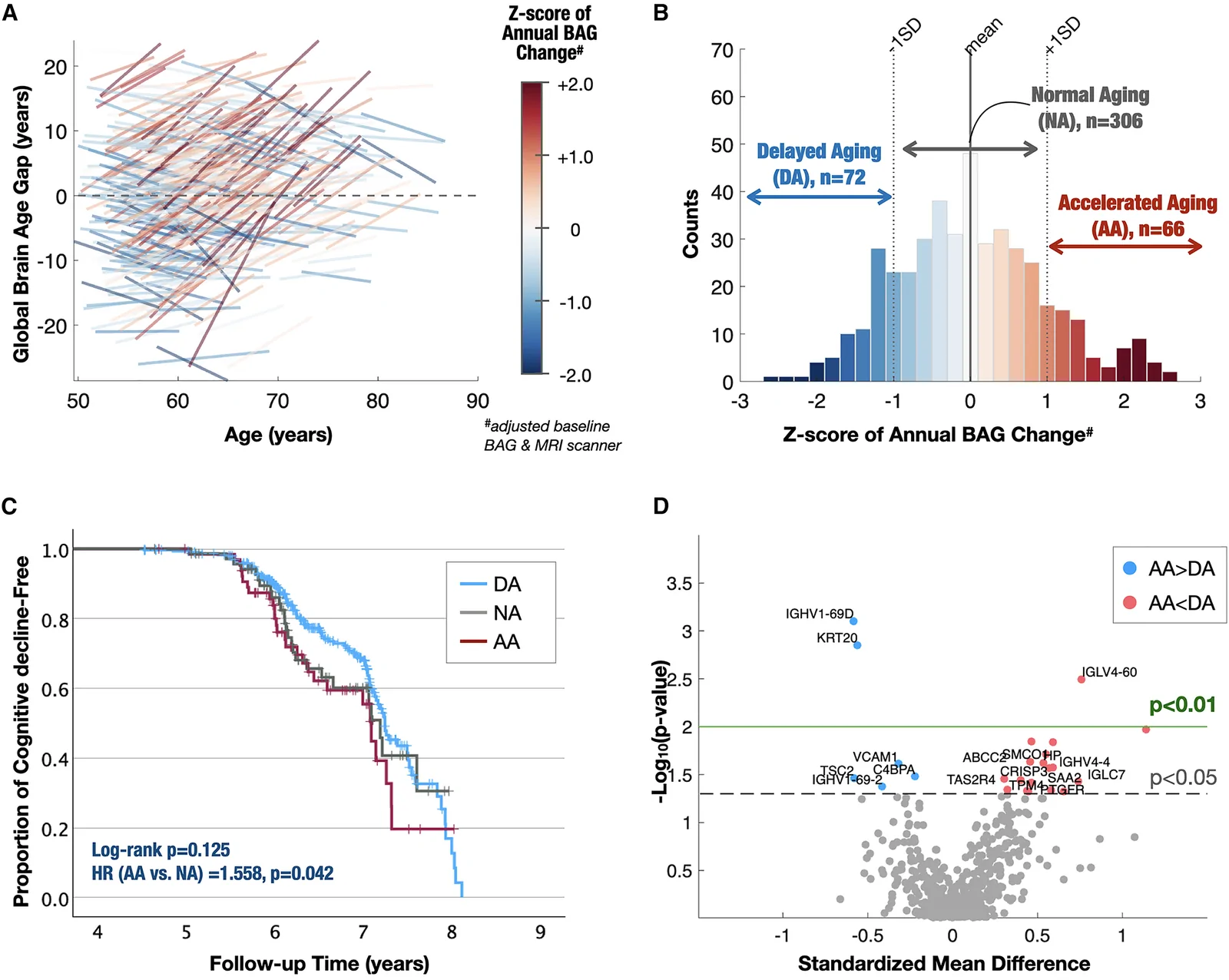
Longitudinal aging phenotypes and clinical outcomes (A) Individual brain age trajectories over 8-year follow-up (*n* = 444 participants with repeat MRI). Spaghetti plot showing brain age gap vs. chronological age, with each line representing one participant, color-coded by adjusted *Z* score of annual rate of BAG change. (B) Phenotype distribution based on trajectory slopes. Histogram showing frequency distribution of annual global BAG change rates, defining three phenotypes: DA (blue, *n* = 72), NA (gray, *n* = 306), and AA (red, *n* = 66). (C) Cognitive outcomes by brain aging phenotype. Kaplan-Meier curves showing cognitive decline-free survival (cognitive decline defined as top 10% decline in any cognitive domain). Accelerated aging showed a trend toward shorter cognitive decline-free survival in the Kaplan-Meier analysis and was associated with increased cognitive decline risk in adjusted Cox regression. Cross marks indicate censored observations. HR indicates the hazard ratio for AA versus NA from adjusted Cox regression model. (D) Proteomic signatures distinguishing phenotypes. Volcano plot comparing protein difference between AA and DA groups. Blue points indicate proteins elevated in AA, red points indicate proteins elevated in DA, and gray points indicate non-significant proteins. The dashed and solid horizontal lines mark *p* = 0.05 and *p* = 0.01 significance thresholds, respectively. BAG, brain age gap; DA/NA/AA, delayed/normal/accelerated aging; MRI, magnetic resonance imaging.

**Table 3:** Characteristics of longitudinal brain aging phenotype

Variables	Delayed aging (*n* = 72)	Normal aging (*n* = 306)	Accelerated aging (*n* = 66)	*p* value
**Demographics at baseline**				

Age, years	57.8 ± 7.0	59.5 ± 6.2	59.0 ± 5.4	0.119
Female, *n* (%)	50 (69.4%)	162 (52.9%)	26 (39.4%)	0.002
Education, years	9.2 ± 4.5	8.8 ± 4.5	9.9 ± 4.7	0.217

**Lifestyle and risk factors**				

Smoking, *n* (%)	8 (11.1%)	34 (11.1%)	11 (16.7%)	0.438
Alcohol drinking, *n* (%)	20 (27.8%)	123 (40.2%)	27 (40.9%)	0.133
Hypertension, *n* (%)	16 (22.2%)	92 (30.1%)	26 (39.4%)	0.090
Diabetes mellitus, *n* (%)	4 (5.6%)	32 (10.5%)	10 (15.2%)	0.180

**Brain age metrics**				

Baseline global BAG, years	−1.26 ± 9.41	−0.46 ± 9.30	−0.55 ± 9.59	0.542^a^
Follow-up intervals, years (MRI scans)	7.85 ± 1.38	7.90 ± 1.24	7.71 ± 1.39	0.553
Annual BAG change (ΔBAG), years	−0.42 ± 0.28	0.32 ± 0.33	1.20 ± 0.37	<0.001^b^
MRI upgrade, *n* (%)	30 (41.7%)	133 (43.5%)	28 (42.4%)	0.957

**Cognitive performance at baseline**				

MMSE score	27.5 ± 2.4	27.4 ± 2.5	27.8 ± 3.0	0.988^c^
CVVLT score	6.9 ± 1.9	7.2 ± 1.7	7.2 ± 1.6	0.103^c^
CDT score	8.8 ± 1.9	8.5 ± 1.9	8.8 ± 1.9	0.851^c^
TCF score	32.6 ± 6.2	32.9 ± 4.4	32.8 ± 4.8	0.335^c^
BNT score	13.3 ± 1.9	13.2 ± 2.1	13.4 ± 2.0	0.598^c^
BDT score	4.3 ± 1.7	4.3 ± 1.7	4.4 ± 1.5	0.404^c^

**Cognitive outcome**				

Cognitive decline, n (%)	26 (36.1%)	104 (34.0%)	28 (42.4%)	0.428
CVVLT decline, n (%)	7 (9.7%)	30 (9.8%)	7 (10.6%)	0.979
CDT decline, n (%)	8 (11.1%)	29 (9.5%)	7 (10.6%)	0.897
TCF decline, n (%)	4 (5.6%)	33 (10.8%)	7 (10.6%)	0.401
BNT decline, n (%)	6 (8.3%)	30 (9.8%)	8 (12.1%)	0.754
BDT decline, n (%)	7 (9.7%)	30 (9.8%)	7 (10.6%)	0.979
Follow-up intervals, years (cognitive assessments)	6.34 ± 0.71	6.43 ± 0.72	6.32 ± 0.66	0.381

BAG, brain age gap; CVVLT, Chinese version Verbal Learning Test; CDT; clock drawing test; TCF, Taylor Complex Figure test; BNT, Boston Naming test; BDT, Backward Digit test.

a ANCOVA test, adjusted for age, sex, and total intracranial volume.

b ANCOVA test, adjusted for age and sex.

c ANCOVA test, adjusted for age, sex, and education years.

### Proteomic signatures distinguishing extreme aging phenotypes

To investigate the molecular mechanisms underlying differential aging trajectories, we compared the plasma proteomic profiles between extreme phenotypes in 71 participants with available proteomics data (AA: *n* = 31, DA: *n* = 40). Twenty-five proteins showed significant differences (*p* < 0.05; Figure 5D; Table S8). Delayed aging was characterized by elevated immunoglobulin proteins, including immunoglobulin lambda variable 4-60 ([IGLV4_60], *p* = 0.003), immunoglobulin heavy variable 4-4 ([IGHV4_4], *p* = 0.027), and immunoglobulin lambda constant 7 ([IGLC7], *p* = 0.037), alongside serum amyloid A2 ([SAA2], *p* = 0.038) and serum deprivation-response protein ([SDPR], *p* = 0.011). Accelerated aging showed higher levels of immunoglobulin heavy variable 1-69D ([IGHV1_69D], *p* = 0.001), keratin 20 ([KRT20], *p* = 0.001), vascular cell adhesion molecule 1 ([VCAM1], *p* = 0.024), and complement component 4-binding protein alpha ([C4BPA], *p* = 0.033).

## Discussion

This study investigated whether regional brain age patterns improve mortality prediction beyond global measures and examined the molecular mechanisms and longitudinal trajectories underlying brain aging-mortality associations in 1,240 community-dwelling participants from the ILAS cohort. First, the global BAG constituted an independent predictor of 10-year mortality risk, with each additional year associated with 4.4% increased hazard of death after adjusting for demographic factors. Next, regional brain age analysis across 442 brain regions revealed that a three-region model comprising the left precentral gyrus, left parahippocampal gyrus, and right cerebellum I–IV showed modestly improved discrimination (C-index, 0.81) compared to global brain age approaches (C-index, 0.79). Then, integration of neuroimaging-derived brain age measures with blood biomarkers and discovery-based proteomics identified inflammation as a convergent molecular pathway. Furthermore, longitudinal characterization in 444 participants with repeated neuroimaging revealed heterogeneous brain aging trajectories, with accelerated aging phenotype associated with increased risk of subsequent cognitive decline. These findings demonstrate that brain aging proceeds through network-specific regional vulnerabilities linked to systemic inflammatory processes, with longitudinally defined phenotypes predicting differential clinical outcomes.

Mortality risk stratification in aging populations relies predominantly on chronological age, despite growing recognition that biological aging heterogeneity determines survival outcomes. Recent plasma proteomic studies established that organ-specific aging—particularly brain and immune system aging—predicts mortality independently of chronological age, with extremely aged brains conferring 182% increased death risk.^17^ However, these breakthrough studies employed proteomics-derived brain age rather than direct neuroimaging assessment, leaving unresolved whether regional neuroanatomical patterns enhance prognostic precision beyond global measures. We found that regional brain age signatures derived from structural MRI provided modest additional discriminative value over global brain age for mortality prediction. Beyond discrimination, region-specific biomarker and proteomic correlates diverged across brain regions rather than converging on a single global signature, extending prior proteomic studies of brain and organ aging, which have primarily characterized correlates of global brain age, to a spatially resolved level. Longitudinally defined phenotypes further showed distinct proteomic profiles associated with differential cognitive trajectories. These results add a regionally resolved, multi-scale dimension to the association between brain aging and mortality within a single cohort.

Regional brain age architecture captured mortality-relevant information that whole-brain averaging obscured, extending recent recognition of brain aging heterogeneity to survival outcomes. While Yang et al. identified five distinct atrophy patterns across 49,482 individuals, their patterns required complex deep learning decomposition without direct mortality optimization.^18^ Our forward stepwise selection distilled the 44 mortality-associated regions to three anatomically interpretable structures—left precentral gyrus (primary motor cortex), left parahippocampal gyrus (medial temporal memory system), and right cerebellum I–IV—showing modestly improved discrimination (C-index, 0.81) compared to global brain age (C-index, 0.79). This regional pattern is broadly consistent with emerging evidence that brain aging proceeded through network-specific vulnerabilities rather than uniform deterioration,^19^^,^^20^ with particular regions demonstrating selective susceptibility to the “last-in, first-out” principle linking late-developing areas to accelerated age-related degeneration. The concentration of mortality-associated regions within the sensorimotor network (34% of significant regions) reflected motor system integrity as a fundamental survival determinant, consistent with epidemiological evidence linking gait speed, grip strength, and motor decline to mortality risk.^21^^,^^22^ The precentral gyrus governs voluntary movement control, with its accelerated aging predicting frailty cascades culminating in mortality.^23^ The parahippocampal gyrus integrated memory and spatial orientation, showing particular vulnerability to age-related neurodegeneration with implications for functional independence.^24^ The cerebellum’s contribution extended beyond motor coordination to cognitive processing and autonomic regulation, with cerebellar vulnerability increasingly recognized as a driver of late-life physiological instability.^25^^,^^26^ This parsimonious three-region model offered practical advantages over multi-dimensional aging indices, providing interpretable biomarkers amenable to clinical implementation while capturing prognostically relevant heterogeneity.

Multi-scale integration of neuroimaging-derived brain age, blood biomarkers, and discovery proteomics identified systemic inflammation as a dominant pathway associated with regional neural aging and mortality, a finding converging with recent plasma proteomic organ aging studies. Liu et al. reported undulating changes in plasma proteome across brain aging, identifying BCAN and GDF15 as key biomarkers with peaks at ages 57, 70, and 78 years.^27^ Similarly, Casanova et al. identified 33 proteins associated with the global BAG in an aged and ethnically diverse cohort, with GDF15 and SEVP1 among the most robust positive associations.^7^ The most consistent BAG-associated proteins reported in these two studies were low-abundance signaling proteins, including growth factors (GDF15 and FGF21) and glial markers (GFAP and CHI3L1). None of these proteins were among those detected in our mass spectrometry-based analysis, which instead identified higher-abundance plasma proteins associated with the global BAG, including apolipoproteins (APOA2, APOB, and APOD), immunoglobulin variants, complement C8G, and IGF-1. This divergence at the individual-protein level is consistent with well-recognized differences in protein coverage across proteomic platforms, whereby the targeted platforms used in the two previous studies preferentially quantify low-abundance signaling proteins, while mass spectrometry-based untargeted approaches primarily capture higher-abundance plasma proteins. Notwithstanding these platform-related differences, convergence emerged at the pathway level, with inflammatory and immune activation pathways identified as common correlates linking the plasma proteome to brain aging across all three studies. While these prior studies characterized such pathways in relation to global brain age, our neuroimaging-anchored approach additionally examined whether the same pathways were detectable at the level of specific brain regions: gene ontology analysis revealed immune response pathways—particularly complement activation and adaptive immune processes—as the most enriched themes across all mortality-associated brain regions; blood biomarker profiling showed homocysteine, neutrophil counts, and white blood cell counts consistently associated with regional BAGs. At the individual-protein level, cross-regional consistency was limited, with immunoglobulin lambda variable 7-43 (IGLV7-43) being the only protein showing nominal association across all three regional BAGs, congruent with the immune convergent theme. In the longitudinal cohort, discovery proteomics identified distinct immunoglobulin profiles distinguishing accelerated from delayed aging. This convergence across molecular scales was consistent with the inflammaging framework positioning chronic immune dysregulation as a contributing process linking systemic and neural aging,^3^ recently reinforced by evidence that inflammaging drove neurodegenerative progression through persistent microglial activation and cytokine release.^28^^,^^29^ Critically, our approach identified region-specific molecular signatures suggestive of distinct vulnerabilities beyond global inflammatory signatures. The precentral gyrus demonstrated enrichment for coagulation and fibrinolysis pathways, reflecting motor cortex microvascular vulnerability where vascular-motor coupling integrity determines mobility and survival,^30^^,^^31^ a pathway undetectable through plasma proteomics. The parahippocampal gyrus showed proteolytic regulation and tissue repair pathway enrichment, suggesting disturbed protein homeostasis in medial temporal structures. The cerebellum exhibited iron homeostasis pathway enrichment, with iron dyshomeostasis contributing to oxidative stress and functional decline.^32^ These region-specific signatures provide candidate directions for targeted interventions impossible to identify through global measures and may inform future precision geromedicine approaches.

The region-specific molecular signatures observed here can be interpreted in the context of established mechanisms of brain-periphery communication during aging. Communication between the brain and peripheral circulation is bidirectional and is mediated by multiple routes, including transmigration across the blood-brain barrier (BBB), signaling through brain endothelial cells, and exchange at the choroid plexus.^7^^,^^14^ The BBB regulates the transport of molecules into and out of the central nervous system, and neuroimaging evidence in aging humans has demonstrated regional BBB permeability changes, most notably in the hippocampus.^33^^,^^34^ Systemic factors including chronic inflammation, vascular dysregulation, and metabolic disturbances have been positioned as upstream mediators of brain homeostasis and age-related change.^35^ Regional brain aging patterns may, therefore, reflect the interaction between shared systemic aging processes and region-specific vulnerabilities, with motor cortex microvasculature, medial temporal protein clearance, and cerebellar iron handling each subject to distinct patterns of vulnerability that could plausibly generate the region-specific plasma protein signatures observed here. Because our findings are based on cross-sectional correlations rather than direct measurement of blood-brain protein exchange, these mechanistic interpretations remain hypothesis generating, and further work incorporating direct assessment of regional BBB permeability and brain-derived plasma proteins would be needed to establish causal pathways.

Longitudinal characterization in 444 participants with repeated neuroimaging revealed substantial heterogeneity in brain aging trajectories, extending cross-sectional heterogeneity findings to temporal dynamics while relating phenotypes to proteomic signatures and cognitive outcomes. Recent studies identified heterogeneous aging patterns using latent class analysis and machine learning,^18^ but longitudinal phenotyping with integrated proteomic characterization remained unexplored. In this longitudinal subgroup, females comprised the majority of delayed aging participants but a minority of accelerated aging individuals, consistent with evidence that males exhibit faster brain structural decline^36^ and sex-specific glial responses to inflammaging.^29^ Accelerated aging conferred elevated cognitive decline risk (HR = 1.558 vs. normal aging), aligning with associations between accelerated atrophy trajectories and cognitive deterioration.^37^^,^^38^ Critically, proteomic profiling identified 25 proteins distinguishing extreme phenotypes, with delayed aging characterized by elevated immunoglobulins (IGLV4_60, IGHV4_4, and IGLC7) and serum amyloid A2 suggesting preserved adaptive immune responses,^39^ while accelerated aging demonstrated elevated endothelial dysfunction markers and complement regulatory proteins. These divergent molecular signatures indicated fundamentally different biological states rather than merely different rates along a common trajectory, differing from existing models that position brain aging as uniform acceleration or deceleration. This phenotype-proteomic integration enables personalized monitoring, with accelerated agers requiring intensive surveillance and targeted interventions addressing their specific endothelial-immune dysregulation profile.

The present findings position regional neuroimaging-proteomic integration as superior to either modality alone for mortality prediction and biological characterization. Recent proteomic organ aging studies demonstrated brain age as the strongest single mortality predictor among 11 organs,^17^ with youthful brains conferring 40% mortality risk reduction and aged brains conferring 182% risk increase. While these findings established plasma proteomics as a scalable brain aging tool, our direct neuroimaging assessment achieved three critical advances: (1) superior discriminative performance through regional pattern optimization specifically for mortality (C-index 0.81 vs. published global brain age C-indices of 0.77–0.79), (2) revelation of region-specific pathways (motor cortex coagulation, medial temporal proteostasis, and cerebellar iron homeostasis) undetectable through blood-based global measures, and (3) characterization in an East Asian cohort, extending predominantly European-ancestry findings. Beyond mortality prediction, our integration addressed gaps in existing heterogeneity research. While Yang et al. identified five atrophy patterns requiring complex deep learning,^18^ our three-region model achieved superior mortality discrimination with anatomically interpretable, clinically implementable biomarkers. Previous longitudinal brain aging studies examined trajectories^38^ or cross-sectional patterns,^18^ but not their proteomic underpinnings. Our phenotype-proteomic integration revealed that aging heterogeneity reflected distinct biological processes requiring phenotype-specific interventions. This multi-scale framework established neuroimaging-proteomic integration as essential for precision geromedicine, with neither modality alone capturing the additional biological detail achieved through their combination.

Circulating proteomic biomarkers associated with regional brain aging enable blood-based risk assessment without neuroimaging requirements, facilitating population-scale screening while preserving biological interpretability. Combined with recent demonstrations that plasma proteomics can estimate organ-specific aging, our region-specific pathway characterization suggests a hybrid strategy: initial blood-based screening to identify high-risk individuals, followed by targeted neuroimaging to characterize the affected brain regions and intervention planning. Regional specificity indicates that system-targeted interventions may prove superior to global approaches—motor cortex vulnerability suggests vascular optimization through blood pressure control, medial temporal proteostasis dysfunction indicates protein clearance enhancement via autophagy induction, and cerebellar iron dysregulation points to antioxidant or chelation strategies. Critical research priorities include: (1) validating regional brain age signatures across diverse populations, (2) examining causal relationships through intervention trials targeting identified pathways, (3) developing simplified blood-based algorithms for scalable screening, and (4) testing whether interventions modifying regional brain age translate to mortality benefit. Success would transform brain aging from an observational biomarker into an actionable clinical target, enabling biologically informed risk stratification, mechanistically guided interventions, and personalized monitoring to extend health span.

### Limitations of the study

Several considerations informed interpretation and future directions. This single-cohort Taiwanese study requires validation across diverse populations to establish the generalizability of regional signatures and proteomic profiles. Proteomic data availability in approximately half the cohort, with further limitation in extreme phenotype characterization, constrained statistical power for molecular discovery. Given the relatively low mortality rate observed in this cohort, we were unable to examine associations with cause-specific mortality, which would require longer follow-up. The longitudinal cohort exhibited a healthy-volunteer attrition pattern, with returning participants being younger and healthier at baseline than non-returning participants. Because the aging phenotype classification was defined relative to the longitudinal sample distribution, associations reported for accelerated aging should be interpreted within this returning subgroup, and effect estimates in the broader baseline population may differ in magnitude. In addition, our mass spectrometry-based untargeted proteomic profiling preferentially quantified higher-abundance plasma proteins and did not include several low-abundance signaling proteins previously identified as brain age markers, such as GDF15, GFAP, and FGF21. A systematic coverage analysis further indicated that the measured panel was significantly enriched for extracellular-space proteins but under-represented for intracellular, nuclear, and plasma membrane proteins, as well as several regulatory protein classes (Table S9). This platform-related coverage difference likely accounts for the limited individual-protein overlap with prior brain age proteomic studies. Nevertheless, pathway-level convergence with previous findings is consistent with the inflammatory and immune pathways identified in our analysis, and future studies incorporating complementary proteomic platforms could extend individual-protein-level comparisons. The present brain age modeling was based on gray matter volume derived from T1-weighted MRI. Multimodal extensions incorporating white matter integrity, diffusion metrics, or tract-based connectivity between mortality-associated regions could enrich the biological granularity of brain age estimation and represent a valuable direction for future studies. Alcohol consumption in the present study was ascertained as a binary variable (ever vs. never), which does not distinguish between moderate and excessive drinking patterns. Although alcohol consumption was included as a covariate in the fully adjusted models, this crude categorization may not fully capture the confounding influence of alcohol on brain aging. More broadly, the regional biomarker and proteomic associations reported throughout this study represent statistical correlates of regional brain aging rather than validated mechanisms, and establishing the underlying biological pathways would require complementary experimental validation. The regional patterns described here should accordingly be interpreted as hypothesis generating rather than as mechanistic evidence. Recent advances in multi-omic aging clocks^27^ and organ-specific aging indices^17^ suggest future integration with epigenetic, metabolomic, and additional proteomic platforms as a direction for further characterizing these associations. Despite these limitations, convergent findings across neuroimaging, molecular, and longitudinal analyses provide a regionally resolved and hypothesis-generating characterization of brain aging heterogeneity and its association with mortality.

## Resource availability

### Lead contact

Requests for further information and resources should be directed to and will be fulfilled by the lead contact, Liang-Kung Chen (lkchen2@vghtpe.gov.tw).

### Materials availability

This study did not generate new unique reagents.

### Data and code availability

• Plasma proteomic data reported in this paper have been deposited to the MassIVE repository via ProteomeXchange with the dataset identifiers MassIVE: MSV000103015 and ProteomeXchange: PXD083183. Because of privacy and ethical restrictions on individual-level human cohort data, raw image data are not publicly deposited. Requests for individual-level raw data will be considered by the lead contact, Liang-Kung Chen (lkchen2@vghtpe.gov.tw), when accompanied by a justified scientific proposal and documented ethical approval.
• Neuroimaging and machine learning analyses, including brain age prediction modeling, were implemented in Python and MATLAB using established open-source packages, including SPM12 for neuroimaging preprocessing and scikit-learn for support vector regression modeling, without development of new algorithms or custom analytical code.
• Any additional information required to reanalyze the data reported in this paper is available from the lead contact upon request.

## Acknowledgments

This study was supported by the 10.13039/100020595National Science and Technology Council (NSTC 115-2321-B-A49-011 to L.-K.C., 115-2321-B-A49-013 to C.-P.L., and 115-2314-B-A49-072-MY2 to P.-L.L.), the 10.13039/501100011912Taipei Veterans General Hospital (115VACS-001), 10.13039/100021037Taipei City Hospital (TPCH-115-05), and the Department of Health, Taipei City Government (11501-62-021). The authors thank the Interdisciplinary Research Center for Healthy Longevity of National Yang Ming Chiao Tung University from The Featured Areas Research Center Program within the framework of the Higher Education Sprout Project by the Ministry of Education (MOE) in Taiwan for supporting the research work.

## Author contributions

Conceptualization, L.-K.C.; methodology, P.-L.L. and Y.-L.H.; investigation, P.-L.L. and Y.-L.H.; resources, C.-K.L., W.-J.L., L.-N.P., F.-Y.H., C.-P.L., and L.-K.C.; data curation, W.-J.L. and L.-N.P.; writing – original draft, P.-L.L. and Y.-L.H.; writing – review and editing, L.-K.C.; visualization, P.-L.L.; supervision, C.-K.L., W.-J.L., F.-Y.H., C.-P.L., and L.-K.C.; project administration, L.-K.C.; funding acquisition, C.-P.L. and L.-K.C.

## Declaration of interests

The authors declare no competing interests.

## Declaration of generative AI and AI-assisted technologies in the writing process

During the preparation of this work, the authors used Claude (Anthropic) to assist with English language editing and refinement during manuscript preparation. After using this tool, the authors reviewed and edited the content as needed and take full responsibility for the content of the publication.

## STAR★Methods

### Key resources table

**Table:** 

REAGENT or RESOURCE	SOURCE	IDENTIFIER
**Biological samples**		

Human plasma samples	I-Lan Longitudinal Aging Study (ILAS), Taiwan	N/A

**Chemicals, peptides, and recombinant proteins**		

Acetonitrile, LiChrosolv	Merck	Cat# 1.00029
Formic acid, LiChropur	Supelco (Merck)	Cat# 5.43804
Dithiothreitol	Sigma	Cat# D0632
iodoacetamide	Sigma	Cat# I6125

**Critical commercial assays**		

SMART Digest Kit with SOLAμ HRP SPE plate	Thermo Fisher Scientific	Cat# 60109–103

**Deposited data**		

Plasma proteomic data	This paper	MassIVE: MSV000103015; ProteomeXchange:PXD083183

**Software and algorithms**		

Proteome Discoverer version 2.4	Thermo Fisher Scientific	https://www.thermofisher.com/tw/zt/home/industrial/mass-spectrometry/liquid-chromatography-mass-spectrometry-lc-ms/lc-ms-software/multi-omics-data-analysis/proteome-discoverer-software.html
MATLAB R2021a	The MathWorks	https://matlab.mathworks.com/
SPM12 version 7487	Open source	https://www.fil.ion.ucl.ac.uk/spm/software/spm12/
Python version 3.11	Open source	https://www.python.org/
scikit-learn version 1.6.1	Open source	https://scikit-learn.org/stable/
SPSS version 29	IBM	https://www.ibm.com/products/spss-statistics
JASP version 0.95.1	JASP Team	https://jasp-stats.org/
DAVID Bioinformatics Resources	National Institute of Allergy and Infectious Diseases (NIAID), NIH	https://davidbioinformatics.nih.gov/

**Other**		

Schaefer 400-region cortical atlas	Schaefer et al.^40^	https://github.com/ThomasYeoLab/CBIG/tree/master/stable_projects/brain_parcellation/Schaefer2018_LocalGlobal
Harvard-Oxford subcortical atlas	Makris et al.^41^ Frazier et al.^42^	https://fsl.fmrib.ox.ac.uk/fsl/docs/other/datasets.html
SUIT cerebellar atlas	Diedrichsen^43^	https://github.com/DiedrichsenLab/cerebellar_atlases

### Experimental model and study participant details

#### Study population

We analyzed participants from the I-Lan Longitudinal Aging Study (ILAS), an ongoing community-based cohort established in 2011.^44^ The ILAS recruited community-dwelling adults aged 50 years or older from Yuanshan Township, I-Lan County, Taiwan. Initial enrollment occurred between January 2011 and July 2014, comprising 1,839 participants. At baseline (ILAS-1), participants underwent brain MRI, blood sampling for biomarkers and proteomics, cognitive testing, physical assessments, and clinical interviews. Smoking and alcohol drinking status were ascertained through self-report questionnaires and categorized as ever (current or former) versus never users. The complete participant selection flowchart was shown at Figure S2. Among 1,839 initially enrolled adults, we excluded 599 participants due to: existing neurological or psychiatric disorders (stroke, dementia, brain tumor, major depression, substance abuse), suboptimal MRI quality, MRI contraindications (claustrophobia, ferromagnetic implants), or incomplete baseline data. The final baseline cohort consisted of 1,240 participants with adequate brain MRI scans and complete assessments for brain age analysis. Sex was included as a covariate in all mortality and biomarker analyses, and sex-stratified associations and sex-by-BAG interaction effects are reported in the Results.

Vital status was ascertained through telephone interviews conducted every three months by trained research nurses. Follow-up extended from baseline enrollment until April 2022. Between 2018 and 2024, we invited participants to return for follow-up assessments (ILAS-3) that included repeated brain MRI and cognitive testing. Among those who returned, 444 participants completed both assessments with optimal MRI quality, forming the longitudinal cohort for analyzing brain aging trajectories. Baseline characteristics of returning and non-returning participants are compared in Table S10.

All participants provided written informed consent. The study protocol received approval from the Institutional Review Boards of National Yang Ming Chiao Tung University (YM103008) and Taipei Veterans General Hospital (2018-05-003 B). All procedures adhered to the principles outlined in the Declaration of Helsinki.

### Method details

#### Brain MRI acquisition

All brain MRI scans were acquired at National Yang Ming Chiao Tung University, Taipei, Taiwan. Baseline T1-weighted anatomical scans (ILAS-1, *n* = 1,240) were obtained on a Siemens 3 T Magnetom Tim Trio scanner (Siemens Healthcare, Erlangen, Germany) using a 12-channel phased-array head coil. Images were acquired using a three-dimensional magnetization-prepared rapid-acquisition gradient-echo (3D MPRAGE) sequence with the following parameters: repetition time/echo time/inversion time = 3500/3.5/1100 ms, flip angle = 7°, field of view = 256 × 256 mm^2^, matrix size = 256 × 256, number of excitations = 1, 192 sagittal slices with isotropic 1 mm^3^ resolution and no interslice gap.

During the longitudinal follow-up (ILAS-3, 2018–2024), the scanner was upgraded from a Magnetom Tim Trio to a Magnetom Prisma^Fit^ in 2021. Among 444 participants with repeat MRI, 253 were scanned on the original Trio system using the identical baseline protocol, while 191 were scanned on the upgraded Prisma Fit system using the same 3D MPRAGE sequence with modified repetition time (TR = 2500 ms; all other parameters unchanged). Comparison of brain metrics between these two groups confirmed systematic differences attributable to scanner upgrade (Table S11), necessitating appropriate correction in longitudinal analyses.

All anatomical scans underwent visual quality inspection by an experienced neuroradiologist to exclude participants with incidental findings (such as brain tumors, traumatic lesions, or large territorial infarcts) or inadequate image quality resulting from excessive head motion or acquisition artifacts. Participants with poor-quality scans were excluded from the analysis, as described in the Study Population section.

#### Clinical biomarkers and physical function assessments

Following a minimum 10-h overnight fast, peripheral blood samples were collected from all participants between 7:00 and 9:00 a.m. Samples were processed and analyzed using standard laboratory protocols for a comprehensive panel of 46 biomarkers spanning seven physiological domains: metabolic regulation (lipid profile, glucose homeostasis), endocrine function (sex hormones, growth factors), inflammation and immune status (acute-phase proteins, white blood cell differential), hepatic function (transaminases, synthetic markers), renal function (nitrogenous waste products, electrolytes), nutritional status (vitamins, micronutrients), and hematologic parameters (complete blood count indices). The complete list of biomarkers is provided in Table S5.

Physical performance was evaluated through three standardized assessments. Handgrip strength was measured using a digital dynamometer (Smedlay’s Dynamo Meter; TTM, Tokyo, Japan) with participants in an upright position using their dominant hand. Gait speed was assessed over a 6-meter walk at the participant’s usual pace. Relative appendicular skeletal muscle mass (RASM) was derived from whole-body dual-energy X-ray absorptiometry scans, calculated as the sum of fat-free lean mass from all four limbs divided by height squared (kg/m^2^).

#### Proteomic analysis

Baseline plasma samples from 606 participants with analyzable plasma proteomic data were analyzed. Comparison of baseline characteristics between participants with and without proteomic data is provided in Table S12. Fasting peripheral blood was collected in EDTA-coated tubes and centrifuged at 3,000 g for 10 min at 4°C. Plasma was separated and stored at −80°C until analysis. Protein digestion was performed using the SMART Digest Trypsin kit (Thermo Fisher Scientific). Each 10 μL plasma sample was diluted with digestion buffer and incubated at 70°C for 2 h to achieve complete tryptic digestion. Digested peptides underwent reduction, alkylation with iodoacetamide, and purification through solid-phase extraction using SOLAμ plates. Samples were subsequently desalted with C18 ZipTip (Merck) prior to mass spectrometry analysis.

Peptide separation and analysis were conducted using nano-liquid chromatography coupled to an Orbitrap Elite mass spectrometer (Thermo Fisher Scientific). Peptides were eluted over 210 min using a segmented gradient on a C18 column. Mass spectrometry data were acquired in data-dependent mode with 60-s dynamic exclusion.

Raw spectral data were processed using Proteome Discoverer version 2.4 (Thermo Fisher Scientific) with database searches performed against the human UniProt database using both Mascot and Sequest HT algorithms. Search parameters allowed one missed tryptic cleavage site, with carbamidomethylation of cysteine as a fixed modification and methionine oxidation as a variable modification. Mass tolerance was set at 20 ppm for precursor ions and 0.8 Da for fragment ions. Protein quantification was based on MS1-level intensities, calculated as the sum of unique peptide abundances. Data normalization utilized the total peptide amount across all samples. Proteins detected in fewer than 50% of samples were excluded, resulting in 513 proteins for statistical analysis.

#### Cognitive assessments

Cognitive function was evaluated through a standardized neuropsychological battery administered by trained research personnel during face-to-face interviews. Global cognitive performance was assessed using the Mini-Mental State Examination (MMSE). Domain-specific cognitive abilities were assessed using five tests that spanned four cognitive domains. Memory function was evaluated through the 10-min delayed recall component of the Chinese Version Verbal Learning Test (CVVLT).^45^ Language ability was assessed with the Boston Naming Test (BNT).^46^ Visuospatial function was measured using the copy task from the Taylor Complex Figure Test (TCF).^47^ Executive function was evaluated through the Backwards Digit Test (BDT).^48^ The Clock Drawing Test (CDT) assessed both visuospatial and executive functions.^49^^,^^50^

For longitudinal analysis in the subset with repeat assessments (*n* = 444), cognitive decline was operationally defined as performance deterioration exceeding the 90th percentile (i.e., a top 10% decline) in any of the five domain-specific tests (CVVLT, BNT, TCF, BDT, CDT). Change scores were calculated as the difference between follow-up and baseline performance for each test. Participants who demonstrated decline in one or more tests were classified as having cognitive decline for subsequent survival analysis.

#### Image processing and feature extraction

Individual T1-weighted images from both the ILAS cohort and an independent training dataset (see “Brain Age Prediction Model” section, *n* = 1,482) were preprocessed using a voxel-based morphometry pipeline implemented in Statistical Parametric Mapping 12 (SPM12, version 7487; Wellcome Trust Center for Neuroimaging, University College London, UK) within MATLAB R2021a (MathWorks, Natick, MA, USA). The complete neuroimaging processing and analysis workflow is illustrated in Figure S3. The preprocessing workflow employed a high-dimensional Diffeomorphic Anatomical Registration Through Exponentiated Lie Algebra (DARTEL) approach, following protocols established and validated in our previous studies.^51^ The pipeline comprised five sequential steps: (1) bias field correction to address intensity inhomogeneities; (2) tissue segmentation to classify gray matter, white matter, and cerebrospinal fluid; (3) construction of a study-specific tissue template from the training dataset, to which both training and ILAS images were spatially normalized and subsequently aligned to Montreal Neurological Institute (MNI) standard space; (4) tissue modulation to preserve absolute tissue volumes following spatial transformation; and (5) spatial smoothing with a 6-mm full-width at half-maximum Gaussian kernel. To account for individual differences in overall brain size, global tissue volume, and total intracranial volume (TIV = gray matter + white matter + cerebrospinal fluid volumes) were estimated for each participant in native space.

Regional gray matter features were extracted using a composite brain atlas comprising 442 regions of interest. Cortical parcellation utilized the Schaefer 400-region functional atlas,^40^ which delineates the cerebral cortex into seven large-scale networks (visual, somatomotor, dorsal attention, ventral attention/salience, limbic, central executive, and default mode) based on intrinsic functional connectivity patterns. Subcortical structures were defined using 14 regions from the Harvard-Oxford subcortical atlas,^41^^,^^42^ and cerebellar parcellation employed 28 regions from the spatially unbiased infratentorial template.^43^ Two types of feature sets were extracted from the resultant gray matter volume images. For global brain age modeling, mean gray matter volume was computed for each of the 442 regions by averaging values across all voxels within that region. For regional brain age modeling, voxel-wise gray matter volumes within each region were retained as separate features, enabling region-specific age prediction models. The voxel count of each region is listed in Table S13.

#### Brain age prediction model

##### Training dataset

To develop robust brain age prediction models, we compiled T1-weighted anatomical scans from 1,482 healthy participants (age range 18–92 years; 681 males, 801 females) recruited across five research sites in Taiwan utilizing six MRI scanners. Participants were free from neuropsychiatric disorders, head trauma, and major medical conditions. Site-specific scanner specifications and imaging parameters are detailed in Table S14 and Figure S4A.

##### Model architecture and training

We employed support vector regression with radial basis function kernel (RBF-SVR) to construct global and regional brain age prediction models, following the validated framework established in our previous study.^51^ The brain age model was trained on the combined sample of male and female participants, consistent with established conventions in brain age modeling^9^^,^^52^ and the sample size requirements demonstrated in recent large-scale evaluations^53^^,^^54^; sex was not entered as a model input but was incorporated as a covariate in all downstream mortality and biomarker analyses. Model training utilized nested 5-fold cross-validation, wherein the inner loop performed hyperparameter optimization (C and gamma parameters) via GridSearchCV across the search space [0.001, 0.01, 0.1, 1, 10, 100, 1000], while the outer loop evaluated model performance using mean absolute error (MAE) and coefficient of determination (R^2^) (Figures S4B and S4C). To assess whether regional model performance was influenced by region size, Spearman correlations between voxel count and prediction accuracy were examined across the 442 regional models. Although statistically significant, the correlations were weak (MAE: rho = −0.12, *p* = 0.010; R^2^: rho = 0.13, *p* = 0.006), indicating that region size explained less than 2% of the variability in regional model performance. Voxel counts and prediction performance metrics for all 442 regions are provided in Table S13. Final models trained on the entire training dataset were then applied to generate brain age predictions for all ILAS participants (*n* = 1,240 at baseline and *n* = 444 at longitudinal follow-up).

##### Age-bias correction and brain age gap calculation

Brain age prediction models inherently exhibit estimation bias due to “regression toward the mean,” whereby younger individuals’ brain ages tend to be overestimated, and older individuals' brain ages tend to be underestimated. To address this age-dependent bias, we implemented a systematic correction procedure following the established method.^9^ Specifically, a linear regression model was fitted to the training dataset to characterize the relationship between predicted brain age and chronological age (predicted age = α × chronological age + β). The resulting regression parameters (α and β) were then used to derive corrected brain age estimates for all ILAS participants via the formula: corrected brain age = (predicted age − β)/α. The individual brain age gap (BAG) was calculated as the difference between the corrected brain age and chronological age.^52^ Both global and regional BAG metrics were computed for subsequent analyses.

#### Longitudinal brain aging phenotypes

A subset of 444 participants underwent repeat brain MRI and cognitive assessments during longitudinal follow-up (ILAS-3). To characterize overall brain aging trajectories at the individual level and ensure comparability with existing longitudinal brain age literature, we used global BAG to define aging phenotypes.^10^^,^^38^ For each participant, we calculated the change in brain age gap (ΔBAG) as the difference between follow-up and baseline BAG values (ΔBAG = BAG_follow-up_ − BAG_baseline_). The annual rate of brain age gap change (BAG slope) was then computed by dividing ΔBAG by the individual inter-scan interval in years.

These participants were classified into three brain aging phenotypes based on the distribution of BAG slopes across the longitudinal cohort. To account for potential confounding effects of baseline BAG and MRI scanner upgrade (191 of 444 participants scanned on upgraded scanner at follow-up), we adjusted the BAG slope using a general linear model with baseline BAG and scanner upgrade status as covariates, and the resulting residuals were *Z* score standardized. Participants were classified into three brain aging phenotypes based on the distribution of adjusted z-scores. Delayed Aging (DA) was defined as *Z* score < −1 (equivalent to adjusted BAG slope <mean − 1 standard deviation), indicating brain aging slower than expected given baseline status. Normal Aging (NA) was defined as −1 ≤ *Z* score ≤ +1, reflecting typical aging trajectories. Accelerated Aging (AA) was defined as *Z* score > +1, indicating brain aging faster than expected given baseline status.

### Quantification and statistical analysis

#### Descriptive statistics and group comparisons

Baseline characteristics of the cross-sectional cohort were summarized using descriptive statistics. Continuous variables were presented as mean ± standard deviation, and categorical variables as frequencies and percentages (n, %). Group comparisons were performed to characterize mortality-associated baseline factors and brain age gap distributions. Comparisons between survival status (alive vs. death) assessed baseline predictors of mortality. Demographic and lifestyle variables were compared using Student’s *t* test for continuous variables and chi-square test for categorical variables. Variables potentially confounded by demographic factors were compared using analysis of covariance (ANCOVA), with physical function measures adjusted for age and sex; cognitive scores for age, sex, and education; and global brain age gap and brain imaging metrics for age, sex, and TIV. Global brain age gap distributions across cardiovascular risk profiles were evaluated by comparing BAG between participants with and without major risk factors (hypertension, diabetes mellitus, smoking, alcohol consumption) using ANCOVA adjusted for age, sex, and TIV. Statistical significance was defined as two-tailed *p* < 0.05. Analyses were performed using IBM SPSS Statistics version 29 (IBM Corp., Armonk, NY, USA) and MATLAB R2021a (MathWorks, Natick, MA, USA).

#### Global and regional BAG associations with mortality risk

Cox proportional hazards regression models assessed associations between brain age gaps and all-cause mortality. Time-to-event was defined from baseline MRI to death or censoring (study end, April 2022). Minimally adjusted model included age, sex, and TIV; Fully adjusted model additionally adjusted for cardiovascular and lifestyle risk factors, including education years, hypertension, diabetes, smoking, and alcohol drinking habits. To assess whether the BAG–mortality association was modified by sex, an additional Cox model was fitted incorporating a BAG × sex interaction term in the fully adjusted specification. Proportional hazards assumptions were verified using Schoenfeld residuals tests. Regional mortality associations were identified through univariate Cox regression for each of 442 regional BAGs with Bonferroni correction (α = 0.05/442 = 1.13 × 10^−4^). Forward stepwise selection based on likelihood ratio tests identified an optimal regional model, starting with base covariates and sequentially adding regional BAGs with significant improvement in model fit. Model performance was evaluated using Harrell’s concordance index (C-index, calculated using JASP version 0.95.1; JASP Team, Amsterdam, Netherlands) across models incorporating different combinations of global BAG, optimal regional BAGs, and covariates.

#### Molecular correlates of brain aging

Associations between brain age gaps (global and three optimal regional BAGs) and molecular biomarkers were evaluated using partial Spearman correlation adjusted for age, sex, and TIV. Two sets of correlation analyses were performed: (1) 55 baseline assessments comprising 46 blood markers, 3 physical function assessments, and 6 cognitive test scores (complete list provided in Table S5) with false discovery rate correction (q < 0.05) applied within each brain age measure; and (2) 513 plasma proteins (*n* = 606) without multiple comparison correction given the exploratory nature. Plasma protein data were log-transformed and *Z* score standardized prior to statistical analysis. To elucidate biological processes underlying protein-brain age associations, pathway enrichment analysis was performed using the Database for Annotation, Visualization and Integrated Discovery (DAVID; https://david.ncifcrf.gov/), with Gene Ontology biological process as the primary annotation category. To assess robustness across pathway annotation frameworks, cross-database validation was additionally performed using KEGG, Reactome, and WikiPathways.

#### Longitudinal brain aging phenotypes and cognitive outcomes

Baseline characteristics were compared across the three aging phenotypes (delayed, normal, accelerated) using analysis of variance for continuous variables and chi-square tests for categorical variables, with analysis of covariance adjustment for brain metrics (age, sex, TIV, MRI scanner type). Cox proportional hazards regression assessed associations between aging phenotypes and risk of cognitive decline during follow-up, with the normal aging group as reference. Hazard ratios with 95% confidence intervals were reported. Kaplan-Meier curves were used to visualize cognitive decline-free survival across phenotypes. To identify molecular signatures distinguishing aging phenotypes, plasma protein levels were compared between accelerated and delayed aging groups (*n* = 31 vs. *n* = 40) using analysis of covariance adjusted for baseline age and sex. Statistical significance was defined at *p* < 0.05.

## Supplemental information

Document S1. Figures S1–S4 and Table S1, S4–S6, S8–S12, and S14

Table S2. Minimally adjusted Cox PH association between regional brain age gap and mortality, related to Figure 2

Table S3. Retained Cox PH association between regional brain age gap and mortality after adjustment for age, sex, and other risk factor covariates, related to Figure 2

Table S7. Cross-database pathway enrichment analysis of brain age-associated proteins, related to Figure 4

Table S13. Voxel counts and prediction performance for all 442 regional brain age models, related to STAR Methods

Document S2. Article plus supplemental information
