# Supplementary material for: Regional brain aging signatures predict mortality through neurovascular immune mechanisms
Source: Cell Rep Med. 2026 Oct 7;7(10):103096. doi: 10.1016/j.xcrm.2026.103096 (PMC13710373; doi:10.1016/j.xcrm.2026.103096)
Supplement: Supplementary file 1 [file mmc1.pdf]

Cell Reports Medicine, Volume *7*

Supplemental information

Regional brain aging signatures predict mortality through neurovascular immune mechanisms

Pei-Lin Lee, Yi-Long Huang, Chih-Kuang Liang, Wei-Ju Lee, Li-Ning Peng, Fei-Yuan Hsiao, Ching-Po Lin, and Liang-Kung Chen

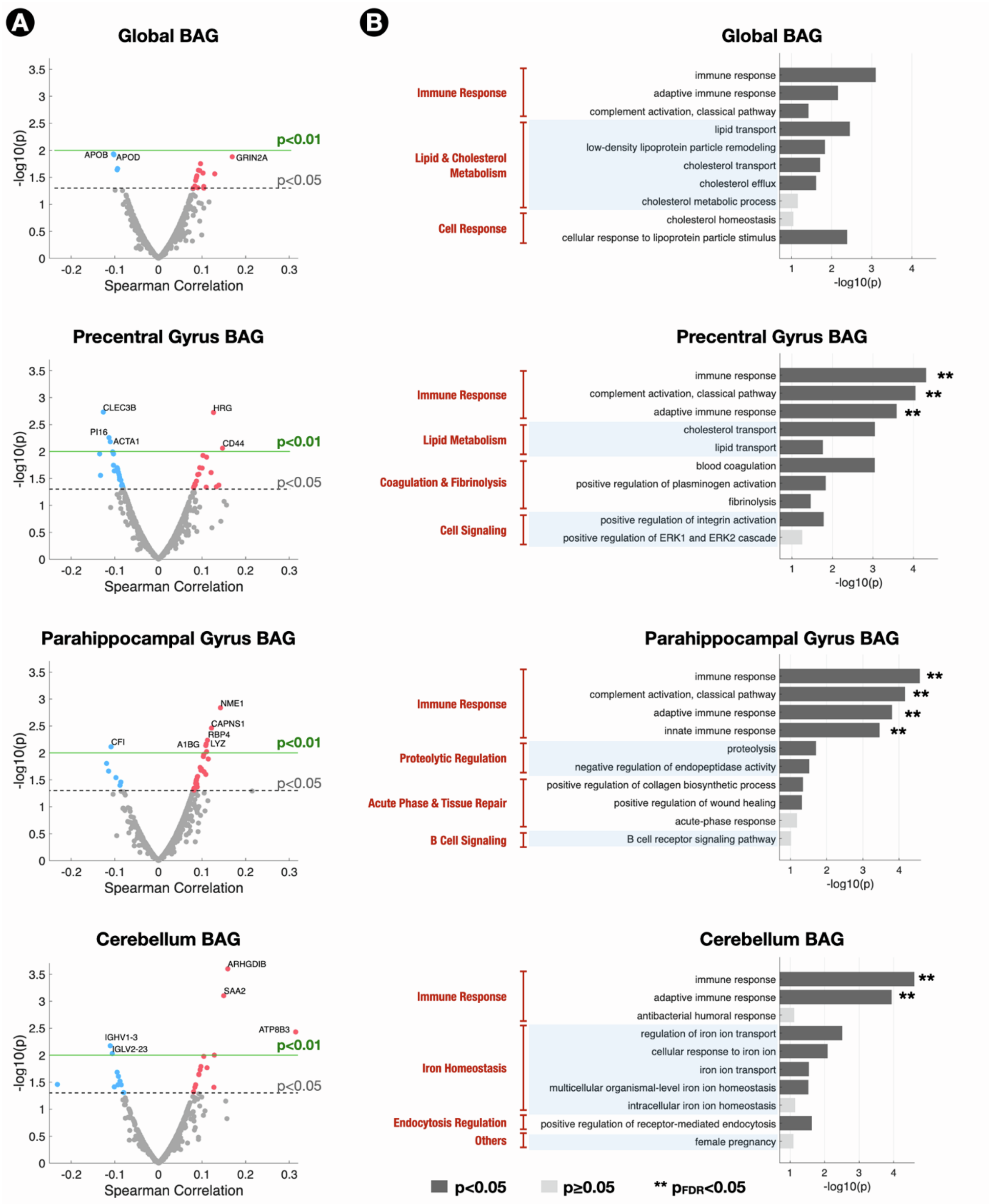

Figure S1. Proteomic Correlates of Global and Regional Brain Age Gaps. Related to Figure 4.

(A) Association between plasma proteins and brain age gaps (n=606 participants). Each panel displays Spearman correlations (x-axis) versus statistical significance (y-axis, $-\log_{10}$ p-value) for approximately 500 quantified proteins. All correlations adjusted for age, sex, and TIV. Blue points indicate negative correlations; red points indicate positive correlations; gray points indicate non-significant associations (p≥0.05). Horizontal dashed lines mark significance thresholds (p=0.05, gray; p=0.01, green). (B) The representative biological processes of GO enrichment analyses. A summary of the terms is provided to the left (red labels). Dark gray bars indicate p<0.05; light gray bars indicate p≥0.05. ** indicate p<0.05 after false discovery rate correction.

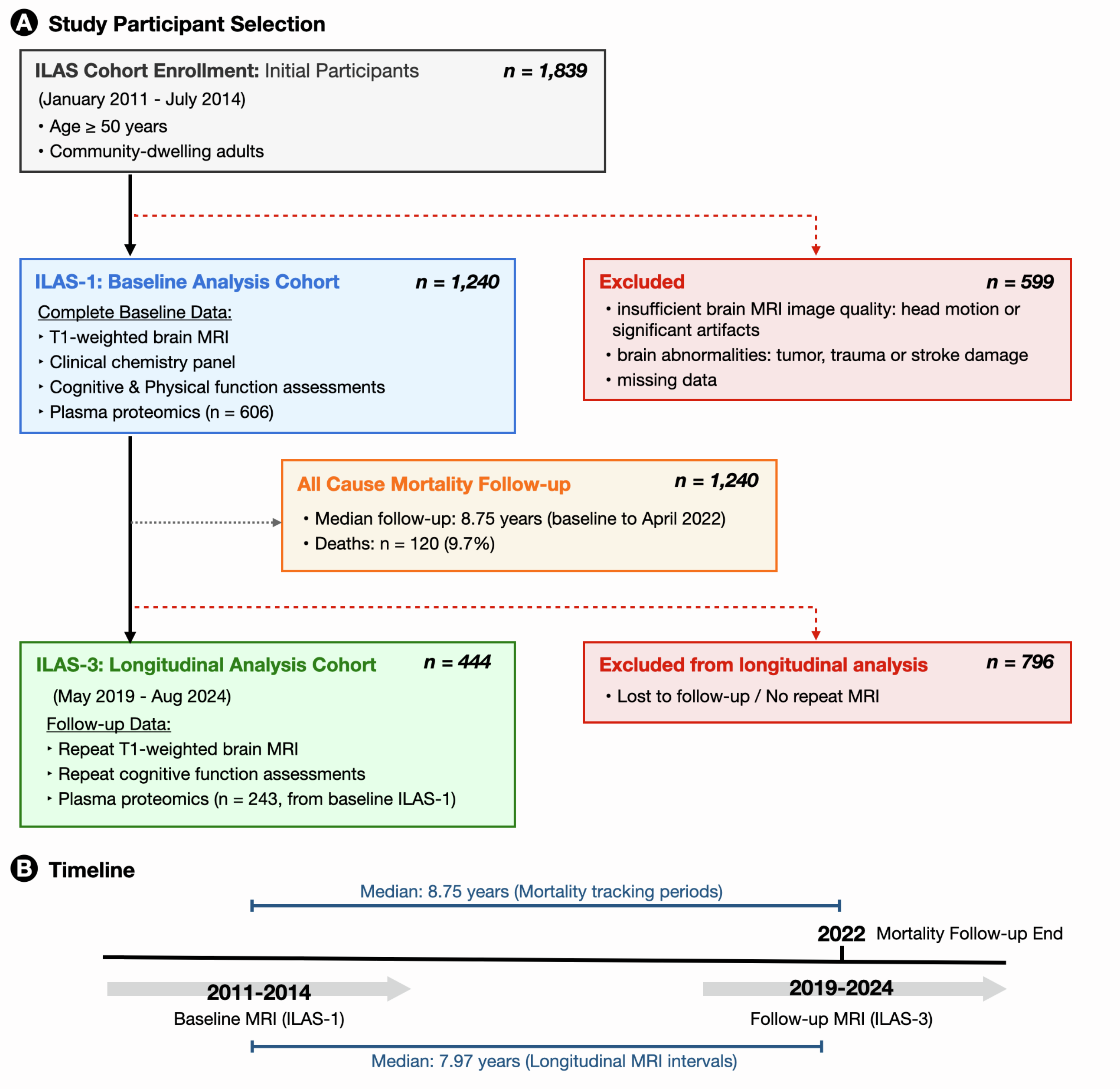

Figure S2. Study population recruitment. Related to STAR Methods.

Flowchart depicting participant selection through ILAS study phases. Phase 1 (2011-2014): Initial enrollment of 1,839 community-dwelling adults. After excluding 599 participants (poor MRI quality, neurological conditions, missing data), the baseline cohort comprised 1,240 participants (ILAS-1) with complete brain MRI and assessments. Phase 2: All 1,240 participants were followed for mortality (median 8.75 years, 120 deaths by April 2022). Phase 3 (2019-2024): Longitudinal cohort of 444 participants (ILAS-3) completed repeat MRI (median 7.97-year interval). Among these, 243 participants had baseline plasma proteomics data available from ILAS-1 for analysis. The timeline shows study milestones and data-collection periods.

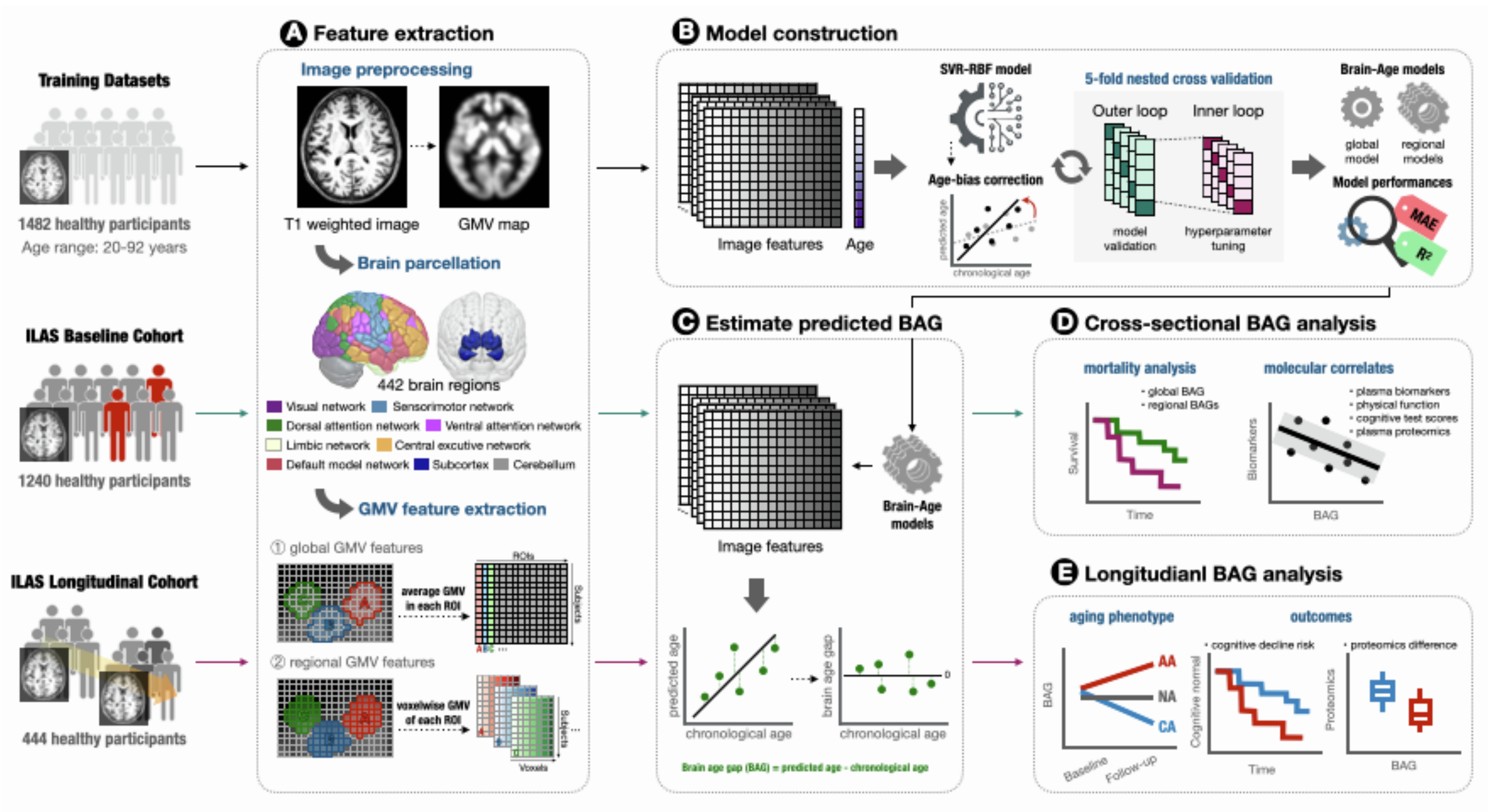

Figure S3. Analysis Framework of the study. Related to STAR Methods.

(A) Structural MRI data were preprocessed using the VBM pipeline in SPM12, and gray matter volume features were extracted globally (averaged across 442 regions) and regionally (voxel-wise within each region). (B) These imaging features were used to construct and validate brain age predictive models using SVR-RBF with nested 5-fold cross-validation in an independent training dataset of 1,482 healthy participants. Age-bias correction was applied, and model performance was evaluated. (C) The established models were applied to ILAS cohorts to estimate individual brain ages, from which brain age gaps were calculated. (D) In the baseline cohort (n=1,240), statistical analyses were conducted to examine associations between global and regional BAG and 10-year mortality risk using Cox regression. Molecular correlates were investigated through associations with clinical biomarkers and plasma proteins. (E) In the longitudinal cohort (n=444), participants were classified into brain aging phenotypes (delayed, normal, accelerated) based on annual rate of BAG change adjusted for baseline BAG and MRI scanner upgrade. Associations between aging phenotypes and cognitive decline risk were examined, and proteomic differences between extreme phenotypes were evaluated.

Abbr. AA, accelerated aging; BAG, brain age gap; DA, delayed aging; GMV, gray matter volume; ILAS, I-Lan Longitudinal Aging Study; MAE, mean absolute error; NA, normal aging; SVR-RBF, Support Vector Regression with Radial Basis Function kernel.

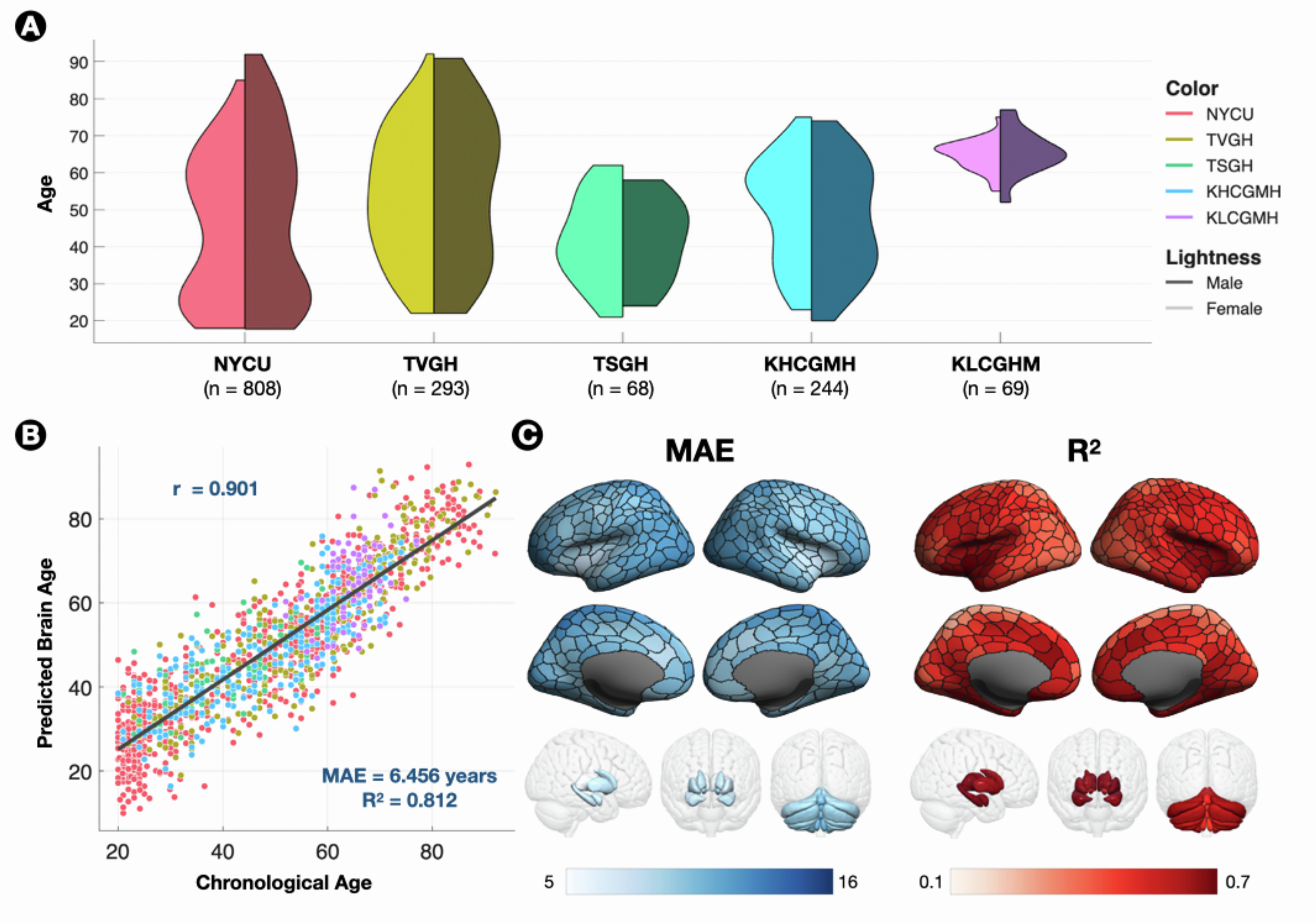

Figure S4. Brain Age Prediction Model Performance. Related to STAR Methods.

Characteristics of the training dataset and performance of the brain age model. (A) Age and sex distribution of the training cohort (n=1,482 healthy participants from 5 research sites). (B) Global brain-predicted age was highly associated with chronological age (r = 0.901, P < 0.001; MAE = 6.46 years and R2 = 0.812). (C) Regional brain-age prediction models also demonstrated moderate to good prediction (MAE range from 5.772 to 13.598 years and R2 range from 0.234 to 0.847).

MAE = mean absolute error; r = correlation coefficient; R2 = coefficient of determination.

Table S1. Baseline characteristics of study samples. Related to Figure 1.

	Cross-sectional samples (n=1240)			p-value
	Overall	Alive	Death	(alive vs. death)
Demographic				
N	1240	1120	120	-
Age, years	63.1 ± 8.8	62.2 ± 8.3	70.9 ± 9.7	<0.001
Female, n (%)	656 (52.9%)	614 (54.8%)	42 (35.0%)	<0.001
Education, years	6.8 ± 5.0	7.1 ± 5.0	4.4 ± 4.9	<0.001
Follow-up time, years	8.66 ± 1.30	8.92 ± 0.82	6.21 ± 2.12	-
Lifestyle and Risk factors				
Smoking , n (%)	210 (16.9%)	171 (15.3%)	39 (32.5%)	<0.001
Alcohol drinking, n (%)	442 (35.6%)	405 (36.2%)	37 (30.8%)	0.271
Hypertension, n (%)	478 (38.5%)	402 (35.9%)	76 (63.3%)	<0.001
Diabetes mellitus, n (%)	188 (15.2%)	157 (14.0%)	31 (25.8%)	<0.001
Physical examination				
Handgrip, kg	29.0 ± 9.5	29.2 ± 9.5	26.8 ± 9.1	<0.001[a]
Gait speed, m/s	1.6 ±0.5	1.6 ± 0.5	1.3 ± 0.5	<0.001[a]
Cognitive performance, scores				
Mini-Mental State Examination	26.2 ± 3.6	26.4 ± 3.5	24.1 ± 4.6	0.041[b]
10-min CVVLT	6.6 ± 2.1	6.7 ± 2.0	5.5 ± 2.5	0.084[b]
Clock drawing test	7.7 ± 2.5	7.8 ± 2.4	6.3 ± 2.9	0.067[b]
Taylor Complex Figure test	30.6 ± 7.1	31.0 ± 6.7	27.0 ± 9.3	0.119[b]
Boston Naming test	12.4 ± 2.5	12.5 ± 2.4	11.3 ± 2.9	0.670[b]
Backward Digit test	3.6 ± 2.0	3.7 ± 1.9	2.7 ± 1.9	0.301[b]
Neuroimaging measurement				
Total intracranial volume, mL	1336 ± 118	1334 ± 119	1358 ± 113	0.471[a]
Gray-matter volume, mL	629 ± 61	631 ± 59	603 ± 69	<0.001[c]
White-matter volume, mL	416 ± 50	417 ± 49	403 ± 53	0.017[c]
Cerebrospinal fluid volume, mL	292 ± 73	285 ± 70	352 ± 66	<0.001[c]
Global brain age gap, years	1.06 ± 9.33	0.63 ± 9.28	5.11 ± 8.89	<0.001[c]

[a]ANCOVA test, adjusted for age and sex.

[b]ANCOVA test, adjusted for age, sex and education years.

[c]ANCOVA test, adjusted for age, sex and total intracranial volume.

Abbreviations: CVVLT = Chinese version Verbal Learning Test

Table S4. Spearman correlations among global BAG and the three optimal regional BAGs (n=1,240). Related to Figure 3 and Table 2.

	Global BAG	Regional BAG in left precentral gyrus	Regional BAG in left parahippocampal gyrus	Regional BAG in right cerebellum I-IV
**Global BAG**	-	0.44	0.43	0.37
**Regional BAG in left precentral gyrus**	0.44	-	0.35	0.29
**Regional BAG in left parahippocampal gyrus**	0.43	0.35	-	0.37
**Regional BAG in right cerebellum I-IV**	0.37	0.29	0.37	-

All correlations p<0.001.

Table S5. Association between brain age metrics and multi-domain biomarker. Related to Figure 4.

Biomarker category	Biomarker Abbr.	Biomarker	Global BAG		Regional BAG in precentral gyrus		Regional BAG in parahippocampal gyrus		Regional BAG in cerebellum I-IV	
			rho	p-value	rho	p-value	rho	p-value	rho	p-value
Metabolic Regulation	TC	Total Cholesterol	-0.032	0.268	-0.026	0.362	-0.058	0.040	-0.044	0.119
Metabolic Regulation	TG	Triglycerides	0.024	0.391	0.053	0.065	-0.005	0.860	0.005	0.857
Metabolic Regulation	A1c	Glycated Hemoglobin	0.079	0.005	0.133	0.000	0.076	0.008	0.051	0.072
Metabolic Regulation	HDL	High-Density Lipoprotein	0.005	0.862	-0.007	0.817	-0.009	0.760	0.011	0.701
Metabolic Regulation	LDL	Low-Density Lipoprotein	-0.051	0.071	-0.053	0.064	-0.051	0.074	-0.049	0.083
Metabolic Regulation	Insulin	Insulin	0.024	0.390	0.053	0.064	0.062	0.028	0.035	0.218
Endocrine	GH	Growth Hormone	0.030	0.290	0.003	0.921	0.041	0.150	0.062	0.029
Endocrine	Testo	Testosterone	-0.016	0.579	-0.039	0.175	0.037	0.190	-0.019	0.504
Endocrine	SHBG	Sex Hormone Binding Globulin	0.033	0.252	-0.013	0.648	-0.018	0.536	-0.008	0.786
Endocrine	FT	Free Testosterone	-0.038	0.177	-0.034	0.233	0.006	0.828	-0.047	0.095
Endocrine	FAI	Free Androgen Index	-0.048	0.092	-0.035	0.217	-0.004	0.882	-0.043	0.133
Endocrine	IGF	Insulin-like Growth Factor	-0.088	0.002	-0.061	0.031	-0.033	0.247	-0.042	0.143
Endocrine	DHEA	Dehydroepiandrosterone	-0.015	0.594	-0.043	0.131	0.011	0.691	-0.048	0.088
Endocrine	iPTH	Intact Parathyroid Hormone	0.000	0.998	0.015	0.601	-0.018	0.523	0.022	0.449
Inflammation & Immune	hsCRP	High-sensitivity C-Reactive Protein	-0.009	0.756	0.008	0.800	0.045	0.136	0.051	0.090
Inflammation & Immune	HCY	Homocystein	0.084	0.003	0.059	0.039	0.079	0.006	0.083	0.004
Inflammation & Immune	WBC	White Blood Cell count	0.087	0.002	0.084	0.003	0.077	0.007	0.011	0.687
Inflammation & Immune	NEUT	Neutrophil	0.096	0.001	0.038	0.180	0.102	0.000	0.029	0.303
Inflammation & Immune	LYMPH	Lymphocytes	-0.109	0.000	-0.055	0.053	-0.101	0.000	-0.032	0.256
Inflammation & Immune	Mono	Monocytes	0.032	0.266	0.058	0.043	0.045	0.114	0.044	0.126
Inflammation & Immune	Eo	Eosinophil	-0.027	0.347	-0.018	0.525	-0.077	0.006	-0.035	0.214
Inflammation & Immune	Baso	Basophil	-0.020	0.483	-0.028	0.327	-0.035	0.223	-0.038	0.184
Hepatic	AST	Aspartate Aminotransferase	-0.024	0.404	-0.023	0.413	0.053	0.065	0.031	0.282
Hepatic	ALT	Alanine Aminotransferase	-0.017	0.554	-0.017	0.562	0.074	0.009	0.022	0.431
Hepatic	ALP	Alkaline Phosphatase	-0.021	0.451	-0.043	0.127	0.028	0.323	0.044	0.121
Hepatic	GGT	Gamma-Glutamyl Transferase	0.066	0.021	0.083	0.004	0.133	0.000	0.109	0.000
Hepatic	ALB	Albumin	-0.027	0.345	0.006	0.824	0.004	0.899	-0.001	0.964
Hepatic	GLO	Globulin	0.013	0.652	-0.003	0.917	0.039	0.172	0.020	0.476

Hepatic	TP	Total Protein	-0.006	0.840	0.005	0.862	0.042	0.141	0.025	0.371
Renal	BUN	Blood Urea Nitrogen	-0.042	0.143	0.011	0.693	-0.036	0.205	0.000	0.999
Renal	UA	Uric Acid	0.038	0.176	0.017	0.546	0.065	0.022	0.101	0.000
Renal	Scr	Serum Creatinine	0.012	0.676	0.033	0.247	0.033	0.240	0.068	0.017
Renal	Ucr	Urine Creatinine	-0.016	0.568	0.041	0.146	0.000	0.993	-0.041	0.148
Renal	Na	Sodium	-0.061	0.032	-0.050	0.081	-0.061	0.031	-0.006	0.827
Renal	K	Potassium	-0.019	0.499	0.000	0.992	0.034	0.226	-0.005	0.867
Renal	mALB	Microalbuminuria	0.109	0.000	0.126	0.000	0.115	0.000	0.070	0.015
Nutritional	B12	Vitamin B12	-0.042	0.138	-0.017	0.556	0.024	0.400	-0.017	0.541
Nutritional	FOL	Folic acid	-0.055	0.053	-0.051	0.073	-0.050	0.077	-0.034	0.230
Nutritional	VitD3	Vitamin D3	0.015	0.600	-0.029	0.314	0.032	0.257	0.031	0.268
Hematologic	RBC	Red Blood Cell count	-0.081	0.005	-0.078	0.006	-0.068	0.017	-0.056	0.051
Hematologic	Hb	Hemoglobin	-0.082	0.004	-0.017	0.554	-0.045	0.112	-0.085	0.003
Hematologic	HCT	Hematocrit	-0.087	0.002	-0.016	0.578	-0.049	0.083	-0.075	0.008
Hematologic	MCV	Mean Corpuscular Volume	0.013	0.658	0.091	0.001	0.035	0.220	0.014	0.612
Hematologic	MCH	Mean Corpuscular Hemoglobin	0.006	0.828	0.060	0.035	0.036	0.202	-0.011	0.703
Hematologic	MCHC	Mean Corpuscular Hemoglobin Concentration	0.040	0.158	0.045	0.111	0.090	0.001	0.016	0.580
Hematologic	PLT	Platelet count	0.048	0.089	0.065	0.022	0.057	0.045	0.018	0.536
Physical function	Grip	Handgrip strength	-0.130	0.000	-0.130	0.129	-0.090	0.002	-0.105	0.000
Physical function	Speed	Gait speed	-0.056	0.051	-0.056	0.347	-0.139	0.000	-0.028	0.332
Physical function	RASM	Relative Appendicular Skeletal Muscle mass	-0.026	0.365	-0.026	0.634	0.023	0.416	0.039	0.172
Cognitive function	MMSE	Mini-Mental State Examination	-0.076	0.008	-0.076	0.115	-0.105	0.000	-0.076	0.007
Cognitive function	CVVLT	Chinese version Verbal Learning Test	-0.036	0.210	-0.036	0.781	-0.065	0.022	-0.040	0.163
Cognitive function	CDT	Clock drawing test	-0.104	0.000	-0.104	0.013	-0.088	0.002	-0.043	0.128
Cognitive function	TCF	Taylor Complex Figure test	-0.130	0.000	-0.130	0.001	-0.112	0.000	-0.106	0.000
Cognitive function	BNT	Boston Naming test	-0.131	0.000	-0.131	0.001	-0.085	0.003	-0.081	0.004
Cognitive function	BDT	Backward Digit test	-0.089	0.002	-0.089	0.028	-0.098	0.001	-0.066	0.020

Partial spearman correlation analysis adjusted age, sex and TIV

Table S6. List of proteins significantly associated with brain age metrics. Related to Figure 4.

Protein	rho	p-value	Protein description
Global BAG			
IGKV2D-30	0.102	0.026	immunoglobulin kappa variable 2D-30
AQR	0.130	0.027	aquarius intron-binding spliceosomal factor
IGLV2-23	-0.094	0.022	immunoglobulin lambda variable 2-23
IGLV3-19	0.096	0.018	immunoglobulin lambda variable 3-19
IGHG2	0.087	0.032	immunoglobulin heavy constant gamma 2 (G2m marker)
APOA2	0.089	0.030	apolipoprotein A2
APOB	-0.103	0.012	apolipoprotein B
IGF1	-0.095	0.023	insulin like growth factor 1
APOD	-0.103	0.012	apolipoprotein D
C8G	-0.102	0.012	complement C8 gamma chain
PLEK	0.083	0.046	pleckstrin
IGLL1	0.080	0.050	immunoglobulin lambda like polypeptide 1
PGD	0.090	0.049	phosphogluconate dehydrogenase
CRISP3	0.095	0.024	cysteine rich secretory protein 3
GRIN2A	0.169	0.013	glutamate ionotropic receptor NMDA type subunit 2A
Q8NEJ1	0.092	0.023	uncharacterized protein
PGLYRP2	0.086	0.036	peptidoglycan recognition protein 2
CYP8B1	0.104	0.047	cytochrome P450 family 8 subfamily B member 1
FCGBP	0.104	0.050	Fc fragment of IgG binding protein
Regional BAG in precentral gyrus			
IGLV10-54	0.121	0.025	immunoglobulin lambda variable 10-54
IGHV2-70D	-0.085	0.043	immunoglobulin heavy variable 2-70D
IGKV2-24	0.110	0.046	immunoglobulin kappa variable 2-24
F12	0.084	0.040	coagulation factor XII
IGKV1-17	0.082	0.045	immunoglobulin kappa variable 1-17
IGLV1-44	-0.089	0.030	immunoglobulin lambda variable 1-44
IGLV3-19	0.081	0.047	immunoglobulin lambda variable 3-19
APOA2	0.102	0.012	apolipoprotein A2
ALB	-0.082	0.044	albumin
APOB	-0.090	0.027	apolipoprotein B
LCAT	-0.081	0.046	lecithin-cholesterol acyltransferase
HRG	0.126	0.002	histidine rich glycoprotein
IGLV7-43	-0.101	0.023	immunoglobulin lambda variable 7-43
IGF1	-0.089	0.033	insulin like growth factor 1
APOD	-0.083	0.041	apolipoprotein D
CLEC3B	-0.126	0.002	C-type lectin domain family 3 member B
IGKV2-30	0.111	0.013	immunoglobulin kappa variable 2-30
PLEK	0.092	0.026	pleckstrin
C4A,C4B	-0.086	0.034	Complement C4A, C4B
IGHV3-38-3	-0.093	0.022	immunoglobulin heavy variable 3-38-3
CPN1	-0.094	0.021	carboxypeptidase N subunit 1
IGLL1	0.082	0.044	immunoglobulin lambda like polypeptide 1
CD44	0.147	0.009	CD44 molecule (Indian blood group)
C4BPB	0.100	0.020	complement component 4 binding protein beta

GPX3	-0.094	0.021	glutathione peroxidase 3
IGFBP4	0.133	0.045	insulin like growth factor binding protein 4
BLVRB	-0.092	0.025	biliverdin reductase B
IGFALS	-0.103	0.011	insulin like growth factor binding protein acid labile subunit
KRT20	-0.103	0.018	keratin 20
NPEPPS	-0.135	0.011	aminopeptidase puromycin sensitive
RAP1B	-0.095	0.020	RAP1B, member of RAS oncogene family
ACTA1	-0.111	0.007	actin alpha 1, skeletal muscle
GRIN2A	0.139	0.042	glutamate ionotropic receptor NMDA type subunit 2A
Q5EBM2	0.084	0.041	uncharacterized protein
FRMPD3	-0.133	0.028	FERM and PDZ domain containing 3
Q6DHW4	0.087	0.036	uncharacterized protein
PI16	-0.113	0.005	peptidase inhibitor 16
ZSWIM9	0.095	0.020	zinc finger SWIM-type containing 9
C1RL	0.090	0.027	complement C1r subcomponent like
SERPINA10	-0.105	0.010	serpin family A member 10
Regional BAG in parahippocampal gyrus			
IGLV3-12	0.115	0.013	immunoglobulin lambda variable 3-12
IGLV5-45	0.088	0.038	immunoglobulin lambda variable 5-45
IGHV3-49	0.085	0.036	immunoglobulin heavy variable 3-49
CFB	0.080	0.048	complement factor B
IGLV1-40	0.090	0.028	immunoglobulin lambda variable 1-40
IGHG2	0.090	0.027	immunoglobulin heavy constant gamma 2 (G2m marker)
C1QA	0.089	0.028	complement C1q A chain
RBP4	0.112	0.006	retinol binding protein 4
VTN	0.086	0.034	vitronectin
ANXA1	0.086	0.037	annexin A1
APOB	-0.086	0.035	apolipoprotein B
IGLV7-43	-0.097	0.029	immunoglobulin lambda variable 7-43
A1BG	0.110	0.007	alpha-1-B glycoprotein
GAPDH	0.103	0.012	glyceraldehyde-3-phosphate dehydrogenase
CAPNS1	0.121	0.003	calpain small subunit 1
CFI	-0.109	0.008	complement factor I
VIM	0.081	0.049	vimentin
SERPINF2	0.087	0.032	serpin family F member 2
NME1	0.143	0.001	NME/NM23 nucleoside diphosphate kinase 1
PROZ	-0.088	0.040	protein Z, vitamin K dependent plasma glycoprotein
BLK	-0.114	0.022	BLK proto-oncogene, Src family tyrosine kinase
PGD	0.104	0.022	phosphogluconate dehydrogenase
CRISP3	0.098	0.020	cysteine rich secretory protein 3
LYZ	0.109	0.007	lysozyme
B2M	0.104	0.011	beta-2-microglobulin
RAN	0.105	0.022	RAN, member RAS oncogene family
TPM4	0.081	0.049	tropomyosin 4
CAP1	0.089	0.043	cyclase associated actin cytoskeleton regulatory protein 1
LGALS3BP	0.088	0.032	galectin 3 binding protein
ITIH4	0.096	0.018	inter-alpha-trypsin inhibitor heavy chain 4

PCOLCE	0.109	0.025	procollagen C-endopeptidase enhancer
C6orf132	-0.119	0.016	chromosome 6 open reading frame 132
DKFZp686C11235	0.111	0.010	uncharacterized protein
PRG4	0.082	0.045	proteoglycan 4
PGLYRP2	0.081	0.046	peptidoglycan recognition protein 2
GLIPR2	0.098	0.021	GLI pathogenesis related 2
Regional BAG in cerebellum I-IV			
IGKV1D-8	-0.095	0.021	immunoglobulin kappa variable 1D-8
IGHV1-3	-0.110	0.007	immunoglobulin heavy variable 1-3
ATP8B3	0.315	0.004	ATPase phospholipid transporting 8B3
CST3	0.084	0.040	cystatin C
IGLV2-23	-0.106	0.009	immunoglobulin lambda variable 2-23
IGLV2-8	-0.088	0.030	immunoglobulin lambda variable 2-8
IGLV3-27	-0.086	0.035	immunoglobulin lambda variable 3-27
APOA2	0.086	0.036	apolipoprotein A2
AMBP	0.081	0.048	alpha-1-microglobulin/bikunin precursor
TF	0.098	0.016	transferrin
IGLV7-43	-0.093	0.035	immunoglobulin lambda variable 7-43
CLEC3B	-0.092	0.025	C-type lectin domain family 3 member B
SAA2	0.150	0.001	serum amyloid A2
PZP	0.112	0.017	PZP alpha-2-macroglobulin like
ARHGDIB	0.159	0.000	Rho GDP dissociation inhibitor beta
INHBC	0.128	0.010	inhibin subunit beta C
ANTXR2	-0.102	0.039	ANTXR cell adhesion molecule 2
B2M	0.093	0.023	beta-2-microglobulin
ITIH3	-0.080	0.049	inter-alpha-trypsin inhibitor heavy chain 3
LGALS3BP	0.093	0.023	galectin 3 binding protein
Q569I7	0.104	0.010	uncharacterized protein
SBSN	-0.232	0.035	suprabasin
PUM2	0.127	0.040	pumilio RNA binding family member 2
SH3BGRL3	0.096	0.019	SH3 domain binding glutamate rich protein like 3

Partial spearman correlation analysis adjusted age, sex and TIV

Table S8. List of proteins with significant difference between accelerated aging and delayed aging groups. Related to Figure 5.

Protein	t-value	p-value	Protein description
Delay aging group > Accelerated aging group			
IGLV4_60	3.063	0.003	immunoglobulin lambda variable 4-60
IGHV4_4	2.262	0.027	immunoglobulin heavy variable 4-4
IGLC7	2.137	0.037	immunoglobulin lambda constant 7
IGKV2_29	2.031	0.046	immunoglobulin kappa variable 2-29
SDPR	2.802	0.011	serum deprivation-response protein
HP	2.312	0.024	haptoglobin
IGKV3_20	2.141	0.036	immunoglobulin kappa variable 3-20
IGLV3_19	2.394	0.019	immunoglobulin lambda variable 3-19
SAA2	2.118	0.038	serum amyloid A2
PTGFR	2.029	0.047	prostaglandin F receptor
CRISP3	2.106	0.039	cysteine rich secretory protein 3
TPM4	2.022	0.047	tropomyosin 4
SMCO1	2.279	0.027	single-pass membrane protein with coiled-coil domains 1
Q6DHW4	2.021	0.047	uncharacterized protein
Q6PJG0	2.539	0.014	uncharacterized protein
Q7Z2U7	2.513	0.014	uncharacterized protein
ZSWIM9	2.322	0.023	zinc finger SWIM-type containing 9
ABCC2	2.158	0.035	ATP binding cassette subfamily C member 2
TAS2R4	2.052	0.045	taste 2 receptor member 4
Delay aging group < Accelerated aging group			
IGHV1_69D	-3.526	0.001	immunoglobulin heavy variable 1-69D
IGHV1_69_2	-2.077	0.042	immunoglobulin heavy variable 1-69-2
C4BPA	-2.176	0.033	complement component 4 binding protein alpha
VCAM1	-2.318	0.024	vascular cell adhesion molecule 1
KRT20	-3.341	0.001	keratin 20
TSC2	-2.216	0.034	TSC complex subunit 2

ANCOVA model adjusted baseline age & sex

Table S9. Coverage analysis of the MS-based proteomic panel compared with reference plasma proteome. Related to Discussion

Location/Protein type	Current study	HPA reference	FDR
Part A. Subcellular location distribution.			
Extracellular Space	209 (42.6%)	727 (17.0%)	5.50E-35
Cytoplasm	108 (22.0%)	2035 (47.5%)	2.00E-28
Nucleus	27 (5.5%)	585 (13.7%)	3.70E-08
Plasma Membrane	56 (11.4%)	815 (19.0%)	1.90E-05
Other	92 (18.7%)	123 (2.9%)	9.00E-37
Part B. Protein type distribution.			
1 enzyme	74 (15.1%)	1104 (25.8%)	4.60E-07
2 translation regulator	0 (0%)	64 (1.5%)	0.01
3 kinase	9 (1.8%)	165 (3.9%)	0.07
4 transcription regulator	8 (1.6%)	144 (3.4%)	0.09
5 transporter	39 (7.9%)	239 (5.6%)	0.09
6 phosphatase	4 (0.8%)	78 (1.8%)	0.26
7 peptidase	35 (7.1%)	250 (5.8%)	0.42
8 transmembrane receptor	16 (3.3%)	187 (4.4%)	0.42
9 ion channel	4 (0.8%)	27 (0.6%)	0.72
10 growth factor	6 (1.2%)	61 (1.4%)	0.99
11 cytokine	5 (1.0%)	46 (1.1%)	1
12 G-protein coupled receptor	2 (0.4%)	20 (0.5%)	1
13 other	289 (58.9%)	1900 (44.3%)	1.40E-08

Current study includes 492 proteins mapped to IPA categories.

HPA reference includes 4,285 plasma proteins detected by MS from the Human Protein Atlas.

FDR values were computed using Fisher's exact tests followed by Benjamini–Hochberg correction.

Table S10. Comparison of baseline characteristics between return and non-return participants. Related to STAR Methods.

	**Return subset** [Participants in longitudinal analysis] **(n=444)**	**Non-return subset** [Attrition participants] **(n=796)**	**p-value**	**Overall (n=1,240)**
Demographic				
Age, years	59.2 ± 6.3	65.2 ± 9.3	<0.001	63.1 ± 8.8
Female, n (%)	238 (53.6%)	418 (52.5%)	0.712	656 (52.9%)
Education, years	9.1 ± 4.5	5.6 ± 4.8	<0.001	6.8 ± 5.0
Lifestyle and Risk factors				
Smoking, n (%)	53 (11.9%)	157 (19.7%)	<0.001	210 (16.9%)
Alcohol drinking, n (%)	170 (38.3%)	272 (34.2%)	0.147	442 (35.6%)
Hypertension, n (%)	134 (30.2%)	344 (43.2%)	<0.001	478 (38.5%)
Diabetes mellitus, n (%)	46 (10.4%)	142 (17.8%)	<0.001	188 (15.2%)
Neuroimaging measurement				
Total intracranial volume, mL	1339 ± 120	1334 ± 117	0.791[a]	1336 ± 118
Global brain age gap, years	-0.60 ± 9.34	1.99 ± 9.20	0.004[b]	1.06 ± 9.33
Physical examination				
Handgrip, kg	30.7 ± 9.9	28.0 ± 9.2	0.349[a]	29.0 ± 9.5
Gait speed, m/s	1.7 ± 0.5	1.5 ± 0.4	<0.001[a]	1.6 ±0.5
Cognitive performance, scores				
Mini-Mental State Examination	27.5 ± 2.5	25.5 ± 4.0	0.393[c]	26.2 ± 3.6

[a]ANCOVA test, adjusted for age and sex.

[b]ANCOVA test, adjusted for age, sex and total intracranial volume.

[c]ANCOVA test, adjusted for age, sex and education years.

Table S11. Sensitivity analysis about the effect of MRI upgrading in BAG change. Related to STAR Methods.

	**No upgrade**<br>TrioTim	**Upgrade**<br>TrioTim to PrismaFit	**p-value**
N	253	191	
Baseline age, years	59.0 ± 6.2	59.4 ± 6.4	0.486
Female, n (%)	138 (54.5%)	100 (52.4%)	0.701
Baseline TIV, mL	1338 ± 115	1340 ± 126	0.951a
Follow-up TIV, mL	1338 ± 117	1395 ± 139	<0.001b
Baseline BAG, years	-0.60 ± 9.65	-0.60 ± 8.94	0.946c
Follow-up BAG, years	1.29 ± 9.57	3.06 ± 8.57	0.026d
Annual BAG change (ΔBAG), years	0.27 ± 0.54	0.42 ± 0.58	0.007a

[a]ANCOVA test, adjusted for baseline age and sex.

[b]ANCOVA test, adjusted for follow-up age and sex.

[c]ANCOVA test, adjusted for baseline age, sex and baseline total intracranial volume.

[d]ANCOVA test, adjusted for follow-up age, sex and follow-up total intracranial volume.

Table S12. Comparison of baseline characteristics between participants with and without proteomic data. Related to STAR Methods.

	Proteomic subset (n=606)	Non-proteomic subset (n=634)	p-value	Overall (n=1,240)
Demographic				
Age, years	62.7 ± 8.6	63.4 ± 9.0	0.175	63.1 ± 8.8
Female, n (%)	327 (54.0%)	329 (51.9%)	0.466	656 (52.9%)
Education, years	7.2 ± 5.0	6.5 ± 5.0	0.012	6.8 ± 5.0
Lifestyle and Risk factors				
Smoking, n (%)	94 (15.5%)	116 (18.3%)	0.191	210 (16.9%)
Alcohol drinking, n (%)	210 (34.7%)	232 (36.6%)	0.476	442 (35.6%)
Hypertension, n (%)	220 (36.6%)	258 (40.7%)	0.112	478 (38.5%)
Diabetes mellitus, n (%)	93 (15.3%)	95 (15.0%)	0.859	188 (15.2%)
Neuroimaging measurement				
Total intracranial volume, mL	1336 ± 117	1336 ± 119	0.633[a]	1336 ± 118
Global brain age gap, years	0.63 ± 9.23	1.48 ± 9.42	0.174[b]	1.06 ± 9.33
Physical examination				
Handgrip, kg	29.2 ± 9.4	28.8 ± 9.6	0.141[a]	29.0 ± 9.5
Gait speed, m/s	1.7 ± 0.5	1.5 ± 0.4	<0.001[a]	1.6 ±0.5
Cognitive performance, scores				
Mini-Mental State Examination	26.4 ± 3.2	26.0 ± 4.0	0.468[c]	26.2 ± 3.6

[a]ANCOVA test, adjusted for age and sex.

[b]ANCOVA test, adjusted for age, sex and total intracranial volume.

[c]ANCOVA test, adjusted for age, sex and education years.

Table S14. Data sources for brain age training cohort. Related to STAR Methods.

Cohort	N	Age mean±SD [range]	Sex male/female	Scanner	Coil	Sequence	Protocol parameters
National Yang Ming Chiao Tung University (NYCU)	808	46.2±20.4 [18-92]	407/401	Siemens Tim Trio (3T)	12-channel / 32-channel phased-array head coil	MPRAGE	TR/ TE/ TI = 3500/3.5/ 1100 msec; flip angle = 7°; NEX = 1; FOV = 256 x 256 mm^2; matrix size = 256 x 256; 192 sagittal slices; and voxel size = 1.0 x 1.0 x 1.0 mm^3
Taipei Veteran General Hospital (TVGH)	293	54.4±17.8 [22-92]	94/199	GE Discovery MR750 (3T)	8-channel phased-array head coil	IR-FSPGR	TR/TE/TI = 9.4/4.0/450 msec; flip angle = 12°; NEX = 1; FOV = 256 x 256 mm^2; matrix size = 256 x 256; 172 axial slices; and voxel size = 1.0 x 1.0 x 1.0 mm^3
						BRAVO	TR/TE/TI = 9.2/3.7/450 msec; flip angle = 12°; NEX = 1; FOV = 256 x 256 mm^2; matrix size = 256 x 256; 168 axial slices; and voxel size = 1.0 x 1.0 x 1.0 mm^3
Tri-Service General Hospital (TSGH)	68	42.0±11.1 [21-62]	21/47	GE Discovery MR750 (3T)	8-channel phased-array head coil	BRAVO	TR/TE/TI = 10.17/4.16/450 msec; flip angle = 12°; NEX = 1; FOV = 256 × 256 mm^2; matrix size = 256 × 256; 172 axial slices; and voxel size = 1.0 x 1.0 x 1.0 mm^3
Kaohsiung Chang Gung Memorial Hospital (KHCGMH)	244	48.5±14.3 [20-75]	127/117	Siemens Skyra (3T)	20-channel phased-array head/neck coil	MPRAGE	TR/ TE/TI = 1800/2.7/900 msec; flip angle = 9°; NEX = 1; FOV = 230 x 230 mm^2; matrix size = 192 x 192; 144 sagittal slices; and voxel size = 1.2 x 1.2 x 1.2 mm^3
				GE Signa Excite (3T)	8-channel phased-array head coil	IR-FSPGR	TR/ TE/ TI = 9.5/3.9/450 msec; flip angle = 15°; NEX = 1; FOV = 240 × 240 mm^2; matrix size = 512 × 512; 110 axial slices; and voxel size = 0.47 × 0.47 × 1.3 mm^3
Keelung Chang Gung Memorial Hospital (KLCGMH)	69	65.0±5.1 [52-77]	32/37	Siemens Skyra (3T)	20-channel phased-array head/neck coil	MPRAGE	TR/TE/TI = 2,200/2.45/900 msec; flip angle = 8°, NEX=1; FOV = 256 x 256 mm^2; matrix size = 256 x 256; 176 sagittal slices; and voxel size = 1.0 x 1.0 x 1.0 mm^3

Abbreviations: TR, Repetition time; TE, echo time; TI, inversion time; NEX, number of excitations; FOV, field of view;
