# Supplementary material for: Regional brain aging signatures predict mortality through neurovascular immune mechanisms
Source: Cell Rep Med. 2026 Oct 7;7(10):103096. doi: 10.1016/j.xcrm.2026.103096 (PMC13710373; doi:10.1016/j.xcrm.2026.103096)
Supplement: Supplementary file 2 [file mmc6.pdf]

Cell Reports Medicine

Article

Regional brain aging signatures predict mortality through neurovascular immune mechanisms

Graphical abstract

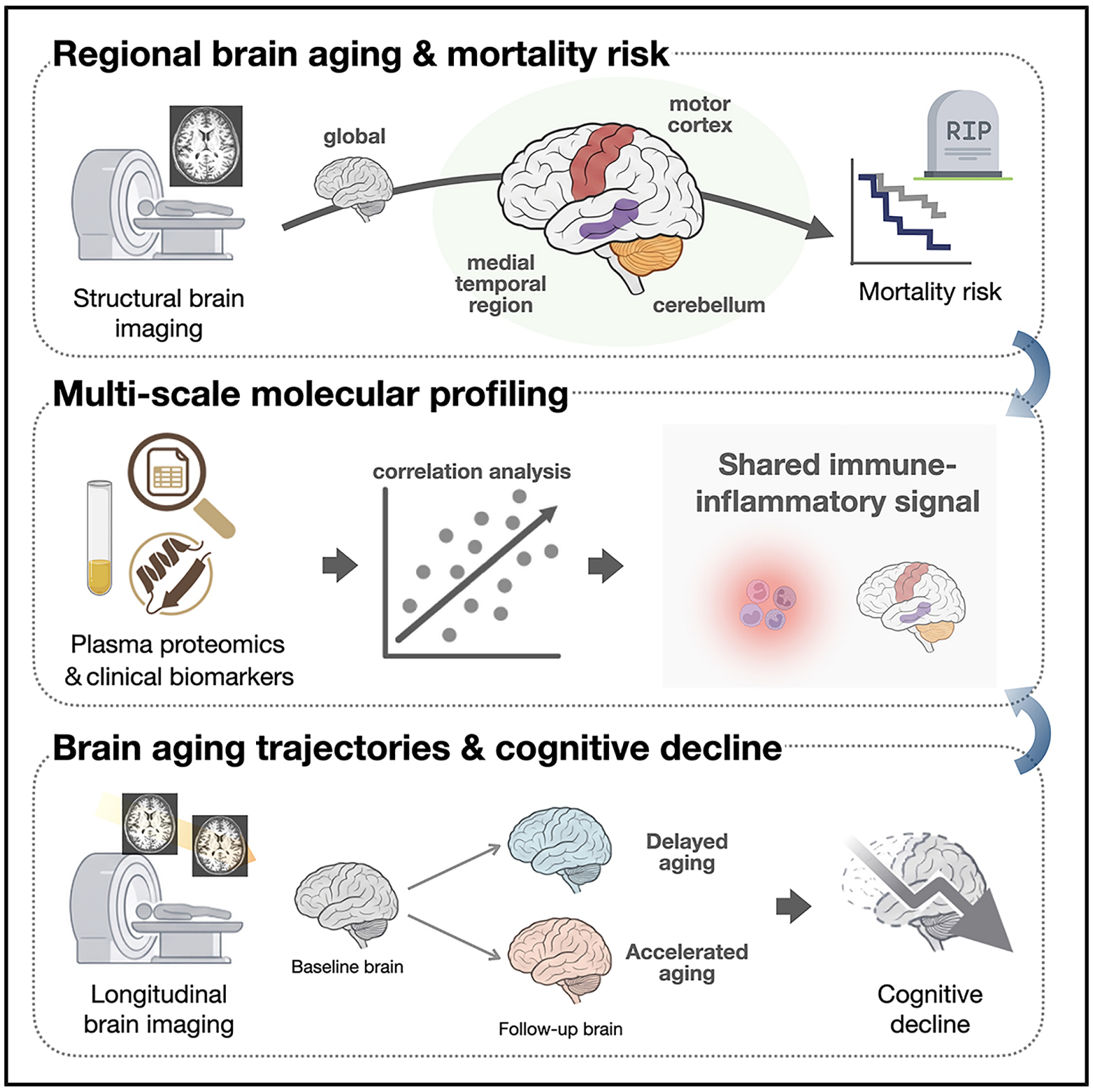

Highlights

- Global brain age gap independently predicts 10-year mortality risk
- A three-region brain age model outperforms global measures for mortality prediction
- Multi-scale integration links regional brain aging to immune-inflammatory pathways
- Accelerated brain aging predicts cognitive decline in longitudinal follow-up

Authors

Pei-Lin Lee, Yi-Long Huang, Chih-Kuang Liang, ..., Fei-Yuan Hsiao, Ching-Po Lin, Liang-Kung Chen

Correspondence

lkchen2@vghtpe.gov.tw

In brief

Lee et al. integrate structural brain MRI with plasma proteomics to identify a three-region brain age model that predicts mortality more accurately than global measures and reveals shared immune and vascular mechanisms. These findings support regional brain aging as a scalable biomarker for precision geromedicine.

Lee et al., 2026, Cell Reports Medicine 7, 103096
October 20, 2026 © 2026 The Author(s). Published by Elsevier Inc.
https://doi.org/10.1016/j.xcrm.2026.103096

CellPress

Cell Reports Medicine

CellPress
OPEN ACCESS

Article

Regional brain aging signatures predict mortality through neurovascular immune mechanisms

Pei-Lin Lee,[1] Yi-Long Huang,[1] Chih-Kuang Liang,[2,3,4] Wei-Ju Lee,[1,5] Li-Ning Peng,[1,6] Fei-Yuan Hsiao,[7,8,9] Ching-Po Lin,[10,11,12] and Liang-Kung Chen[1,6,13,14,*]

[1]Center for Healthy Longevity and Aging Sciences, National Yang Ming Chiao Tung University, Taipei 112304, Taiwan
[2]Center for Geriatrics and Gerontology, Kaohsiung Veterans General Hospital, Kaohsiung 813414, Taiwan
[3]Division of Neurology, Department of Internal Medicine, Kaohsiung Veterans General Hospital, Kaohsiung 813414, Taiwan
[4]Department of Geriatric Medicine, School of Medicine, National Yang Ming Chiao Tung University, Taipei 112304, Taiwan
[5]Department of Family Medicine, Taipei Veterans General Hospital Yuanshan Branch, Yilan 26444, Taiwan
[6]Center for Geriatrics and Gerontology, Taipei Veterans General Hospital, Taipei 11217, Taiwan
[7]Graduate Institute of Clinical Pharmacy, College of Medicine, National Taiwan University, Taipei 10051, Taiwan
[8]School of Pharmacy, College of Medicine, National Taiwan University, Taipei 10051, Taiwan
[9]Department of Pharmacy, National Taiwan University Hospital, Taipei 100225, Taiwan
[10]Institute of Neuroscience, National Yang Ming Chiao Tung University, Taipei 112304, Taiwan
[11]Brain Research Center, National Yang Ming Chiao Tung University, Taipei 112304, Taiwan
[12]Department of Education and Research, Taipei City Hospital, Taipei 103212, Taiwan
[13]Taipei Municipal Gan-Dau Hospital (managed by Taipei Veterans General Hospital), Taipei 112020, Taiwan
[14]Lead contact
*Correspondence: lkchen2@vghtpe.gov.tw
https://doi.org/10.1016/j.xcrm.2026.103096

SUMMARY

Brain aging provides an integrative marker of systemic health, but whether regional patterns outperform whole-brain approaches remains unknown. Here, we show that regional brain age signatures achieve superior mortality prediction through identifiable neurovascular-immune mechanisms in 1,240 community-dwelling adults followed for 10 years (120 deaths). While global brain age independently predicts mortality (hazard ratio [HR], 1.044 per year), a data-driven three-region model combining the left precentral gyrus, left parahippocampal gyrus, and right cerebellum achieves superior discrimination (C-index, 0.81 vs. 0.79, $p = 0.032$). Integration with plasma proteomics (513 proteins) reveals complement activation and adaptive immune dysregulation as convergent mechanisms. Region-specific vulnerabilities emerge: microvascular dysfunction in the motor cortex, proteostasis failure in the medial temporal structures, and iron dysregulation in the cerebellum. Longitudinal imaging ($n = 444$) identifies accelerated brain aging phenotypes predicting cognitive decline (HR, 1.558; $p = 0.042$). These findings establish regional neuroimaging-proteomic integration as a precision strategy linking network-specific vulnerabilities to systemic immune processes.

INTRODUCTION

Individuals of identical chronological age exhibit marked heterogeneity in mortality risk, with differences in accumulated health deficits accounting for much of this variation.[1] Biological aging processes, rather than chronological time, thus govern individual vulnerability to age-related conditions, including cardiovascular disease, dementia, and frailty.[2,3] Among various organ systems, the brain represents a particularly informative target for biological age assessment. As a metabolically demanding tissue, the brain exhibits exquisite vulnerability to systemic perturbations—including vascular dysfunction, chronic inflammation, and metabolic dysregulation—that accumulate during biological aging.[4] These multi-system insults manifest as progressive structural changes, including tissue atrophy, synaptic loss, and network reorganization, detectable via neuroimaging years before clinical symptom onset.[4] Despite these insights, clinical risk stratification continues to rely predominantly on chronological age, which limits precision in identifying high-risk individuals and targeting preventive interventions with precision, particularly for brain-related age-associated conditions. Recent advances in structural magnetic resonance imaging (MRI), combined with machine learning, have enabled the quantification of brain biological age from neuroimaging features, providing a data-driven measure of brain health.[5,6] The brain age gap (BAG)—the difference between predicted and chronological brain age—quantifies deviation from normative aging, providing an integrative biomarker of biological aging independent of chronological time.

Accumulating evidence demonstrated that BAG predicted mortality risk across diverse populations, with positive values indicating elevated risk.[7–9] Beyond mortality, elevated BAG predicted both progression to Alzheimer's disease dementia and accelerated cognitive decline.[10,11] Importantly, brain age not merely reflects localized brain pathology but serves as an

This is an open access article under the CC BY license (http://creativecommons.org/licenses/by/4.0/).

integrative measure of systemic biological aging.[12] Evidence for this systemic integration included associations with physical frailty indicators—impaired gait speed, reduced grip strength, and compromised pulmonary function[9]—and cardiometabolic disorders such as hypertension, diabetes mellitus, and cardiovascular disease.[7] Brain age demonstrated responsiveness to modifiable lifestyle factors, with smoking cessation, regular physical activity, and moderate alcohol consumption each associated with younger brain-predicted age.[8,12] Implementation of brain age estimation in large-scale population cohorts has established its utility as a biomarker for clinical risk stratification and as a potential therapeutic target for interventions promoting healthy brain aging.[6]

Despite these advances, three critical gaps limit mechanistic understanding and precision medicine applications. First, brain age research has predominantly utilized whole-brain summary measures, potentially obscuring regional variations that may improve mortality risk prediction. Brain aging proceeds heterogeneously across regions, with distinct neuroanatomical substrates exhibiting differential vulnerability to age-related pathology.[11,13] Whole-brain approaches may thus overlook regional patterns containing the most robust predictive information for mortality outcomes. Regional brain age investigations have remained limited in scope and have primarily examined non-mortality outcomes; no comprehensive analysis has yet characterized how regional brain aging patterns relate to mortality risk across the full brain. Second, the mechanisms linking accelerated brain aging to mortality remain incompletely understood. The systemic processes connecting regional brain atrophy to death risk, and the pathways mediating brain-periphery communication during aging, require elucidation for rational intervention design. Blood constitutes a critical mediator, with circulating protein profiles potentially reflecting brain-periphery crosstalk.[14,15] However, most investigations have employed targeted biomarker panels rather than unbiased proteomic profiling, with limited integration of neuroimaging-derived brain age measures and multi-domain assessments spanning inflammatory, metabolic, cardiovascular, and proteomic systems. Third, cross-sectional BAGs pose interpretational challenges, potentially reflecting historical insults or developmental variations rather than ongoing accelerated aging.[16] Longitudinal measurements are essential to establish whether elevated BAGs represent dynamic aging trajectories conferring mortality risk. Yet, longitudinal brain age studies have remained scarce, typically involving brief follow-up intervals and lacking comprehensive phenotyping of individuals exhibiting accelerated aging trajectories.

To address these gaps, we performed a comprehensive multiscale analysis in the I-Lan Longitudinal Aging Study (ILAS), a prospective community-based cohort in Taiwan. To derive unbiased individual brain age estimates, we developed prediction models using a large external training dataset, generating both global and regional brain age estimates across 442 brain regions encompassing cortical, subcortical, and cerebellar structures. We evaluated whether regional BAGs provided superior mortality prediction compared to whole-brain approaches and identified the optimal regional combination for mortality risk stratification. We further investigated the molecular mechanisms linking brain aging to mortality by integrating neuroimaging-derived brain age measures with comprehensive biological profiling, including circulating biomarkers spanning metabolic, inflammatory, and cardiovascular domains; physical performance assessments; cognitive performance; and untargeted plasma proteomics. In a longitudinal subset with serial neuroimaging, we characterized distinct brain aging phenotypes and examined their associations with cognitive decline trajectories and underlying proteomic signatures. We hypothesized that regional brain age patterns would exhibit heterogeneous associations with mortality risk, that systemic inflammation would emerge as a unifying molecular mechanism across brain regions, and that longitudinally defined accelerated brain aging would predict subsequent cognitive decline.

RESULTS

Global brain age gap independently predicts 10-year mortality

The analysis included 1,240 participants with complete baseline data and mortality follow-up (age 63.1 ± 8.8 years, 52.9% female, 120 deaths over a median 8.75-year follow-up, Table 1). The detailed baseline characteristics stratified by mortality status are presented in Table S1. Global BAG was significantly elevated in participants who died during follow-up compared to survivors (5.11 ± 8.89 vs. 0.63 ± 9.28 years, mean difference +4.48 years, $p < 0.001$ after adjusting for age, sex, and total intracranial volume [TIV]; Figure 1A). In sex-stratified analyses, the BAG elevation was statistically significant in males (mean difference +5.17 years, $p < 0.001$) and showed a similar direction but did not reach significance in females (+3.90 years, $p = 0.094$), likely reflecting the smaller number of deaths in females.

Cox proportional hazards regression analysis established the global BAG as an independent predictor of mortality risk. In the minimally adjusted model controlling for age, sex, and TIV, each additional year of BAG was associated with a 4.4% increased hazard of death (hazard ratio [HR] = 1.044, 95% confidence interval [CI]: 1.022–1.067, $p < 0.001$; Table 2). The association remained statistically significant following comprehensive adjustment for potential confounders, including education, smoking status, alcohol consumption, hypertension, and diabetes mellitus (HR = 1.037, 95% CI: 1.015–1.059, $p = 0.001$; Table 2). To formally test whether sex modified this association, we further included a BAG × sex interaction term in the fully adjusted Cox regression model. The interaction term was not statistically significant (HR = 0.988, 95% CI: 0.944–1.034, $p = 0.610$), indicating that sex did not significantly modify the BAG-mortality association after adjustment for demographic, lifestyle, and cardiovascular risk factors. Kaplan-Meier survival curves stratified by global BAG tertiles provided visual confirmation of these dose-response relationships (Figure 1B), demonstrating progressive separation across strata (log rank test $p < 0.001$). Global BAG showed significant associations with established cardiovascular risk factors (Figure 1C). Participants with hypertension had higher BAG compared to those without (mean difference +3.2 years, $p < 0.001$). Similarly, participants with diabetes mellitus showed elevated BAG (+3.5 years, $p < 0.001$), as did current or former smokers compared to never-smokers

CellPress
OPEN ACCESS

Table 1. Baseline characteristics of study samples

	Cross-sectional samples (*n* = 1,240)			Longitudinal samples (*n* = 444)		
	Whole sample	Male	Female	Whole sample	Male	Female
Demographic						
n	1,240	584	656	444	206	238
Age, years	63.1 ± 8.8	64.2 ± 9.4	62.1 ± 8.1	59.2 ± 6.3	59.8 ± 6.6	58.6 ± 4.6
Education, years	6.8 ± 5.0	7.8 ± 5.1	6.0 ± 4.8	9.1 ± 4.5	9.9 ± 4.3	8.4 ± 4.6
Lifestyle and risk factors						
Smoking, *n* (%)	210 (16.9%)	194 (33.2%)	16 (2.4%)	53 (11.9%)	51 (24.8%)	2 (0.8%)
Alcohol drinking, *n* (%)	442 (35.6%)	312 (53.4%)	130 (19.8%)	170 (38.3%)	124 (60.2%)	46 (19.3%)
Hypertension, *n* (%)	478 (38.5%)	228 (39.0%)	250 (38.1%)	134 (30.2%)	64 (31.1%)	70 (29.4%)
Diabetes mellitus, *n* (%)	188 (15.2%)	92 (15.8%)	96 (14.6%)	46 (10.4%)	25 (12.1%)	21 (8.8%)
Physical examination						
Handgrip, kg	29.0 ± 9.5	36.2 ± 8.0	22.6 ± 5.2	30.7 ± 9.9	39.3 ± 6.8	23.3 ± 5.0
Gait speed, m/s	1.6 ± 0.5	1.7 ± 0.5	1.5 ± 0.4	1.7 ± 0.5	1.8 ± 0.5	1.6 ± 0.4
Cognitive performance, scores						
Mini-Mental State Examination	26.2 ± 3.6	26.7 ± 3.2	25.7 ± 3.9	27.5 ± 2.5	28.0 ± 1.9	27.1 ± 3.0
10-min CVVLT	6.6 ± 2.1	6.4 ± 2.1	6.8 ± 2.0	7.1 ± 1.7	7.0 ± 1.8	7.3 ± 1.6
Clock drawing test	7.7 ± 2.5	8.0 ± 2.3	7.4 ± 2.7	8.6 ± 1.9	8.8 ± 1.6	8.4 ± 2.1
Taylor Complex Figure test	30.6 ± 7.1	31.4 ± 6.0	29.8 ± 7.8	32.8 ± 4.8	33.4 ± 3.7	32.4 ± 5.6
Boston Naming test	12.4 ± 2.5	13.0 ± 2.3	11.9 ± 2.6	13.3 ± 2.0	13.8 ± 1.6	12.8 ± 2.2
Backward Digit test	3.6 ± 2.0	3.9 ± 1.8	3.4 ± 2.0	4.3 ± 1.7	4.6 ± 1.5	4.1 ± 1.8
Neuroimaging measurement, mL						
Total intracranial volume	1,336 ± 118	1,414 ± 97	1,266 ± 88	1,339 ± 120	1,419 ± 99	1,270 ± 90
Gray-matter volume	629 ± 61	654 ± 58	606 ± 53	645 ± 54	671 ± 50	622 ± 48
White-matter volume	416 ± 50	439 ± 48	395 ± 42	425 ± 48	450 ± 46	404 ± 39
Cerebrospinal fluid volume	292 ± 73	321 ± 70	265 ± 64	269 ± 64	298 ± 63	245 ± 55
Death, *n* (%)	120 (9.7%)	78 (13.4%)	42 (6.4%)	–	–	–
Follow-up periods of mortality tracing, years	8.66 ± 1.30	8.64 ± 1.44	8.67 ± 1.16	–	–	–
Follow-up intervals of MRI scans, years	–	–	–	7.8 ± 1.3	7.9 ± 1.2	7.8 ± 1.3

CVVLT, Chinese version Verbal Learning Test.

(+1.3 years, $p = 0.004$). No significant association was observed between BAG and alcohol consumption ($p = 0.154$).

Three-region model achieves superior mortality discrimination

Univariate Cox regression analysis of regional BAGs across 442 brain regions identified 44 regions significantly associated with mortality after Bonferroni correction ($p < 1.13 \times 10^{-4}$; Figure 2A; Table S2). For anatomical interpretation, cortical regions were mapped to Harvard-Oxford anatomical labels. Network distribution showed that sensorimotor network regions comprised 34% of significant areas (15 regions), followed by default mode network (14%, 6 regions), subcortical structures (11%, 5 regions), and visual networks (11%, 5 regions; Figure 2B). Subcortical structures demonstrated the strongest associations, with the right and left thalamus showing the highest hazard ratios (HR = 1.050, 95% CI: 1.025–1.075, $p < 0.001$; HR = 1.049, 95% CI: 1.024–1.074, $p < 0.001$, respectively; Figure 2C). All 44 regions remained significant ($p < 0.05$) after full adjustment for demographics, lifestyle factors, and cardiovascular risk factors (Table S3). Forward stepwise selection identified an optimal three-region model (Figure 3A; Table 2). The selected regions represent distinct functional systems: left precentral gyrus (sensorimotor network), left parahippocampal gyrus anterior division (limbic network), and right cerebellum I–IV (cerebellar system). Inter-correlations among the global and the three regional BAGs were modest (Table S4), supporting their capture of partially independent brain aging information. Multivariate Cox regression demonstrated independent contributions from each region: left precentral gyrus (HR = 1.016, 95% CI: 1.005–1.026, $p = 0.003$), left parahippocampal gyrus (HR = 1.019, 95% CI: 1.008–1.031, $p < 0.001$), and right cerebellum I–IV (HR = 1.022, 95% CI: 1.007–1.037, $p = 0.004$). Without any covariate adjustment, the three regional BAGs jointly yielded a C-index of 0.73 (95% CI: 0.68–0.77), while the global BAG alone yielded 0.62 (95% CI: 0.57–0.67), both exceeding chance-level discrimination. Model performance comparison showed progressive improvement (Figure 3B). C-index values ranged from 0.75 (baseline demographic model) to 0.81 (three-region model with expanded covariates). The three-region model showed superior discrimination over the global BAG (C-index: 0.80 vs. 0.77, bootstrap

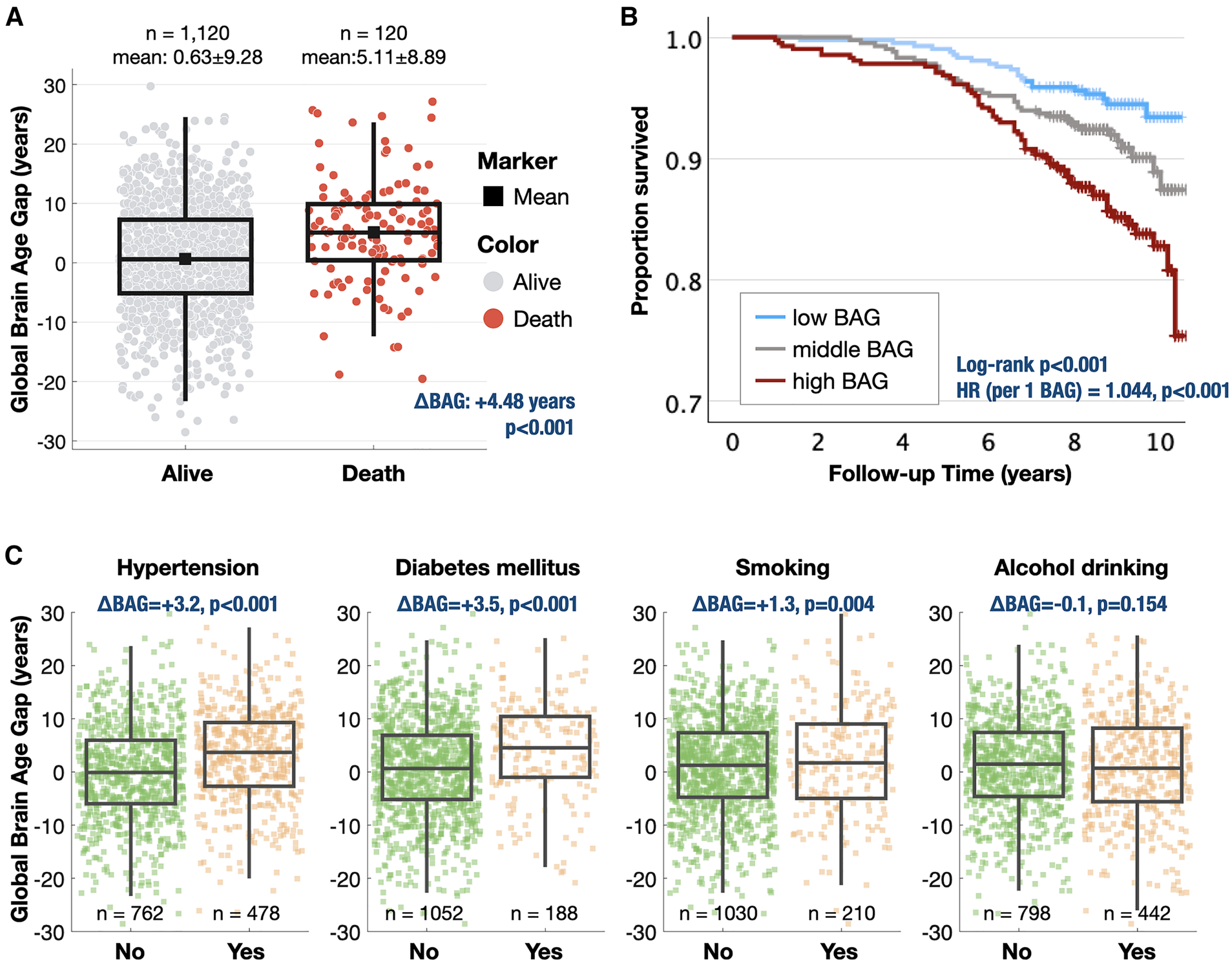

Figure 1. Global brain age gap associates with all-cause mortality risk

(A) Global BAG distribution by survival status (alive n = 1,120, died n = 120). Boxplots show median and interquartile range; dots represent individual participants. Deceased individuals showed significantly elevated BAG ($p < 0.001$, ANCOVA adjusted for age, sex, and TIV).

(B) Kaplan-Meier survival curves stratified by global BAG tertiles: low BAG (blue line), middle BAG (gray line), and high BAG (red line). Censoring events indicated by cross marks. The log-rank p value compares survival across tertiles; HR indicates the hazard ratio per 1-year increase in global BAG from Cox regression model.

(C) Global BAG across cardiovascular risk factor groups. Boxplots compare BAG between individuals with (orange) and without (green) hypertension, diabetes, smoking, and alcohol use. The group comparison analyses were adjusted for age, sex, and TIV.

BAG, brain age gap; TIV, total intracranial volume; ΔBAG, difference in mean BAG between groups.

p = 0.034 with basic demographics; 0.81 vs. 0.79, bootstrap p = 0.032 with expanded covariates).

Multi-scale integration reveals convergent inflammatory mechanisms

To investigate the biological mechanisms underlying regional brain aging patterns, we examined the associations between the global BAG and three regional BAGs with 46 plasma biomarkers, 3 physical function assessments, and 6 cognitive test scores (Figure 4A; Table S5). A convergent inflammatory signature emerged as the primary shared mechanism across all brain age measures. Homocysteine and neutrophil count showed consistent positive correlations with the global BAG and all three regional BAGs. Hematologic parameters, including white blood cell count and platelet count, demonstrated similar cross-regional associations. Beyond these shared patterns, region-specific biomarker associations were observed across the hepatic, renal, and endocrine domains. Cognitive performance measures demonstrated negative correlations with BAGs, with the language, visual-spatial, and executive function domains showing the strongest and most consistent associations across regions. Physical function measures (grip strength and gait speed) showed similar negative correlations across all brain regions.

Proteomic analysis revealed 19–40 region-associated proteins per brain region (Figure 4B; Table S6; Figure S1A). Global BAG showed associations with proteins including APOD, APOB, and GRIN2A. Region-specific proteomic signatures included hemostatic and extracellular matrix-associated proteins for the precentral gyrus (HRG and CD44), metabolic and proteolytic markers associated with neurodegeneration for the parahippocampal gyrus (NME1 and CAPNS1), and inflammatory and membrane-integrity-related proteins for the cerebellum (SAA2 and ATP8B3). These differential protein signatures suggest distinct molecular pathways underlying regional brain aging vulnerabilities. Pathway enrichment analysis of brain age-associated proteins revealed convergent immune response activation across all regions, alongside region-specific signatures

Table 2. Cox proportional hazards regression models for all-cause mortality risk

Variables	Model 1 Global BAG (minimal adjustment)			Model 2 Global BAG (full adjustment)			Model 3 Optimal regional BAGs (minimal adjustment)			Model 4 Optimal regional BAGs (full adjustment)		
–	HR	95% CI	*p* value	HR	95% CI	*p* value	HR	95% CI	*p* value	HR	95% CI	*p* value
Age, per year	1.096	1.075–1.118	<0.001	1.084	1.059–1.110	<0.001	1.079	1.057–1.102	<0.001	1.071	1.045–1.097	<0.001
Female (vs. male)	0.733	0.454–1.183	0.204	0.806	0.470–1.384	0.435	0.722	0.447–1.167	0.183	0.743	0.431–1.281	0.285
TIV, per 100 mL	1.151	0.946–1.400	0.161	1.156	0.950–1.407	0.148	1.293	1.064–1.570	0.010	1.251	1.032–1.517	0.023
Education, per year	–	–	–	0.983	0.937–1.031	0.486	–	–	–	0.987	0.940–1.035	0.582
Smoking (vs. no)	–	–	–	2.284	1.477–3.533	<0.001	–	–	–	2.169	1.394–3.374	<0.001
Alcohol drinking (vs. no)	–	–	–	0.655	0.429–0.998	0.049	–	–	–	0.622	0.405–0.955	0.03
Hypertension (vs. no)	–	–	–	1.757	1.180–2.617	0.006	–	–	–	1.617	1.082–2.416	0.019
Diabetes mellitus (vs. no)	–	–	–	1.286	0.835–1.981	0.254	–	–	–	1.116	0.721–1.726	0.623
Brain age metrics												
Global BAG, per year	1.044	1.022–1.067	<0.001	1.037	1.015–1.059	0.001	–	–	–	–	–	–
Regional BAG in left precentral gyrus, per year	–	–	–	–	–	–	1.016	1.005–1.026	0.003	1.014	1.004–1.025	0.007
Regional BAG in left parahippocampal, per year	–	–	–	–	–	–	1.019	1.008–1.031	<0.001	1.016	1.004–1.028	0.007
Regional BAG in right cerebellum I–IV, per year	–	–	–	–	–	–	1.022	1.007–1.037	0.004	1.021	1.006–1.036	0.007
Model performance												
Concordance index (C-index)	0.772	–	–	0.791	–	–	0.797	–	–	0.811	–	–
AIC	1,532.0	–	–	1,516.4	–	–	1,507.8	–	–	1,498.1	–	–

AIC, Akaike information criterion; BAG, brain age gap; TIV, total intracranial volume; HR, hazard ratio; CI, confidence interval.

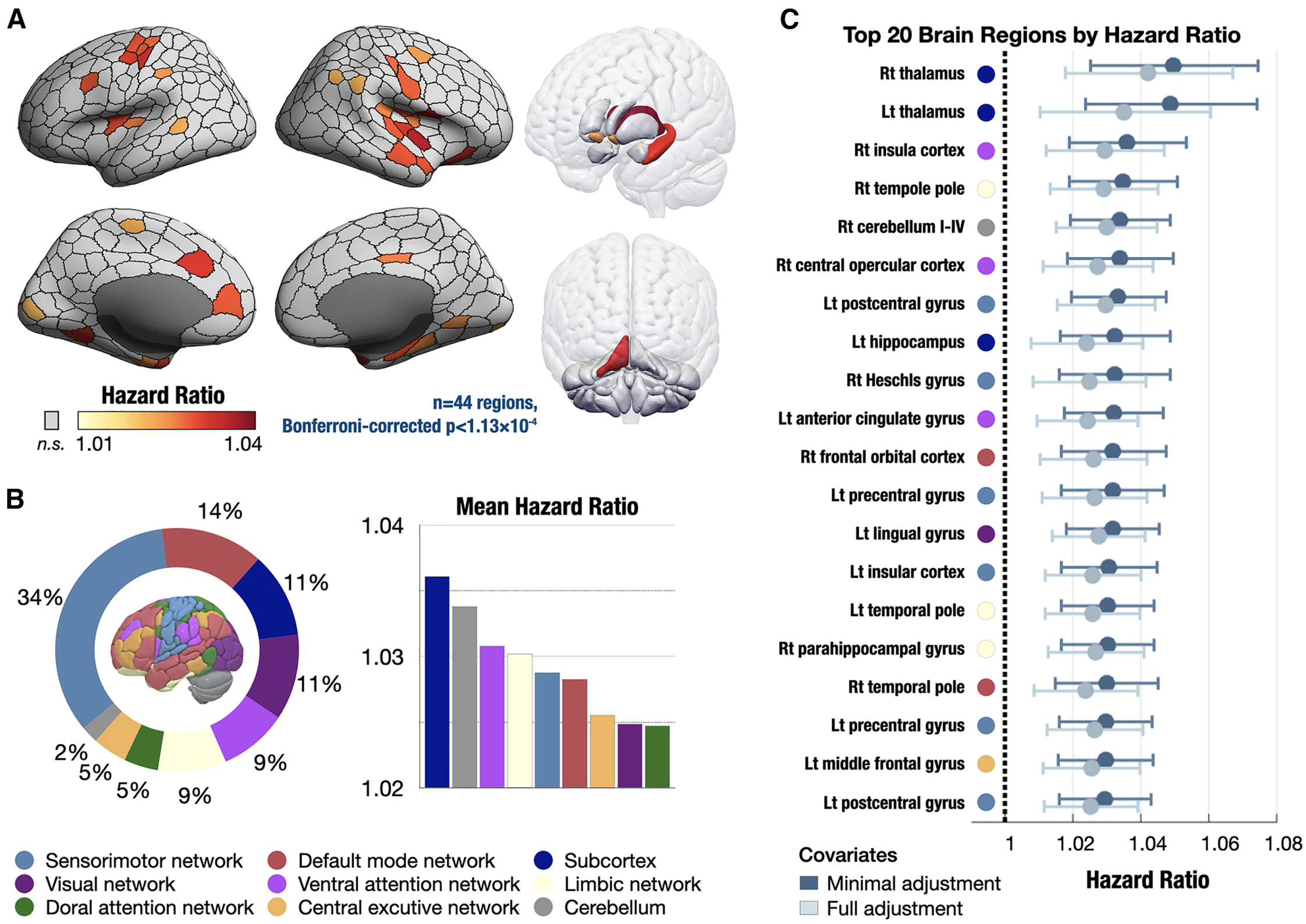

Figure 2. Regional brain age patterns reveal network-specific mortality associations

(A) Spatial distribution of mortality-associated brain regions. Brain surface maps showing 44 regions that achieved Bonferroni-corrected significance ($p < 1.13 \times 10^{-4}$) from 442 regions tested. Color intensity indicates HR strength; non-significant regions are shown in light gray. All models adjusted for age, sex, and TIV.

(B) Network-level distribution of 44 significant regions. Left: pie chart showing proportion of 44 regions by brain network, with sensorimotor network (34%) and default mode network (14%) predominating. Right: the bar plot shows the mean HR of those significant regions within each network.

(C) Forest plot of the top 20 brain regions ranked by HR from each Cox regression model, displaying hazard ratios with 95% confidence intervals for minimally adjusted (light points: age, sex, and TIV) and fully adjusted (dark points: additional covariates including education, smoking, alcohol, hypertension, and diabetes) models.

BAG, brain age gap; HR, hazard ratio; TIV, total intracranial volume; Lt/Rt, left/right hemisphere.

including coagulation pathways for the precentral gyrus, acute-phase response and proteolytic regulation for the parahippocampal gyrus, and notably, iron homeostasis for the cerebellum (Figure S1B). Cross-database validation using Reactome, KEGG, and WikiPathways confirmed the convergent immune/complement mechanism across all regions and provided independent support for the region-specific signatures identified through GO enrichment (Table S7).

Accelerated brain aging predicts cognitive decline

Longitudinal analysis of 444 participants with repeat MRI over a median 8-year interval revealed heterogeneous brain aging trajectories (Figures 5A and 5B). Based on the annual rate of global BAG change adjusted for baseline BAG and scanner upgrade effects, participants were classified into three phenotypes: delayed aging ([DA], $n = 72$, 16.2%), normal aging ([NA], $n = 306$, 68.9%), and accelerated aging ([AA], $n = 66$, 14.9%). The baseline characteristics and aging trajectories are summarized in Table 3. Significant sex differences were observed, with females comprising 69.4% of the delayed aging group compared to 39.4% of the accelerated aging group ($p = 0.002$). No significant differences were observed in age, education, smoking, alcohol consumption, hypertension, diabetes, or MRI scanner upgrade across phenotypes. Baseline cognitive performance was also comparable among the three groups. Cox proportional hazards regression analysis, adjusting for age, sex, and education, indicated that accelerated aging was associated with an increased risk of cognitive decline compared to normal aging (HR = 1.558, 95% CI: 1.016–2.390, $p = 0.042$). Kaplan-Meier survival curves suggested a trend toward earlier onset of cognitive decline in the accelerated aging group, though this did not reach statistical significance in unadjusted log rank testing ($p = 0.125$; Figure 5C).

Proteomic signatures distinguishing extreme aging phenotypes

To investigate the molecular mechanisms underlying differential aging trajectories, we compared the plasma proteomic profiles between extreme phenotypes in 71 participants with available proteomics data (AA: $n = 31$, DA: $n = 40$). Twenty-five proteins

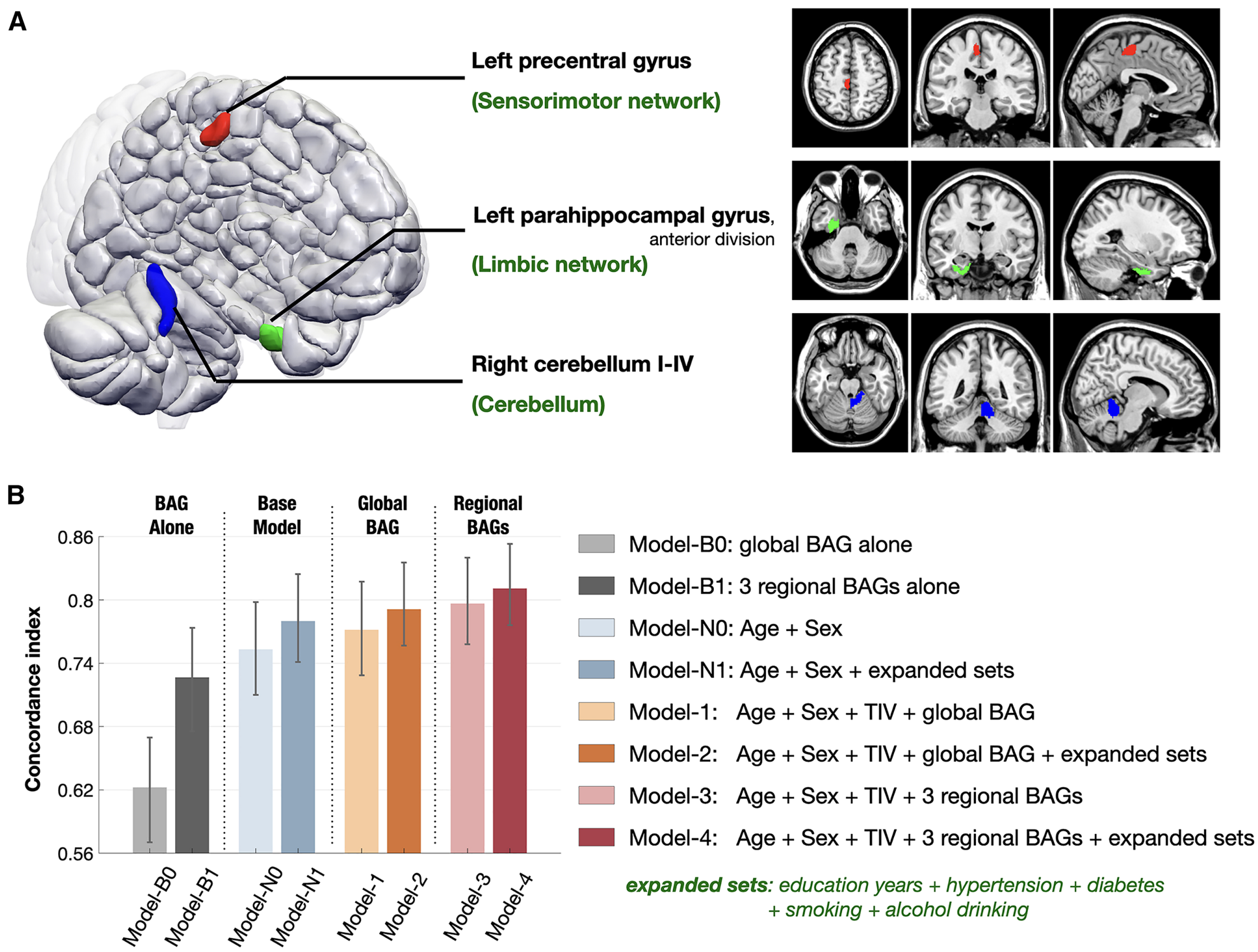

Figure 3. Three-region brain age model optimally predicts mortality risk

(A) Anatomical localization of three optimal regions selected from 44 mortality-associated candidates via forward stepwise regression. Left: 3D brain rendering. Right: MRI slices showing each region in axial, coronal, and sagittal views. Regions: left precentral gyrus (sensorimotor, red), left parahippocampal gyrus (limbic, green), and right cerebellum I–IV (blue).

(B) Model discrimination performance (concordance index). Eight models compared: (1) global BAG only (C = 0.62), (2) three regional BAGs only (C = 0.73), (3) age + sex (C = 0.75), (4) model 3 + risk factors (C = 0.78), (5) model 3 + global BAG (C = 0.77), (6) model 4 + global BAG (C = 0.79), (7) model 3 + 3 regional BAGs (C = 0.80), (8) model 4 + 3 regional BAGs (C = 0.81). Error bars: 95% CI from bootstrap. BAG, brain age gap; C, concordance index.

showed significant differences ($p < 0.05$; Figure 5D; Table S8). Delayed aging was characterized by elevated immunoglobulin proteins, including immunoglobulin lambda variable 4-60 ([IGLV4_60], p = 0.003), immunoglobulin heavy variable 4-4 ([IGHV4_4], p = 0.027), and immunoglobulin lambda constant 7 ([IGLC7], p = 0.037), alongside serum amyloid A2 ([SAA2], p = 0.038) and serum deprivation-response protein ([SDPR], p = 0.011). Accelerated aging showed higher levels of immunoglobulin heavy variable 1-69D ([IGHV1_69D], p = 0.001), keratin 20 ([KRT20], p = 0.001), vascular cell adhesion molecule 1 ([VCAM1], p = 0.024), and complement component 4-binding protein alpha ([C4BPA], p = 0.033).

DISCUSSION

This study investigated whether regional brain age patterns improve mortality prediction beyond global measures and examined the molecular mechanisms and longitudinal trajectories underlying brain aging-mortality associations in 1,240 community-dwelling participants from the ILAS cohort. First, the global BAG constituted an independent predictor of 10-year mortality risk, with each additional year associated with 4.4% increased hazard of death after adjusting for demographic factors. Next, regional brain age analysis across 442 brain regions revealed that a three-region model comprising the left precentral gyrus, left parahippocampal gyrus, and right cerebellum I–IV showed modestly improved discrimination (C-index, 0.81) compared to global brain age approaches (C-index, 0.79). Then, integration of neuroimaging-derived brain age measures with blood biomarkers and discovery-based proteomics identified inflammation as a convergent molecular pathway. Furthermore, longitudinal characterization in 444 participants with repeated neuroimaging revealed heterogeneous brain aging trajectories, with accelerated aging phenotype associated with increased risk of subsequent cognitive decline. These findings demonstrate that brain aging proceeds through network-specific regional vulnerabilities linked to systemic inflammatory processes, with longitudinally defined phenotypes predicting differential clinical outcomes.

Mortality risk stratification in aging populations relies predominantly on chronological age, despite growing recognition

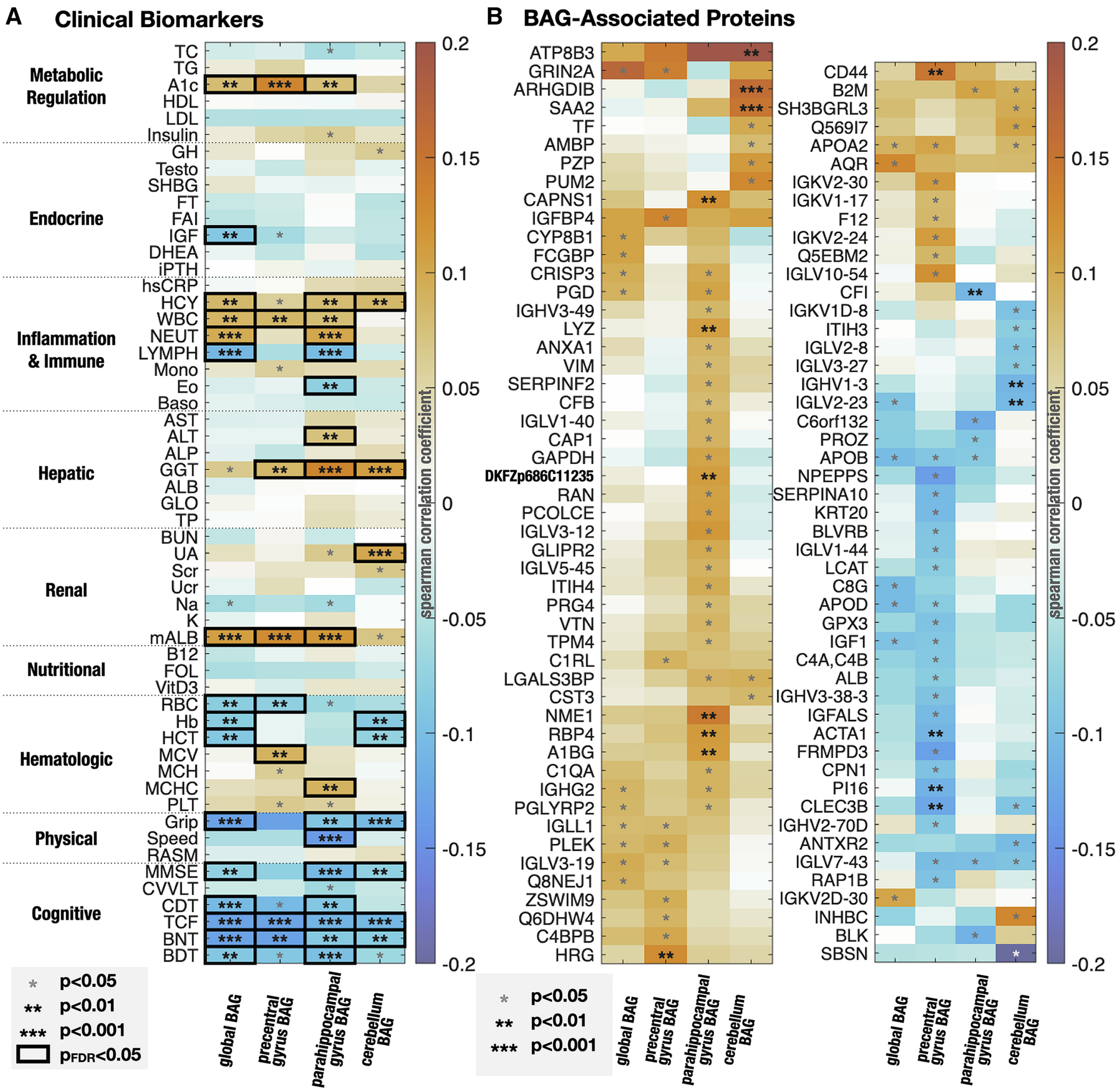

Figure 4. Multi-scale molecular validation of global and regional brain aging

Heatmaps showing partial Spearman correlations between molecular biomarkers (rows) and brain age gaps (columns): global, left precentral gyrus, left parahippocampal gyrus, and right cerebellum. *$p < 0.05$, **$p < 0.01$, ***$p < 0.001$. All correlations adjusted for age, sex, and TIV.

(A) Clinical biomarkers (n = 1,240). Fifty-five biomarkers across nine domains: metabolic, endocrine, inflammation and immune, hepatic, renal, nutritional, hematologic, physical, and cognitive. Black boxes indicate FDR-corrected associations ($q < 0.05$).

(B) Plasma proteomics (n = 606). Display shows 99 of 513 proteins measured, selected for uncorrected $p < 0.05$ with at least one brain age measure. Complete annotations and statistical results are provided in Table S5 for clinical biomarkers and Table S6 for plasma proteins.

BAG, brain age gap; FDR, false discovery rate; TIV, total intracranial volume.

that biological aging heterogeneity determines survival outcomes. Recent plasma proteomic studies established that organ-specific aging—particularly brain and immune system aging—predicts mortality independently of chronological age, with extremely aged brains conferring 182% increased death risk.[17] However, these breakthrough studies employed proteomics-derived brain age rather than direct neuroimaging assessment, leaving unresolved whether regional neuroanatomical patterns enhance prognostic precision beyond global measures. We found that regional brain age signatures derived from structural MRI provided modest additional discriminative value over global brain age for mortality prediction. Beyond discrimination, region-specific biomarker and proteomic correlates diverged across brain regions rather than converging on a single global signature, extending prior proteomic studies of brain and organ aging, which have primarily characterized correlates of global brain age, to a spatially resolved level. Longitudinally defined phenotypes further showed distinct proteomic profiles associated with differential cognitive trajectories. These results add a regionally resolved, multi-scale dimension to the association between brain aging and mortality within a single cohort.

CellPress
OPEN ACCESS

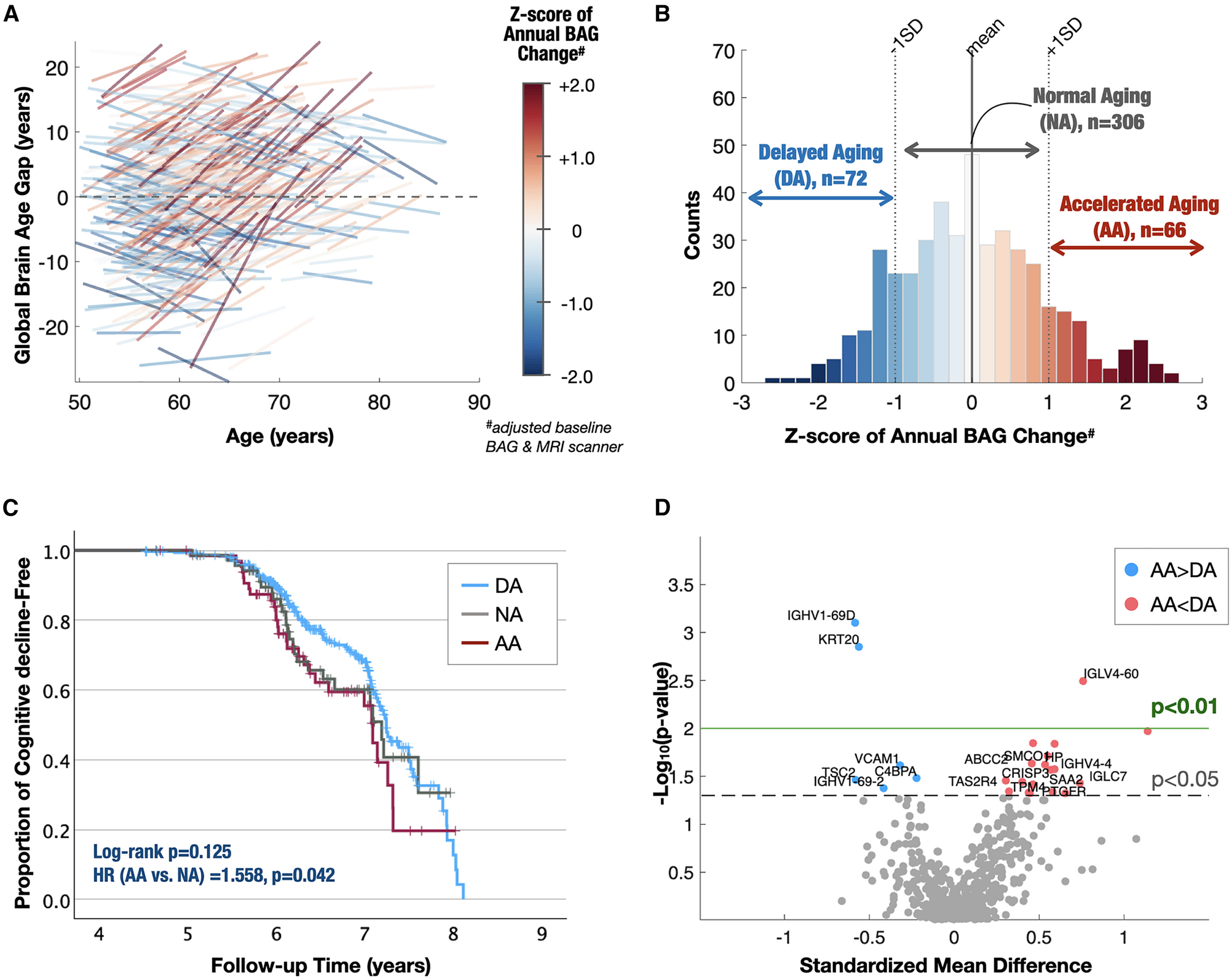

Figure 5. Longitudinal aging phenotypes and clinical outcomes

(A) Individual brain age trajectories over 8-year follow-up (n = 444 participants with repeat MRI). Spaghetti plot showing brain age gap vs. chronological age, with each line representing one participant, color-coded by adjusted Z score of annual rate of BAG change.

(B) Phenotype distribution based on trajectory slopes. Histogram showing frequency distribution of annual global BAG change rates, defining three phenotypes: DA (blue, n = 72), NA (gray, n = 306), and AA (red, n = 66).

(C) Cognitive outcomes by brain aging phenotype. Kaplan-Meier curves showing cognitive decline-free survival (cognitive decline defined as top 10% decline in any cognitive domain). Accelerated aging showed a trend toward shorter cognitive decline-free survival in the Kaplan-Meier analysis and was associated with increased cognitive decline risk in adjusted Cox regression. Cross marks indicate censored observations. HR indicates the hazard ratio for AA versus NA from adjusted Cox regression model.

(D) Proteomic signatures distinguishing phenotypes. Volcano plot comparing protein difference between AA and DA groups. Blue points indicate proteins elevated in AA, red points indicate proteins elevated in DA, and gray points indicate non-significant proteins. The dashed and solid horizontal lines mark p = 0.05 and p = 0.01 significance thresholds, respectively. BAG, brain age gap; DA/NA/AA, delayed/normal/accelerated aging; MRI, magnetic resonance imaging.

Regional brain age architecture captured mortality-relevant information that whole-brain averaging obscured, extending recent recognition of brain aging heterogeneity to survival outcomes. While Yang et al. identified five distinct atrophy patterns across 49,482 individuals, their patterns required complex deep learning decomposition without direct mortality optimization.[18] Our forward stepwise selection distilled the 44 mortality-associated regions to three anatomically interpretable structures—left precentral gyrus (primary motor cortex), left parahippocampal gyrus (medial temporal memory system), and right cerebellum I–IV—showing modestly improved discrimination (C-index, 0.81) compared to global brain age (C-index, 0.79). This regional pattern is broadly consistent with emerging evidence that brain aging proceeded through network-specific vulnerabilities rather than uniform deterioration,[19,20] with particular regions demonstrating selective susceptibility to the “last-in, first-out” principle linking late-developing areas to accelerated age-related degeneration. The concentration of mortality-associated regions within the sensorimotor network (34% of significant regions) reflected motor system integrity as a fundamental survival determinant,

Table 3. Characteristics of longitudinal brain aging phenotype

Variables	Delayed aging (*n* = 72)	Normal aging (*n* = 306)	Accelerated aging (*n* = 66)	*p* value
Demographics at baseline				
Age, years	57.8 ± 7.0	59.5 ± 6.2	59.0 ± 5.4	0.119
Female, *n* (%)	50 (69.4%)	162 (52.9%)	26 (39.4%)	0.002
Education, years	9.2 ± 4.5	8.8 ± 4.5	9.9 ± 4.7	0.217
Lifestyle and risk factors				
Smoking, *n* (%)	8 (11.1%)	34 (11.1%)	11 (16.7%)	0.438
Alcohol drinking, *n* (%)	20 (27.8%)	123 (40.2%)	27 (40.9%)	0.133
Hypertension, *n* (%)	16 (22.2%)	92 (30.1%)	26 (39.4%)	0.090
Diabetes mellitus, *n* (%)	4 (5.6%)	32 (10.5%)	10 (15.2%)	0.180
Brain age metrics				
Baseline global BAG, years	−1.26 ± 9.41	−0.46 ± 9.30	−0.55 ± 9.59	0.542[a]
Follow-up intervals, years (MRI scans)	7.85 ± 1.38	7.90 ± 1.24	7.71 ± 1.39	0.553
Annual BAG change (ΔBAG), years	−0.42 ± 0.28	0.32 ± 0.33	1.20 ± 0.37	<0.001[b]
MRI upgrade, *n* (%)	30 (41.7%)	133 (43.5%)	28 (42.4%)	0.957
Cognitive performance at baseline				
MMSE score	27.5 ± 2.4	27.4 ± 2.5	27.8 ± 3.0	0.988[c]
CVVLT score	6.9 ± 1.9	7.2 ± 1.7	7.2 ± 1.6	0.103[c]
CDT score	8.8 ± 1.9	8.5 ± 1.9	8.8 ± 1.9	0.851[c]
TCF score	32.6 ± 6.2	32.9 ± 4.4	32.8 ± 4.8	0.335[c]
BNT score	13.3 ± 1.9	13.2 ± 2.1	13.4 ± 2.0	0.598[c]
BDT score	4.3 ± 1.7	4.3 ± 1.7	4.4 ± 1.5	0.404[c]
Cognitive outcome				
Cognitive decline, n (%)	26 (36.1%)	104 (34.0%)	28 (42.4%)	0.428
CVVLT decline, n (%)	7 (9.7%)	30 (9.8%)	7 (10.6%)	0.979
CDT decline, n (%)	8 (11.1%)	29 (9.5%)	7 (10.6%)	0.897
TCF decline, n (%)	4 (5.6%)	33 (10.8%)	7 (10.6%)	0.401
BNT decline, n (%)	6 (8.3%)	30 (9.8%)	8 (12.1%)	0.754
BDT decline, n (%)	7 (9.7%)	30 (9.8%)	7 (10.6%)	0.979
Follow-up intervals, years (cognitive assessments)	6.34 ± 0.71	6.43 ± 0.72	6.32 ± 0.66	0.381

BAG, brain age gap; CVVLT, Chinese version Verbal Learning Test; CDT; clock drawing test; TCF, Taylor Complex Figure test; BNT, Boston Naming test; BDT, Backward Digit test.
[a]ANCOVA test, adjusted for age, sex, and total intracranial volume.
[b]ANCOVA test, adjusted for age and sex.
[c]ANCOVA test, adjusted for age, sex, and education years.

consistent with epidemiological evidence linking gait speed, grip strength, and motor decline to mortality risk.[21,22] The precentral gyrus governs voluntary movement control, with its accelerated aging predicting frailty cascades culminating in mortality.[23] The parahippocampal gyrus integrated memory and spatial orientation, showing particular vulnerability to age-related neurodegeneration with implications for functional independence.[24] The cerebellum's contribution extended beyond motor coordination to cognitive processing and autonomic regulation, with cerebellar vulnerability increasingly recognized as a driver of late-life physiological instability.[25,26] This parsimonious three-region model offered practical advantages over multi-dimensional aging indices, providing interpretable biomarkers amenable to clinical implementation while capturing prognostically relevant heterogeneity.

Multi-scale integration of neuroimaging-derived brain age, blood biomarkers, and discovery proteomics identified systemic inflammation as a dominant pathway associated with regional neural aging and mortality, a finding converging with recent plasma proteomic organ aging studies. Liu et al. reported undulating changes in plasma proteome across brain aging, identifying BCAN and GDF15 as key biomarkers with peaks at ages 57, 70, and 78 years.[27] Similarly, Casanova et al. identified 33 proteins associated with the global BAG in an aged and ethnically diverse cohort, with GDF15 and SEVP1 among the most robust positive associations.[7] The most consistent BAG-associated proteins reported in these two studies were low-abundance signaling proteins, including growth factors (GDF15 and FGF21) and glial markers (GFAP and CHI3L1). None of these proteins were among those detected in our mass spectrometry-based

analysis, which instead identified higher-abundance plasma proteins associated with the global BAG, including apolipoproteins (APOA2, APOB, and APOD), immunoglobulin variants, complement C8G, and IGF-1. This divergence at the individual-protein level is consistent with well-recognized differences in protein coverage across proteomic platforms, whereby the targeted platforms used in the two previous studies preferentially quantify low-abundance signaling proteins, while mass spectrometry-based untargeted approaches primarily capture higher-abundance plasma proteins. Notwithstanding these platform-related differences, convergence emerged at the pathway level, with inflammatory and immune activation pathways identified as common correlates linking the plasma proteome to brain aging across all three studies. While these prior studies characterized such pathways in relation to global brain age, our neuroimaging-anchored approach additionally examined whether the same pathways were detectable at the level of specific brain regions: gene ontology analysis revealed immune response pathways—particularly complement activation and adaptive immune processes—as the most enriched themes across all mortality-associated brain regions; blood biomarker profiling showed homocysteine, neutrophil counts, and white blood cell counts consistently associated with regional BAGs. At the individual-protein level, cross-regional consistency was limited, with immunoglobulin lambda variable 7-43 (IGLV7-43) being the only protein showing nominal association across all three regional BAGs, congruent with the immune convergent theme. In the longitudinal cohort, discovery proteomics identified distinct immunoglobulin profiles distinguishing accelerated from delayed aging. This convergence across molecular scales was consistent with the inflammaging framework positioning chronic immune dysregulation as a contributing process linking systemic and neural aging,[3] recently reinforced by evidence that inflammaging drove neurodegenerative progression through persistent microglial activation and cytokine release.[28,29] Critically, our approach identified region-specific molecular signatures suggestive of distinct vulnerabilities beyond global inflammatory signatures. The precentral gyrus demonstrated enrichment for coagulation and fibrinolysis pathways, reflecting motor cortex microvascular vulnerability where vascular-motor coupling integrity determines mobility and survival,[30,31] a pathway undetectable through plasma proteomics. The parahippocampal gyrus showed proteolytic regulation and tissue repair pathway enrichment, suggesting disturbed protein homeostasis in medial temporal structures. The cerebellum exhibited iron homeostasis pathway enrichment, with iron dyshomeostasis contributing to oxidative stress and functional decline.[32] These region-specific signatures provide candidate directions for targeted interventions impossible to identify through global measures and may inform future precision geromedicine approaches.

The region-specific molecular signatures observed here can be interpreted in the context of established mechanisms of brain-periphery communication during aging. Communication between the brain and peripheral circulation is bidirectional and is mediated by multiple routes, including transmigration across the blood-brain barrier (BBB), signaling through brain endothelial cells, and exchange at the choroid plexus.[7,14] The BBB regulates the transport of molecules into and out of the central nervous system, and neuroimaging evidence in aging humans has demonstrated regional BBB permeability changes, most notably in the hippocampus.[33,34] Systemic factors including chronic inflammation, vascular dysregulation, and metabolic disturbances have been positioned as upstream mediators of brain homeostasis and age-related change.[35] Regional brain aging patterns may, therefore, reflect the interaction between shared systemic aging processes and region-specific vulnerabilities, with motor cortex microvasculature, medial temporal protein clearance, and cerebellar iron handling each subject to distinct patterns of vulnerability that could plausibly generate the region-specific plasma protein signatures observed here. Because our findings are based on cross-sectional correlations rather than direct measurement of blood-brain protein exchange, these mechanistic interpretations remain hypothesis generating, and further work incorporating direct assessment of regional BBB permeability and brain-derived plasma proteins would be needed to establish causal pathways.

Longitudinal characterization in 444 participants with repeated neuroimaging revealed substantial heterogeneity in brain aging trajectories, extending cross-sectional heterogeneity findings to temporal dynamics while relating phenotypes to proteomic signatures and cognitive outcomes. Recent studies identified heterogeneous aging patterns using latent class analysis and machine learning,[18] but longitudinal phenotyping with integrated proteomic characterization remained unexplored. In this longitudinal subgroup, females comprised the majority of delayed aging participants but a minority of accelerated aging individuals, consistent with evidence that males exhibit faster brain structural decline[36] and sex-specific glial responses to inflammaging.[29] Accelerated aging conferred elevated cognitive decline risk (HR = 1.558 vs. normal aging), aligning with associations between accelerated atrophy trajectories and cognitive deterioration.[37,38] Critically, proteomic profiling identified 25 proteins distinguishing extreme phenotypes, with delayed aging characterized by elevated immunoglobulins (IGLV4_60, IGHV4_4, and IGLC7) and serum amyloid A2 suggesting preserved adaptive immune responses,[39] while accelerated aging demonstrated elevated endothelial dysfunction markers and complement regulatory proteins. These divergent molecular signatures indicated fundamentally different biological states rather than merely different rates along a common trajectory, differing from existing models that position brain aging as uniform acceleration or deceleration. This phenotype-proteomic integration enables personalized monitoring, with accelerated agers requiring intensive surveillance and targeted interventions addressing their specific endothelial-immune dysregulation profile.

The present findings position regional neuroimaging-proteomic integration as superior to either modality alone for mortality prediction and biological characterization. Recent proteomic organ aging studies demonstrated brain age as the strongest single mortality predictor among 11 organs,[17] with youthful brains conferring 40% mortality risk reduction and aged brains conferring 182% risk increase. While these findings established plasma proteomics as a scalable brain aging tool, our direct neuroimaging assessment achieved three critical advances: (1) superior discriminative performance through regional pattern optimization specifically for mortality (C-index 0.81 vs. published global brain age C-indices of

0.77–0.79), (2) revelation of region-specific pathways (motor cortex coagulation, medial temporal proteostasis, and cerebellar iron homeostasis) undetectable through blood-based global measures, and (3) characterization in an East Asian cohort, extending predominantly European-ancestry findings. Beyond mortality prediction, our integration addressed gaps in existing heterogeneity research. While Yang et al. identified five atrophy patterns requiring complex deep learning,[18] our three-region model achieved superior mortality discrimination with anatomically interpretable, clinically implementable biomarkers. Previous longitudinal brain aging studies examined trajectories[38] or cross-sectional patterns,[18] but not their proteomic underpinnings. Our phenotype-proteomic integration revealed that aging heterogeneity reflected distinct biological processes requiring phenotype-specific interventions. This multi-scale framework established neuroimaging-proteomic integration as essential for precision geromedicine, with neither modality alone capturing the additional biological detail achieved through their combination.

Circulating proteomic biomarkers associated with regional brain aging enable blood-based risk assessment without neuroimaging requirements, facilitating population-scale screening while preserving biological interpretability. Combined with recent demonstrations that plasma proteomics can estimate organ-specific aging, our region-specific pathway characterization suggests a hybrid strategy: initial blood-based screening to identify high-risk individuals, followed by targeted neuroimaging to characterize the affected brain regions and intervention planning. Regional specificity indicates that system-targeted interventions may prove superior to global approaches—motor cortex vulnerability suggests vascular optimization through blood pressure control, medial temporal proteostasis dysfunction indicates protein clearance enhancement via autophagy induction, and cerebellar iron dysregulation points to antioxidant or chelation strategies. Critical research priorities include: (1) validating regional brain age signatures across diverse populations, (2) examining causal relationships through intervention trials targeting identified pathways, (3) developing simplified blood-based algorithms for scalable screening, and (4) testing whether interventions modifying regional brain age translate to mortality benefit. Success would transform brain aging from an observational biomarker into an actionable clinical target, enabling biologically informed risk stratification, mechanistically guided interventions, and personalized monitoring to extend health span.

Limitations of the study

Several considerations informed interpretation and future directions. This single-cohort Taiwanese study requires validation across diverse populations to establish the generalizability of regional signatures and proteomic profiles. Proteomic data availability in approximately half the cohort, with further limitation in extreme phenotype characterization, constrained statistical power for molecular discovery. Given the relatively low mortality rate observed in this cohort, we were unable to examine associations with cause-specific mortality, which would require longer follow-up. The longitudinal cohort exhibited a healthy-volunteer attrition pattern, with returning participants being younger and healthier at baseline than non-returning participants. Because the aging phenotype classification was defined relative to the longitudinal sample distribution, associations reported for accelerated aging should be interpreted within this returning subgroup, and effect estimates in the broader baseline population may differ in magnitude. In addition, our mass spectrometry-based untargeted proteomic profiling preferentially quantified higher-abundance plasma proteins and did not include several low-abundance signaling proteins previously identified as brain age markers, such as GDF15, GFAP, and FGF21. A systematic coverage analysis further indicated that the measured panel was significantly enriched for extracellular-space proteins but under-represented for intracellular, nuclear, and plasma membrane proteins, as well as several regulatory protein classes (Table S9). This platform-related coverage difference likely accounts for the limited individual-protein overlap with prior brain age proteomic studies. Nevertheless, pathway-level convergence with previous findings is consistent with the inflammatory and immune pathways identified in our analysis, and future studies incorporating complementary proteomic platforms could extend individual-protein-level comparisons. The present brain age modeling was based on gray matter volume derived from T1-weighted MRI. Multimodal extensions incorporating white matter integrity, diffusion metrics, or tract-based connectivity between mortality-associated regions could enrich the biological granularity of brain age estimation and represent a valuable direction for future studies. Alcohol consumption in the present study was ascertained as a binary variable (ever vs. never), which does not distinguish between moderate and excessive drinking patterns. Although alcohol consumption was included as a covariate in the fully adjusted models, this crude categorization may not fully capture the confounding influence of alcohol on brain aging. More broadly, the regional biomarker and proteomic associations reported throughout this study represent statistical correlates of regional brain aging rather than validated mechanisms, and establishing the underlying biological pathways would require complementary experimental validation. The regional patterns described here should accordingly be interpreted as hypothesis generating rather than as mechanistic evidence. Recent advances in multi-omic aging clocks[27] and organ-specific aging indices[17] suggest future integration with epigenetic, metabolomic, and additional proteomic platforms as a direction for further characterizing these associations. Despite these limitations, convergent findings across neuroimaging, molecular, and longitudinal analyses provide a regionally resolved and hypothesis-generating characterization of brain aging heterogeneity and its association with mortality.

RESOURCE AVAILABILITY

Lead contact

Requests for further information and resources should be directed to and will be fulfilled by the lead contact, Liang-Kung Chen (lkchen2@vghtpe.gov.tw).

Materials availability

This study did not generate new unique reagents.

Data and code availability

- Plasma proteomic data reported in this paper have been deposited to the MassIVE repository via ProteomeXchange with the dataset identifiers MassIVE: MSV000103015 and ProteomeXchange: PXD083183. Because of privacy and ethical restrictions on individual-level human

CellPress
OPEN ACCESS

cohort data, raw image data are not publicly deposited. Requests for individual-level raw data will be considered by the lead contact, Liang-Kung Chen (lkchen2@vghtpe.gov.tw), when accompanied by a justified scientific proposal and documented ethical approval.

- Neuroimaging and machine learning analyses, including brain age prediction modeling, were implemented in Python and MATLAB using established open-source packages, including SPM12 for neuroimaging preprocessing and scikit-learn for support vector regression modeling, without development of new algorithms or custom analytical code.
- Any additional information required to reanalyze the data reported in this paper is available from the lead contact upon request.

ACKNOWLEDGMENTS

This study was supported by the National Science and Technology Council (NSTC 115-2321-B-A49-011 to L.-K.C., 115-2321-B-A49-013 to C.-P.L., and 115-2314-B-A49-072-MY2 to P.-L.L.), the Taipei Veterans General Hospital (115VACS-001), Taipei City Hospital (TPCH-115-05), and the Department of Health, Taipei City Government (11501-62-021). The authors thank the Interdisciplinary Research Center for Healthy Longevity of National Yang Ming Chiao Tung University from The Featured Areas Research Center Program within the framework of the Higher Education Sprout Project by the Ministry of Education (MOE) in Taiwan for supporting the research work.

AUTHOR CONTRIBUTIONS

Conceptualization, L.-K.C.; methodology, P.-L.L. and Y.-L.H.; investigation, P.-L.L. and Y.-L.H.; resources, C.-K.L., W.-J.L., L.-N.P., F.-Y.H., C.-P.L., and L.-K.C.; data curation, W.-J.L. and L.-N.P.; writing – original draft, P.-L.L. and Y.-L.H.; writing – review and editing, L.-K.C.; visualization, P.-L.L.; supervision, C.-K.L., W.-J.L., F.-Y.H., C.-P.L., and L.-K.C.; project administration, L.-K.C.; funding acquisition, C.-P.L. and L.-K.C.

DECLARATION OF INTERESTS

The authors declare no competing interests.

DECLARATION OF GENERATIVE AI AND AI-ASSISTED TECHNOLOGIES IN THE WRITING PROCESS

During the preparation of this work, the authors used Claude (Anthropic) to assist with English language editing and refinement during manuscript preparation. After using this tool, the authors reviewed and edited the content as needed and take full responsibility for the content of the publication.

STAR★METHODS

Detailed methods are provided in the online version of this paper and include the following:

- KEY RESOURCES TABLE
- EXPERIMENTAL MODEL AND STUDY PARTICIPANT DETAILS
  - Study population
- METHOD DETAILS
  - Brain MRI acquisition
  - Clinical biomarkers and physical function assessments
  - Proteomic analysis
  - Cognitive assessments
  - Image processing and feature extraction
  - Brain age prediction model
  - Longitudinal brain aging phenotypes
- QUANTIFICATION AND STATISTICAL ANALYSIS
  - Descriptive statistics and group comparisons
  - Global and regional BAG associations with mortality risk
  - Molecular correlates of brain aging
  - Longitudinal brain aging phenotypes and cognitive outcomes

SUPPLEMENTAL INFORMATION

Supplementary data related to this article can be found online at https://doi.org/10.1016/j.xcrm.2026.103096.

Received: June 2, 2026
Revised: August 2, 2026
Accepted: September 10, 2026
Published: October 7, 2026

REFERENCES

1. Mitnitski, A.B., Mogilner, A.J., MacKnight, C., and Rockwood, K. (2002). The mortality rate as a function of accumulated deficits in a frailty index. Mech. Ageing Dev. *123*, 1457–1460. https://doi.org/10.1016/s0047-6374(02)00082-9.

2. Elliott, M.L., Caspi, A., Houts, R.M., Ambler, A., Broadbent, J.M., Hancox, R.J., Harrington, H., Hogan, S., Keenan, R., Knodt, A., et al. (2021). Disparities in the pace of biological aging among midlife adults of the same chronological age have implications for future frailty risk and policy. Nat. Aging *1*, 295–308. https://doi.org/10.1038/s43587-021-00044-4.

3. Ferrucci, L., Gonzalez-Freire, M., Fabbri, E., Simonsick, E., Tanaka, T., Moore, Z., Salimi, S., Sierra, F., and de Cabo, R. (2020). Measuring biological aging in humans: A quest. Aging Cell *19*, e13080. https://doi.org/10.1111/acel.13080.

4. Anderton, B.H. (2002). Ageing of the brain. Mech. Ageing Dev. *123*, 811–817. https://doi.org/10.1016/s0047-6374(01)00426-2.

5. Cole, J.H., and Franke, K. (2017). Predicting Age Using Neuroimaging: Innovative Brain Ageing Biomarkers. Trends Neurosci. *40*, 681–690. https://doi.org/10.1016/j.tins.2017.10.001.

6. Gaser, C., Kalc, P., and Cole, J.H. (2024). A perspective on brain-age estimation and its clinical promise. Nat. Comput. Sci. *4*, 744–751. https://doi.org/10.1038/s43588-024-00659-8.

7. Casanova, R., Walker, K.A., Justice, J.N., Anderson, A., Duggan, M.R., Cordon, J., Barnard, R.T., Lu, L., Hsu, F.C., Sedaghat, S., et al. (2024). Associations of plasma proteomics and age-related outcomes with brain age in a diverse cohort. GeroScience *46*, 3861–3873. https://doi.org/10.1007/s11357-024-01112-4.

8. Zhang, R., Yi, F., Mao, H., Huang, Z., Wang, K., and Zhang, J. (2025). Brain age gap as a predictive biomarker that links aging, lifestyle, and neuropsychiatric health. Commun. Med. *5*, 441. https://doi.org/10.1038/s43856-025-01100-5.

9. Cole, J.H., Ritchie, S.J., Bastin, M.E., Valdés Hernández, M.C., Muñoz Maniega, S., Royle, N., Corley, J., Pattie, A., Harris, S.E., Zhang, Q., et al. (2018). Brain age predicts mortality. Mol. Psychiatry *23*, 1385–1392. https://doi.org/10.1038/mp.2017.62.

10. Franke, K., and Gaser, C. (2012). Longitudinal Changes in Individual BrainAGE in Healthy Aging, Mild Cognitive Impairment, and Alzheimer's Disease (GeroPsych).

11. Kaufmann, T., van der Meer, D., Doan, N.T., Schwarz, E., Lund, M.J., Agartz, I., Alnæs, D., Barch, D.M., Baur-Streubel, R., Bertolino, A., et al. (2019). Common brain disorders are associated with heritable patterns of apparent aging of the brain. Nat. Neurosci. *22*, 1617–1623. https://doi.org/10.1038/s41593-019-0471-7.

12. Cole, J.H., Marioni, R.E., Harris, S.E., and Deary, I.J. (2019). Brain age and other bodily 'ages': implications for neuropsychiatry. Mol. Psychiatry *24*, 266–281. https://doi.org/10.1038/s41380-018-0098-1.

13. Fjell, A.M., McEvoy, L., Holland, D., Dale, A.M., and Walhovd, K.B.; Alzheimer's Disease Neuroimaging Initiative (2014). What is normal in normal aging? Effects of aging, amyloid and Alzheimer's disease on the cerebral cortex and the hippocampus. Prog. Neurobiol. *117*, 20–40. https://doi.org/10.1016/j.pneurobio.2014.02.004.

14. Bieri, G., Schroer, A.B., and Villeda, S.A. (2023). Blood-to-brain communication in aging and rejuvenation. Nat. Neurosci. *26*, 379–393. https://doi.org/10.1038/s41593-022-01238-8.

15. Guest, P.C., Guest, F.L., and Martins-de Souza, D. (2016). Making Sense of Blood-Based Proteomics and Metabolomics in Psychiatric Research. Int. J. Neuropsychopharmacol. *19*, pyv138. https://doi.org/10.1093/ijnp/pyv138.

16. Vidal-Pineiro, D., Wang, Y., Krogsrud, S.K., Amlien, I.K., Baaré, W.F.C., Bartres-Faz, D., Bertram, L., Brandmaier, A.M., Drevon, C.A., Düzel, S., et al. (2021). Individual variations in 'brain age' relate to early-life factors more than to longitudinal brain change. eLife *10*, e69995. https://doi.org/10.7554/eLife.69995.

17. Oh, H.S.H., Le Guen, Y., Rappoport, N., Urey, D.Y., Farinas, A., Rutledge, J., Channappa, D., Wagner, A.D., Mormino, E., Brunet, A., et al. (2025). Plasma proteomics links brain and immune system aging with healthspan and longevity. Nat. Med. *31*, 2703–2711. https://doi.org/10.1038/s41591-025-03798-1.

18. Yang, Z., Wen, J., Erus, G., Govindarajan, S.T., Melhem, R., Mamourian, E., Cui, Y., Srinivasan, D., Abdulkadir, A., Parmpi, P., et al. (2024). Brain aging patterns in a large and diverse cohort of 49,482 individuals. Nat. Med. *30*, 3015–3026. https://doi.org/10.1038/s41591-024-03144-x.

19. Manuello, J., Min, J., McCarthy, P., Alfaro-Almagro, F., Lee, S., Smith, S., Elliott, L.T., Winkler, A.M., and Douaud, G. (2024). The effects of genetic and modifiable risk factors on brain regions vulnerable to ageing and disease. Nat. Commun. *15*, 2576. https://doi.org/10.1038/s41467-024-46344-2.

20. Douaud, G., Groves, A.R., Tamnes, C.K., Westlye, L.T., Duff, E.P., Engvig, A., Walhovd, K.B., James, A., Gass, A., Monsch, A.U., et al. (2014). A common brain network links development, aging, and vulnerability to disease. Proc. Natl. Acad. Sci. USA *111*, 17648–17653. https://doi.org/10.1073/pnas.1410378111.

21. Gale, C.R., Martyn, C.N., Cooper, C., and Sayer, A.A. (2007). Grip strength, body composition, and mortality. Int. J. Epidemiol. *36*, 228–235. https://doi.org/10.1093/ije/dyl224.

22. Studenski, S., Perera, S., Patel, K., Rosano, C., Faulkner, K., Inzitari, M., Brach, J., Chandler, J., Cawthon, P., Connor, E.B., et al. (2011). Gait speed and survival in older adults. JAMA *305*, 50–58. https://doi.org/10.1001/jama.2010.1923.

23. Fried, L.P., Tangen, C.M., Walston, J., Newman, A.B., Hirsch, C., Gottdiener, J., Seeman, T., Tracy, R., Kop, W.J., Burke, G., et al. (2001). Frailty in older adults: evidence for a phenotype. J. Gerontol. A Biol. Sci. Med. Sci. *56*, M146–M156. https://doi.org/10.1093/gerona/56.3.m146.

24. Burke, S.N., Gaynor, L.S., Barnes, C.A., Bauer, R.M., Bizon, J.L., Roberson, E.D., and Ryan, L. (2018). Shared Functions of Perirhinal and Parahippocampal Cortices: Implications for Cognitive Aging. Trends Neurosci. *41*, 349–359. https://doi.org/10.1016/j.tins.2018.03.001.

25. Arleo, A., Bareš, M., Bernard, J.A., Bogoian, H.R., Bruchhage, M.M.K., Bryant, P., Carlson, E.S., Chan, C.C.H., Chen, L.K., Chung, C.P., et al. (2024). Consensus Paper: Cerebellum and Ageing. Cerebellum *23*, 802–832. https://doi.org/10.1007/s12311-023-01577-7.

26. McElroy, C.L., Wang, B., Zhang, H., and Jin, K. (2024). Cerebellum and Aging: Update and Challenges. Aging Dis. *15*, 2345–2360. https://doi.org/10.14336/AD.2024.0220.

27. Liu, W.S., You, J., Chen, S.D., Zhang, Y., Feng, J.F., Xu, Y.M., Yu, J.T., and Cheng, W. (2025). Plasma proteomics identify biomarkers and undulating changes of brain aging. Nat. Aging *5*, 99–112. https://doi.org/10.1038/s43587-024-00753-6.

28. Ajoolabady, A., Pratico, D., Tang, D., Zhou, S., Franceschi, C., and Ren, J. (2024). Immunosenescence and inflammaging: Mechanisms and role in diseases. Ageing Res. Rev. *101*, 102540. https://doi.org/10.1016/j.arr.2024.102540.

29. Müller, L., Di Benedetto, S., and Müller, V. (2025). Neuroimmune dynamics and brain aging: mechanisms and consequences. Front. Aging Neurosci. *17*, 1715045. https://doi.org/10.3389/fnagi.2025.1715045.

30. Sorond, F.A., Kiely, D.K., Galica, A., Moscufo, N., Serrador, J.M., Iloputaife, I., Egorova, S., Dell'Oglio, E., Meier, D.S., Newton, E., et al. (2011). Neurovascular coupling is impaired in slow walkers: the MOBILIZE Boston Study. Ann. Neurol. *70*, 213–220. https://doi.org/10.1002/ana.22433.

31. Knoll, C., Doehler, J., Northall, A., Schreiber, S., Rotta, J., Mattern, H., and Kuehn, E. (2024). Age-related differences in human cortical microstructure depend on the distance to the nearest vein. Brain Commun. *6*, fcae321. https://doi.org/10.1093/braincomms/fcae321.

32. Ashraf, A., Clark, M., and So, P.W. (2018). The Aging of Iron Man. Front. Aging Neurosci. *10*, 65. https://doi.org/10.3389/fnagi.2018.00065.

33. Montagne, A., Barnes, S.R., Sweeney, M.D., Halliday, M.R., Sagare, A.P., Zhao, Z., Toga, A.W., Jacobs, R.E., Liu, C.Y., Amezcua, L., et al. (2015). Blood-brain barrier breakdown in the aging human hippocampus. Neuron *85*, 296–302. https://doi.org/10.1016/j.neuron.2014.12.032.

34. Sweeney, M.D., Zhao, Z., Montagne, A., Nelson, A.R., and Zlokovic, B.V. (2019). Blood-Brain Barrier: From Physiology to Disease and Back. Physiol. Rev. *99*, 21–78. https://doi.org/10.1152/physrev.00050.2017.

35. Pluvinage, J.V., and Wyss-Coray, T. (2020). Systemic factors as mediators of brain homeostasis, ageing and neurodegeneration. Nat. Rev. Neurosci. *21*, 93–102. https://doi.org/10.1038/s41583-019-0255-9.

36. Vinke, E.J., de Groot, M., Venkatraghavan, V., Klein, S., Niessen, W.J., Ikram, M.A., and Vernooij, M.W. (2018). Trajectories of imaging markers in brain aging: the Rotterdam Study. Neurobiol. Aging *71*, 32–40. https://doi.org/10.1016/j.neurobiolaging.2018.07.001.

37. Sluimer, J.D., van der Flier, W.M., Karas, G.B., Fox, N.C., Scheltens, P., Barkhof, F., and Vrenken, H. (2008). Whole-brain atrophy rate and cognitive decline: longitudinal MR study of memory clinic patients. Radiology *248*, 590–598. https://doi.org/10.1148/radiol.2482070938.

38. Ly, M., Yu, G., Son, S.J., Pascoal, T., and Karim, H.T.; the Alzheimer's disease Neuroimaging Initiative (2024). Longitudinal accelerated brain age in mild cognitive impairment and Alzheimer's disease. Front. Aging Neurosci. *16*, 1433426. https://doi.org/10.3389/fnagi.2024.1433426.

39. Eklund, K.K., Niemi, K., and Kovanen, P.T. (2012). Immune functions of serum amyloid A. Crit. Rev. Immunol. *32*, 335–348. https://doi.org/10.1615/critrevimmunol.v32.i4.40.

40. Schaefer, A., Kong, R., Gordon, E.M., Laumann, T.O., Zuo, X.N., Holmes, A.J., Eickhoff, S.B., and Yeo, B.T.T. (2018). Local-Global Parcellation of the Human Cerebral Cortex from Intrinsic Functional Connectivity MRI. Cereb. Cortex *28*, 3095–3114. https://doi.org/10.1093/cercor/bhx179.

41. Makris, N., Goldstein, J.M., Kennedy, D., Hodge, S.M., Caviness, V.S., Faraone, S.V., Tsuang, M.T., and Seidman, L.J. (2006). Decreased volume of left and total anterior insular lobule in schizophrenia. Schizophr. Res. *83*, 155–171. https://doi.org/10.1016/j.schres.2005.11.020.

42. Frazier, J.A., Chiu, S., Breeze, J.L., Makris, N., Lange, N., Kennedy, D.N., Herbert, M.R., Bent, E.K., Koneru, V.K., Dieterich, M.E., et al. (2005). Structural brain magnetic resonance imaging of limbic and thalamic volumes in pediatric bipolar disorder. Am. J. Psychiatry *162*, 1256–1265. https://doi.org/10.1176/appi.ajp.162.7.1256.

43. Diedrichsen, J. (2006). A spatially unbiased atlas template of the human cerebellum. Neuroimage *33*, 127–138. https://doi.org/10.1016/j.neuroimage.2006.05.056.

44. Lee, W.J., Liu, L.K., Peng, L.N., Lin, M.H., and Chen, L.K. (2013). Comparisons of sarcopenia defined by IWGS and EWGSOP criteria among older people: results from the I-Lan longitudinal aging study. J. Am. Med. Dir. Assoc. *14*, 528.e1–528.e7. https://doi.org/10.1016/j.jamda.2013.03.019.

45. Mok, E.H.L., Lam, L.C.W., and Chiu, H.F.K. (2004). Category verbal fluency test performance in chinese elderly with Alzheimer's disease. Dement. Geriatr. Cogn. Disord. *18*, 120–124. https://doi.org/10.1159/000079190.

CellPress
OPEN ACCESS

46. Mack, W.J., Freed, D.M., Williams, B.W., and Henderson, V.W. (1992). Boston Naming Test: shortened versions for use in Alzheimer's disease. J. Gerontol. *47*, P154–P158. https://doi.org/10.1093/geronj/47.3.p154.

47. Taylor, L.B. (1969). Localisation of cerebral lesions by psychological testing. Clin. Neurosurg. *16*, 269–287. https://doi.org/10.1093/neurosurgery/16.cn_suppl_1.269.

48. Lezak, M.D., Howieson, D.B., Loring, D.W., Hannay, H.J., and Fischer, J.S. (2004). Neuropsychological Assessment (Oxford University Press).

49. Eknoyan, D., Hurley, R.A., and Taber, K.H. (2012). The clock drawing task: common errors and functional neuroanatomy. J. Neuropsychiatry Clin. Neurosci. *24*, 260–265. https://doi.org/10.1176/appi.neuropsych.12070180.

50. Rouleau, I., Salmon, D.P., Butters, N., Kennedy, C., and McGuire, K. (1992). Quantitative and qualitative analyses of clock drawings in Alzheimer's and Huntington's disease. Brain Cogn. *18*, 70–87. https://doi.org/10.1016/0278-2626(92)90112-y.

51. Lee, P.L., Kuo, C.Y., Wang, P.N., Chen, L.K., Lin, C.P., Chou, K.H., and Chung, C.P. (2022). Regional rather than global brain age mediates cognitive function in cerebral small vessel disease. Brain Commun. *4*, fcac233. https://doi.org/10.1093/braincomms/fcac233.

52. Franke, K., Ziegler, G., Klöppel, S., and Gaser, C.; Alzheimer's Disease Neuroimaging Initiative (2010). Alzheimer's Disease Neuroimaging, I. Estimating the age of healthy subjects from T1-weighted MRI scans using kernel methods: exploring the influence of various parameters. Neuroimage *50*, 883–892. https://doi.org/10.1016/j.neuroimage.2010.01.005.

53. Franke, K., and Gaser, C. (2019). Ten Years of BrainAGE as a Neuroimaging Biomarker of Brain Aging: What Insights Have We Gained? Front. Neurol. *10*, 789. https://doi.org/10.3389/fneur.2019.00789.

54. Yu, Y., Cui, H.Q., Haas, S.S., New, F., Sanford, N., Yu, K., Zhan, D., Yang, G., Gao, J.H., Wei, D., et al. (2024). Brain-age prediction: Systematic evaluation of site effects, and sample age range and size. Hum. Brain Mapp. *45*, e26768. https://doi.org/10.1002/hbm.26768.

STAR★METHODS

KEY RESOURCES TABLE

REAGENT or RESOURCE	SOURCE	IDENTIFIER
**Biological samples**		
Human plasma samples	I-Lan Longitudinal Aging Study (ILAS), Taiwan	N/A
**Chemicals, peptides, and recombinant proteins**		
Acetonitrile, LiChrosolv	Merck	Cat# 1.00029
Formic acid, LiChropur	Supelco (Merck)	Cat# 5.43804
Dithiothreitol	Sigma	Cat# D0632
iodoacetamide	Sigma	Cat# I6125
**Critical commercial assays**		
SMART Digest Kit with SOLAμ HRP SPE plate	Thermo Fisher Scientific	Cat# 60109–103
**Deposited data**		
Plasma proteomic data	This paper	MassIVE: MSV000103015; ProteomeXchange:PXD083183
**Software and algorithms**		
Proteome Discoverer version 2.4	Thermo Fisher Scientific	https://www.thermofisher.com/tw/zt/home/industrial/mass-spectrometry/liquid-chromatography-mass-spectrometry-lc-ms/lc-ms-software/multi-omics-data-analysis/proteome-discoverer-software.html
MATLAB R2021a	The MathWorks	https://matlab.mathworks.com/
SPM12 version 7487	Open source	https://www.fil.ion.ucl.ac.uk/spm/software/spm12/
Python version 3.11	Open source	https://www.python.org/
scikit-learn version 1.6.1	Open source	https://scikit-learn.org/stable/
SPSS version 29	IBM	https://www.ibm.com/products/spss-statistics
JASP version 0.95.1	JASP Team	https://jasp-stats.org/
DAVID Bioinformatics Resources	National Institute of Allergy and Infectious Diseases (NIAID), NIH	https://davidbioinformatics.nih.gov/
**Other**		
Schaefer 400-region cortical atlas	Schaefer et al.[40]	https://github.com/ThomasYeoLab/CBIG/tree/master/stable_projects/brain_parcellation/Schaefer2018_LocalGlobal
Harvard-Oxford subcortical atlas	Makris et al.[41] Frazier et al.[42]	https://fsl.fmrib.ox.ac.uk/fsl/docs/other/datasets.html
SUIT cerebellar atlas	Diedrichsen[43]	https://github.com/DiedrichsenLab/cerebellar_atlases

EXPERIMENTAL MODEL AND STUDY PARTICIPANT DETAILS

Study population

We analyzed participants from the I-Lan Longitudinal Aging Study (ILAS), an ongoing community-based cohort established in 2011.[44] The ILAS recruited community-dwelling adults aged 50 years or older from Yuanshan Township, I-Lan County, Taiwan. Initial enrollment occurred between January 2011 and July 2014, comprising 1,839 participants. At baseline (ILAS-1), participants underwent brain MRI, blood sampling for biomarkers and proteomics, cognitive testing, physical assessments, and clinical interviews. Smoking and alcohol drinking status were ascertained through self-report questionnaires and categorized as ever (current or former) versus

never users. The complete participant selection flowchart was shown at Figure S2. Among 1,839 initially enrolled adults, we excluded 599 participants due to: existing neurological or psychiatric disorders (stroke, dementia, brain tumor, major depression, substance abuse), suboptimal MRI quality, MRI contraindications (claustrophobia, ferromagnetic implants), or incomplete baseline data. The final baseline cohort consisted of 1,240 participants with adequate brain MRI scans and complete assessments for brain age analysis. Sex was included as a covariate in all mortality and biomarker analyses, and sex-stratified associations and sex-by-BAG interaction effects are reported in the Results.

Vital status was ascertained through telephone interviews conducted every three months by trained research nurses. Follow-up extended from baseline enrollment until April 2022. Between 2018 and 2024, we invited participants to return for follow-up assessments (ILAS-3) that included repeated brain MRI and cognitive testing. Among those who returned, 444 participants completed both assessments with optimal MRI quality, forming the longitudinal cohort for analyzing brain aging trajectories. Baseline characteristics of returning and non-returning participants are compared in Table S10.

All participants provided written informed consent. The study protocol received approval from the Institutional Review Boards of National Yang Ming Chiao Tung University (YM103008) and Taipei Veterans General Hospital (2018-05-003 B). All procedures adhered to the principles outlined in the Declaration of Helsinki.

METHOD DETAILS

Brain MRI acquisition

All brain MRI scans were acquired at National Yang Ming Chiao Tung University, Taipei, Taiwan. Baseline T1-weighted anatomical scans (ILAS-1, n = 1,240) were obtained on a Siemens 3 T Magnetom Tim Trio scanner (Siemens Healthcare, Erlangen, Germany) using a 12-channel phased-array head coil. Images were acquired using a three-dimensional magnetization-prepared rapid-acquisition gradient-echo (3D MPRAGE) sequence with the following parameters: repetition time/echo time/inversion time = 3500/3.5/1100 ms, flip angle = 7°, field of view = 256 × 256 mm^2, matrix size = 256 × 256, number of excitations = 1, 192 sagittal slices with isotropic 1 mm^3 resolution and no interslice gap.

During the longitudinal follow-up (ILAS-3, 2018–2024), the scanner was upgraded from a Magnetom Tim Trio to a Magnetom $Prisma^{Fit}$ in 2021. Among 444 participants with repeat MRI, 253 were scanned on the original Trio system using the identical baseline protocol, while 191 were scanned on the upgraded Prisma Fit system using the same 3D MPRAGE sequence with modified repetition time (TR = 2500 ms; all other parameters unchanged). Comparison of brain metrics between these two groups confirmed systematic differences attributable to scanner upgrade (Table S11), necessitating appropriate correction in longitudinal analyses.

All anatomical scans underwent visual quality inspection by an experienced neuroradiologist to exclude participants with incidental findings (such as brain tumors, traumatic lesions, or large territorial infarcts) or inadequate image quality resulting from excessive head motion or acquisition artifacts. Participants with poor-quality scans were excluded from the analysis, as described in the Study Population section.

Clinical biomarkers and physical function assessments

Following a minimum 10-h overnight fast, peripheral blood samples were collected from all participants between 7:00 and 9:00 a.m. Samples were processed and analyzed using standard laboratory protocols for a comprehensive panel of 46 biomarkers spanning seven physiological domains: metabolic regulation (lipid profile, glucose homeostasis), endocrine function (sex hormones, growth factors), inflammation and immune status (acute-phase proteins, white blood cell differential), hepatic function (transaminases, synthetic markers), renal function (nitrogenous waste products, electrolytes), nutritional status (vitamins, micronutrients), and hematologic parameters (complete blood count indices). The complete list of biomarkers is provided in Table S5.

Physical performance was evaluated through three standardized assessments. Handgrip strength was measured using a digital dynamometer (Smedlay's Dynamo Meter; TTM, Tokyo, Japan) with participants in an upright position using their dominant hand. Gait speed was assessed over a 6-meter walk at the participant's usual pace. Relative appendicular skeletal muscle mass (RASM) was derived from whole-body dual-energy X-ray absorptiometry scans, calculated as the sum of fat-free lean mass from all four limbs divided by height squared (kg/m^2).

Proteomic analysis

Baseline plasma samples from 606 participants with analyzable plasma proteomic data were analyzed. Comparison of baseline characteristics between participants with and without proteomic data is provided in Table S12. Fasting peripheral blood was collected in EDTA-coated tubes and centrifuged at 3,000 g for 10 min at 4°C. Plasma was separated and stored at −80°C until analysis. Protein digestion was performed using the SMART Digest Trypsin kit (Thermo Fisher Scientific). Each 10 μL plasma sample was diluted with digestion buffer and incubated at 70°C for 2 h to achieve complete tryptic digestion. Digested peptides underwent reduction, alkylation with iodoacetamide, and purification through solid-phase extraction using SOLAμ plates. Samples were subsequently desalted with C18 ZipTip (Merck) prior to mass spectrometry analysis.

Peptide separation and analysis were conducted using nano-liquid chromatography coupled to an Orbitrap Elite mass spectrometer (Thermo Fisher Scientific). Peptides were eluted over 210 min using a segmented gradient on a C18 column. Mass spectrometry data were acquired in data-dependent mode with 60-s dynamic exclusion.

Raw spectral data were processed using Proteome Discoverer version 2.4 (Thermo Fisher Scientific) with database searches performed against the human UniProt database using both Mascot and Sequest HT algorithms. Search parameters allowed one missed tryptic cleavage site, with carbamidomethylation of cysteine as a fixed modification and methionine oxidation as a variable modification. Mass tolerance was set at 20 ppm for precursor ions and 0.8 Da for fragment ions. Protein quantification was based on MS1-level intensities, calculated as the sum of unique peptide abundances. Data normalization utilized the total peptide amount across all samples. Proteins detected in fewer than 50% of samples were excluded, resulting in 513 proteins for statistical analysis.

Cognitive assessments

Cognitive function was evaluated through a standardized neuropsychological battery administered by trained research personnel during face-to-face interviews. Global cognitive performance was assessed using the Mini-Mental State Examination (MMSE). Domain-specific cognitive abilities were assessed using five tests that spanned four cognitive domains. Memory function was evaluated through the 10-min delayed recall component of the Chinese Version Verbal Learning Test (CVVLT).[45] Language ability was assessed with the Boston Naming Test (BNT).[46] Visuospatial function was measured using the copy task from the Taylor Complex Figure Test (TCF).[47] Executive function was evaluated through the Backwards Digit Test (BDT).[48] The Clock Drawing Test (CDT) assessed both visuospatial and executive functions.[49,50]

For longitudinal analysis in the subset with repeat assessments (n = 444), cognitive decline was operationally defined as performance deterioration exceeding the 90th percentile (i.e., a top 10% decline) in any of the five domain-specific tests (CVVLT, BNT, TCF, BDT, CDT). Change scores were calculated as the difference between follow-up and baseline performance for each test. Participants who demonstrated decline in one or more tests were classified as having cognitive decline for subsequent survival analysis.

Image processing and feature extraction

Individual T1-weighted images from both the ILAS cohort and an independent training dataset (see "Brain Age Prediction Model" section, n = 1,482) were preprocessed using a voxel-based morphometry pipeline implemented in Statistical Parametric Mapping 12 (SPM12, version 7487; Wellcome Trust Center for Neuroimaging, University College London, UK) within MATLAB R2021a (MathWorks, Natick, MA, USA). The complete neuroimaging processing and analysis workflow is illustrated in Figure S3. The preprocessing workflow employed a high-dimensional Diffeomorphic Anatomical Registration Through Exponentiated Lie Algebra (DARTEL) approach, following protocols established and validated in our previous studies.[51] The pipeline comprised five sequential steps: (1) bias field correction to address intensity inhomogeneities; (2) tissue segmentation to classify gray matter, white matter, and cerebrospinal fluid; (3) construction of a study-specific tissue template from the training dataset, to which both training and ILAS images were spatially normalized and subsequently aligned to Montreal Neurological Institute (MNI) standard space; (4) tissue modulation to preserve absolute tissue volumes following spatial transformation; and (5) spatial smoothing with a 6-mm full-width at half-maximum Gaussian kernel. To account for individual differences in overall brain size, global tissue volume, and total intracranial volume (TIV = gray matter + white matter + cerebrospinal fluid volumes) were estimated for each participant in native space.

Regional gray matter features were extracted using a composite brain atlas comprising 442 regions of interest. Cortical parcellation utilized the Schaefer 400-region functional atlas,[40] which delineates the cerebral cortex into seven large-scale networks (visual, somatomotor, dorsal attention, ventral attention/salience, limbic, central executive, and default mode) based on intrinsic functional connectivity patterns. Subcortical structures were defined using 14 regions from the Harvard-Oxford subcortical atlas,[41,42] and cerebellar parcellation employed 28 regions from the spatially unbiased infratentorial template.[43] Two types of feature sets were extracted from the resultant gray matter volume images. For global brain age modeling, mean gray matter volume was computed for each of the 442 regions by averaging values across all voxels within that region. For regional brain age modeling, voxel-wise gray matter volumes within each region were retained as separate features, enabling region-specific age prediction models. The voxel count of each region is listed in Table S13.

Brain age prediction model

Training dataset

To develop robust brain age prediction models, we compiled T1-weighted anatomical scans from 1,482 healthy participants (age range 18–92 years; 681 males, 801 females) recruited across five research sites in Taiwan utilizing six MRI scanners. Participants were free from neuropsychiatric disorders, head trauma, and major medical conditions. Site-specific scanner specifications and imaging parameters are detailed in Table S14 and Figure S4A.

Model architecture and training

We employed support vector regression with radial basis function kernel (RBF-SVR) to construct global and regional brain age prediction models, following the validated framework established in our previous study.[51] The brain age model was trained on the combined sample of male and female participants, consistent with established conventions in brain age modeling[9,52] and the sample size requirements demonstrated in recent large-scale evaluations[53,54]; sex was not entered as a model input but was incorporated as a covariate in all downstream mortality and biomarker analyses. Model training utilized nested 5-fold cross-validation, wherein the inner loop performed hyperparameter optimization (C and gamma parameters) via GridSearchCV across the search space [0.001, 0.01, 0.1, 1, 10, 100, 1000], while the outer loop evaluated model performance using mean absolute error (MAE) and coefficient of determination (R^2) (Figures S4B and S4C). To assess whether regional model performance was influenced by region size, Spearman

correlations between voxel count and prediction accuracy were examined across the 442 regional models. Although statistically significant, the correlations were weak (MAE: rho = −0.12, $p = 0.010$; R^2: rho = 0.13, $p = 0.006$), indicating that region size explained less than 2% of the variability in regional model performance. Voxel counts and prediction performance metrics for all 442 regions are provided in Table S13. Final models trained on the entire training dataset were then applied to generate brain age predictions for all ILAS participants (n = 1,240 at baseline and n = 444 at longitudinal follow-up).

Age-bias correction and brain age gap calculation

Brain age prediction models inherently exhibit estimation bias due to "regression toward the mean," whereby younger individuals' brain ages tend to be overestimated, and older individuals' brain ages tend to be underestimated. To address this age-dependent bias, we implemented a systematic correction procedure following the established method.[9] Specifically, a linear regression model was fitted to the training dataset to characterize the relationship between predicted brain age and chronological age (predicted age = $\alpha \times$ chronological age + β). The resulting regression parameters (α and β) were then used to derive corrected brain age estimates for all ILAS participants via the formula: corrected brain age = (predicted age − β)/α. The individual brain age gap (BAG) was calculated as the difference between the corrected brain age and chronological age.[52] Both global and regional BAG metrics were computed for subsequent analyses.

Longitudinal brain aging phenotypes

A subset of 444 participants underwent repeat brain MRI and cognitive assessments during longitudinal follow-up (ILAS-3). To characterize overall brain aging trajectories at the individual level and ensure comparability with existing longitudinal brain age literature, we used global BAG to define aging phenotypes.[10,38] For each participant, we calculated the change in brain age gap (ΔBAG) as the difference between follow-up and baseline BAG values ($\Delta BAG = BAG_{follow\text{-}up} - BAG_{baseline}$). The annual rate of brain age gap change (BAG slope) was then computed by dividing ΔBAG by the individual inter-scan interval in years.

These participants were classified into three brain aging phenotypes based on the distribution of BAG slopes across the longitudinal cohort. To account for potential confounding effects of baseline BAG and MRI scanner upgrade (191 of 444 participants scanned on upgraded scanner at follow-up), we adjusted the BAG slope using a general linear model with baseline BAG and scanner upgrade status as covariates, and the resulting residuals were *Z* score standardized. Participants were classified into three brain aging phenotypes based on the distribution of adjusted z-scores. Delayed Aging (DA) was defined as Z score < -1 (equivalent to adjusted BAG slope <mean − 1 standard deviation), indicating brain aging slower than expected given baseline status. Normal Aging (NA) was defined as $-1 \le Z$ score $\le +1$, reflecting typical aging trajectories. Accelerated Aging (AA) was defined as Z score $> +1$, indicating brain aging faster than expected given baseline status.

QUANTIFICATION AND STATISTICAL ANALYSIS

Descriptive statistics and group comparisons

Baseline characteristics of the cross-sectional cohort were summarized using descriptive statistics. Continuous variables were presented as mean ± standard deviation, and categorical variables as frequencies and percentages (n, %). Group comparisons were performed to characterize mortality-associated baseline factors and brain age gap distributions. Comparisons between survival status (alive vs. death) assessed baseline predictors of mortality. Demographic and lifestyle variables were compared using Student's *t* test for continuous variables and chi-square test for categorical variables. Variables potentially confounded by demographic factors were compared using analysis of covariance (ANCOVA), with physical function measures adjusted for age and sex; cognitive scores for age, sex, and education; and global brain age gap and brain imaging metrics for age, sex, and TIV. Global brain age gap distributions across cardiovascular risk profiles were evaluated by comparing BAG between participants with and without major risk factors (hypertension, diabetes mellitus, smoking, alcohol consumption) using ANCOVA adjusted for age, sex, and TIV. Statistical significance was defined as two-tailed $p < 0.05$. Analyses were performed using IBM SPSS Statistics version 29 (IBM Corp., Armonk, NY, USA) and MATLAB R2021a (MathWorks, Natick, MA, USA).

Global and regional BAG associations with mortality risk

Cox proportional hazards regression models assessed associations between brain age gaps and all-cause mortality. Time-to-event was defined from baseline MRI to death or censoring (study end, April 2022). Minimally adjusted model included age, sex, and TIV; Fully adjusted model additionally adjusted for cardiovascular and lifestyle risk factors, including education years, hypertension, diabetes, smoking, and alcohol drinking habits. To assess whether the BAG–mortality association was modified by sex, an additional Cox model was fitted incorporating a BAG × sex interaction term in the fully adjusted specification. Proportional hazards assumptions were verified using Schoenfeld residuals tests. Regional mortality associations were identified through univariate Cox regression for each of 442 regional BAGs with Bonferroni correction ($\alpha = 0.05/442 = 1.13 \times 10^{-4}$). Forward stepwise selection based on likelihood ratio tests identified an optimal regional model, starting with base covariates and sequentially adding regional BAGs with significant improvement in model fit. Model performance was evaluated using Harrell's concordance index (C-index, calculated using JASP version 0.95.1; JASP Team, Amsterdam, Netherlands) across models incorporating different combinations of global BAG, optimal regional BAGs, and covariates.

Molecular correlates of brain aging

Associations between brain age gaps (global and three optimal regional BAGs) and molecular biomarkers were evaluated using partial Spearman correlation adjusted for age, sex, and TIV. Two sets of correlation analyses were performed: (1) 55 baseline assessments comprising 46 blood markers, 3 physical function assessments, and 6 cognitive test scores (complete list provided in Table S5) with false discovery rate correction ($q < 0.05$) applied within each brain age measure; and (2) 513 plasma proteins (n = 606) without multiple comparison correction given the exploratory nature. Plasma protein data were log-transformed and Z score standardized prior to statistical analysis. To elucidate biological processes underlying protein-brain age associations, pathway enrichment analysis was performed using the Database for Annotation, Visualization and Integrated Discovery (DAVID; https://david.ncifcrf.gov/), with Gene Ontology biological process as the primary annotation category. To assess robustness across pathway annotation frameworks, cross-database validation was additionally performed using KEGG, Reactome, and WikiPathways.

Longitudinal brain aging phenotypes and cognitive outcomes

Baseline characteristics were compared across the three aging phenotypes (delayed, normal, accelerated) using analysis of variance for continuous variables and chi-square tests for categorical variables, with analysis of covariance adjustment for brain metrics (age, sex, TIV, MRI scanner type). Cox proportional hazards regression assessed associations between aging phenotypes and risk of cognitive decline during follow-up, with the normal aging group as reference. Hazard ratios with 95% confidence intervals were reported. Kaplan-Meier curves were used to visualize cognitive decline-free survival across phenotypes. To identify molecular signatures distinguishing aging phenotypes, plasma protein levels were compared between accelerated and delayed aging groups ($n = 31$ vs. $n = 40$) using analysis of covariance adjusted for baseline age and sex. Statistical significance was defined at $p < 0.05$.

Cell Reports Medicine, Volume *7*

Supplemental information

Regional brain aging signatures predict mortality through neurovascular immune mechanisms

Pei-Lin Lee, Yi-Long Huang, Chih-Kuang Liang, Wei-Ju Lee, Li-Ning Peng, Fei-Yuan Hsiao, Ching-Po Lin, and Liang-Kung Chen

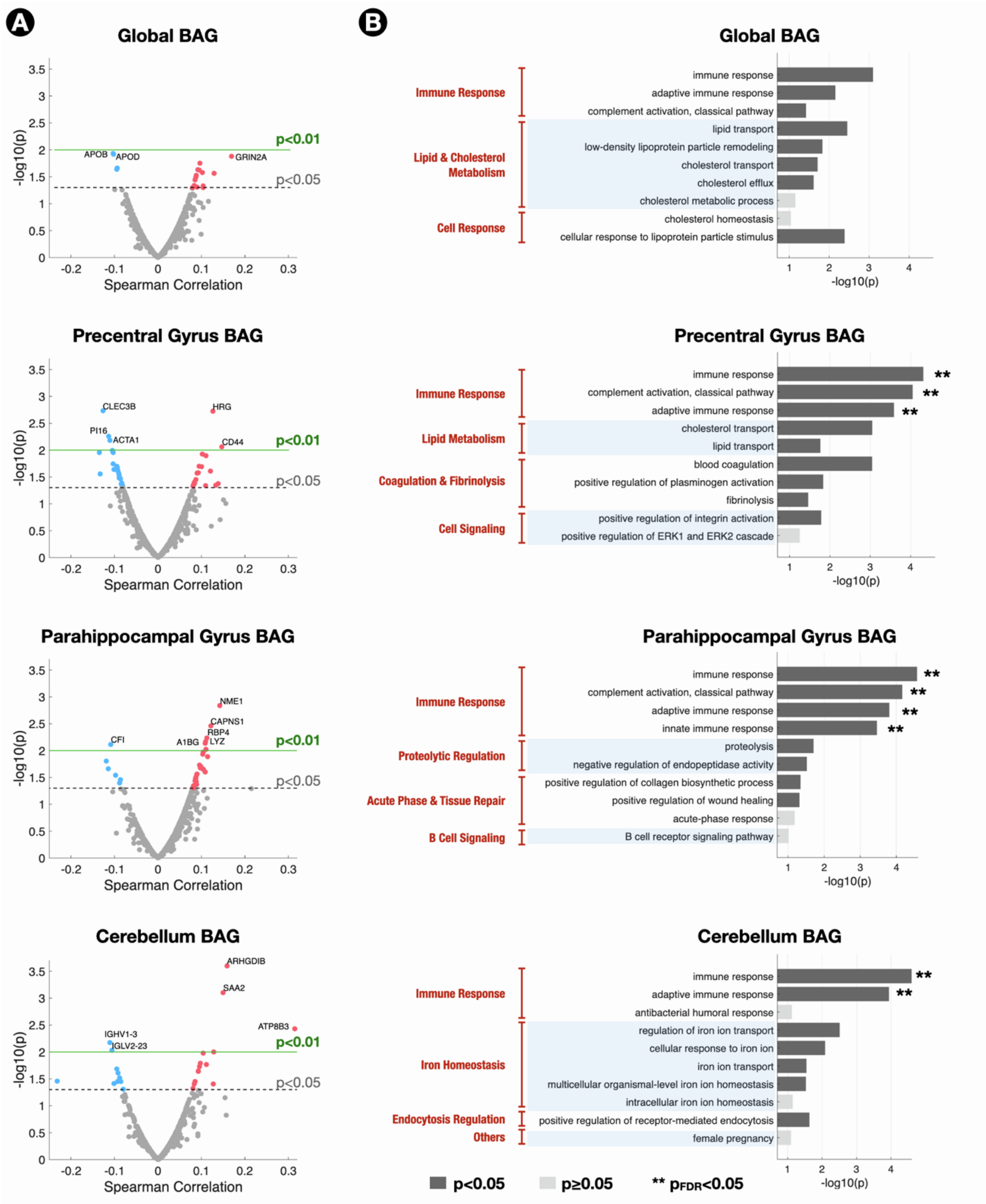

Figure S1. Proteomic Correlates of Global and Regional Brain Age Gaps. Related to Figure 4.

(A) Association between plasma proteins and brain age gaps (n=606 participants). Each panel displays Spearman correlations (x-axis) versus statistical significance (y-axis, $-\log_{10}$ p-value) for approximately 500 quantified proteins. All correlations adjusted for age, sex, and TIV. Blue points indicate negative correlations; red points indicate positive correlations; gray points indicate non-significant associations ($p \geq 0.05$). Horizontal dashed lines mark significance thresholds ($p=0.05$, gray; $p=0.01$, green). (B) The representative biological processes of GO enrichment analyses. A summary of the terms is provided to the left (red labels). Dark gray bars indicate $p<0.05$; light gray bars indicate $p \geq 0.05$. ** indicate $p<0.05$ after false discovery rate correction.

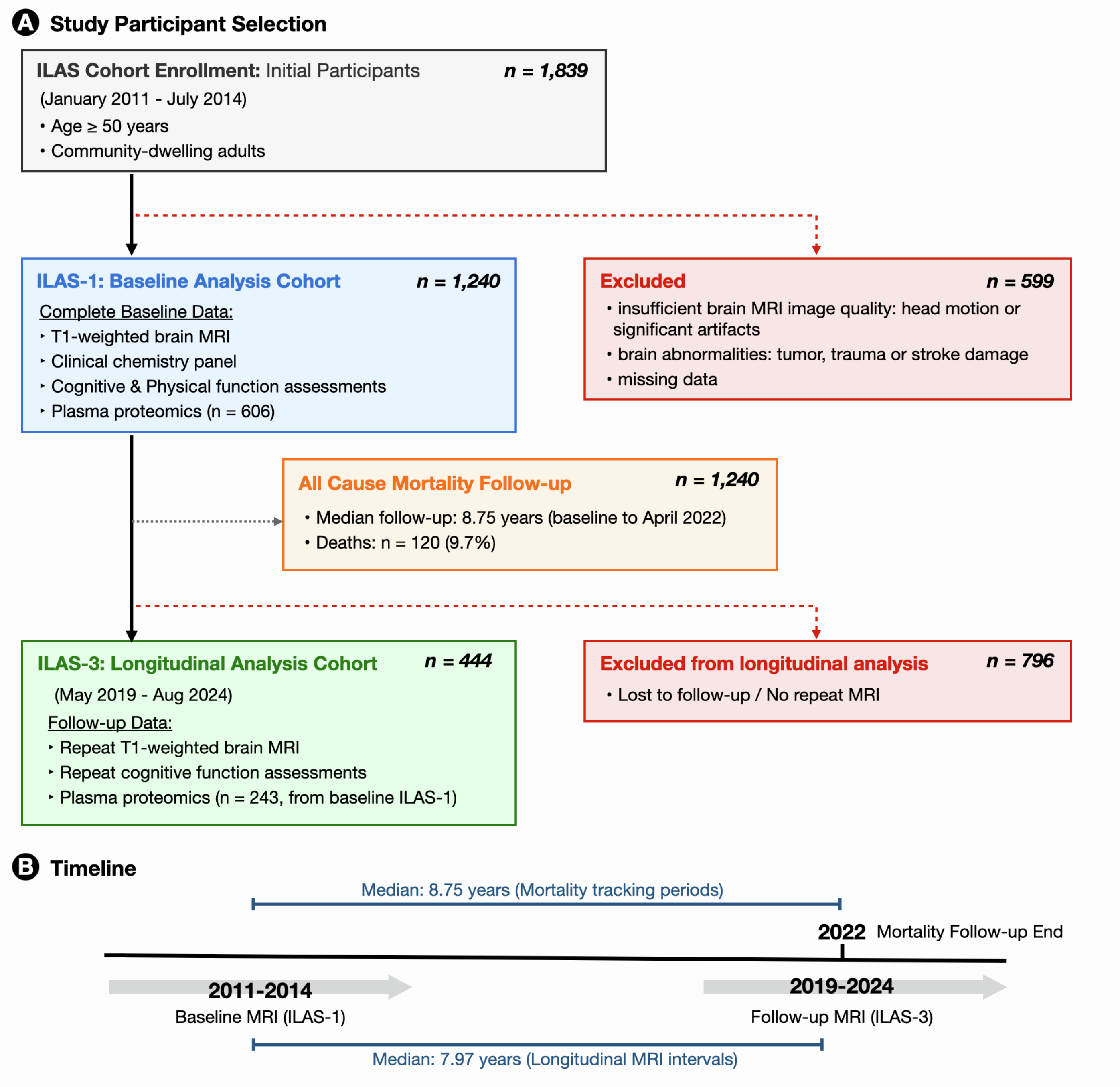

Figure S2. Study population recruitment. Related to STAR Methods.

Flowchart depicting participant selection through ILAS study phases. Phase 1 (2011-2014): Initial enrollment of 1,839 community-dwelling adults. After excluding 599 participants (poor MRI quality, neurological conditions, missing data), the baseline cohort comprised 1,240 participants (ILAS-1) with complete brain MRI and assessments. Phase 2: All 1,240 participants were followed for mortality (median 8.75 years, 120 deaths by April 2022). Phase 3 (2019-2024): Longitudinal cohort of 444 participants (ILAS-3) completed repeat MRI (median 7.97-year interval). Among these, 243 participants had baseline plasma proteomics data available from ILAS-1 for analysis. The timeline shows study milestones and data-collection periods.

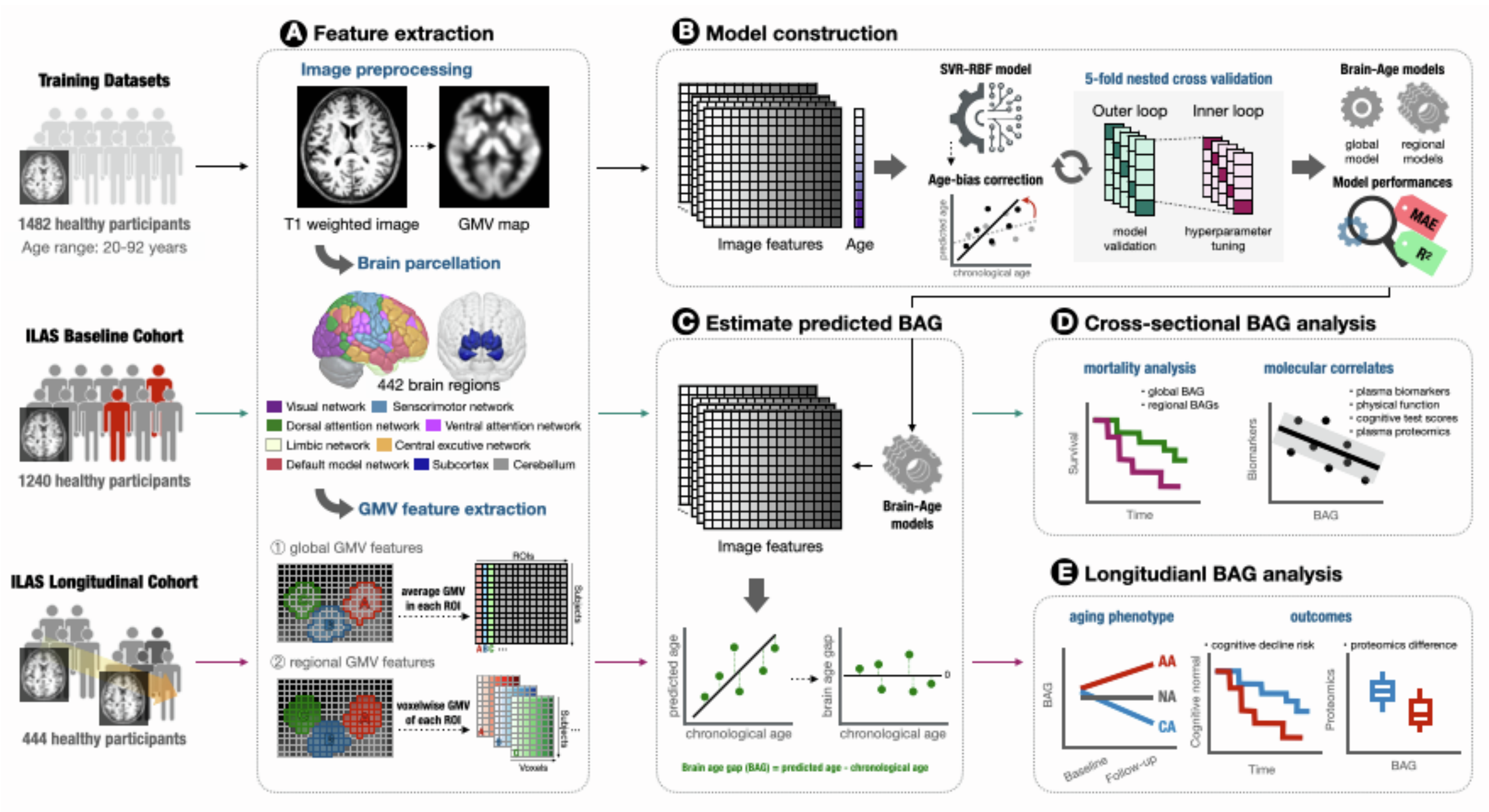

Figure S3. Analysis Framework of the study. Related to STAR Methods.

(A) Structural MRI data were preprocessed using the VBM pipeline in SPM12, and gray matter volume features were extracted globally (averaged across 442 regions) and regionally (voxel-wise within each region). (B) These imaging features were used to construct and validate brain age predictive models using SVR-RBF with nested 5-fold cross-validation in an independent training dataset of 1,482 healthy participants. Age-bias correction was applied, and model performance was evaluated. (C) The established models were applied to ILAS cohorts to estimate individual brain ages, from which brain age gaps were calculated. (D) In the baseline cohort (n=1,240), statistical analyses were conducted to examine associations between global and regional BAG and 10-year mortality risk using Cox regression. Molecular correlates were investigated through associations with clinical biomarkers and plasma proteins. (E) In the longitudinal cohort (n=444), participants were classified into brain aging phenotypes (delayed, normal, accelerated) based on annual rate of BAG change adjusted for baseline BAG and MRI scanner upgrade. Associations between aging phenotypes and cognitive decline risk were examined, and proteomic differences between extreme phenotypes were evaluated.

Abbr. AA, accelerated aging; BAG, brain age gap; DA, delayed aging; GMV, gray matter volume; ILAS, I-Lan Longitudinal Aging Study; MAE, mean absolute error; NA, normal aging; SVR-RBF, Support Vector Regression with Radial Basis Function kernel.

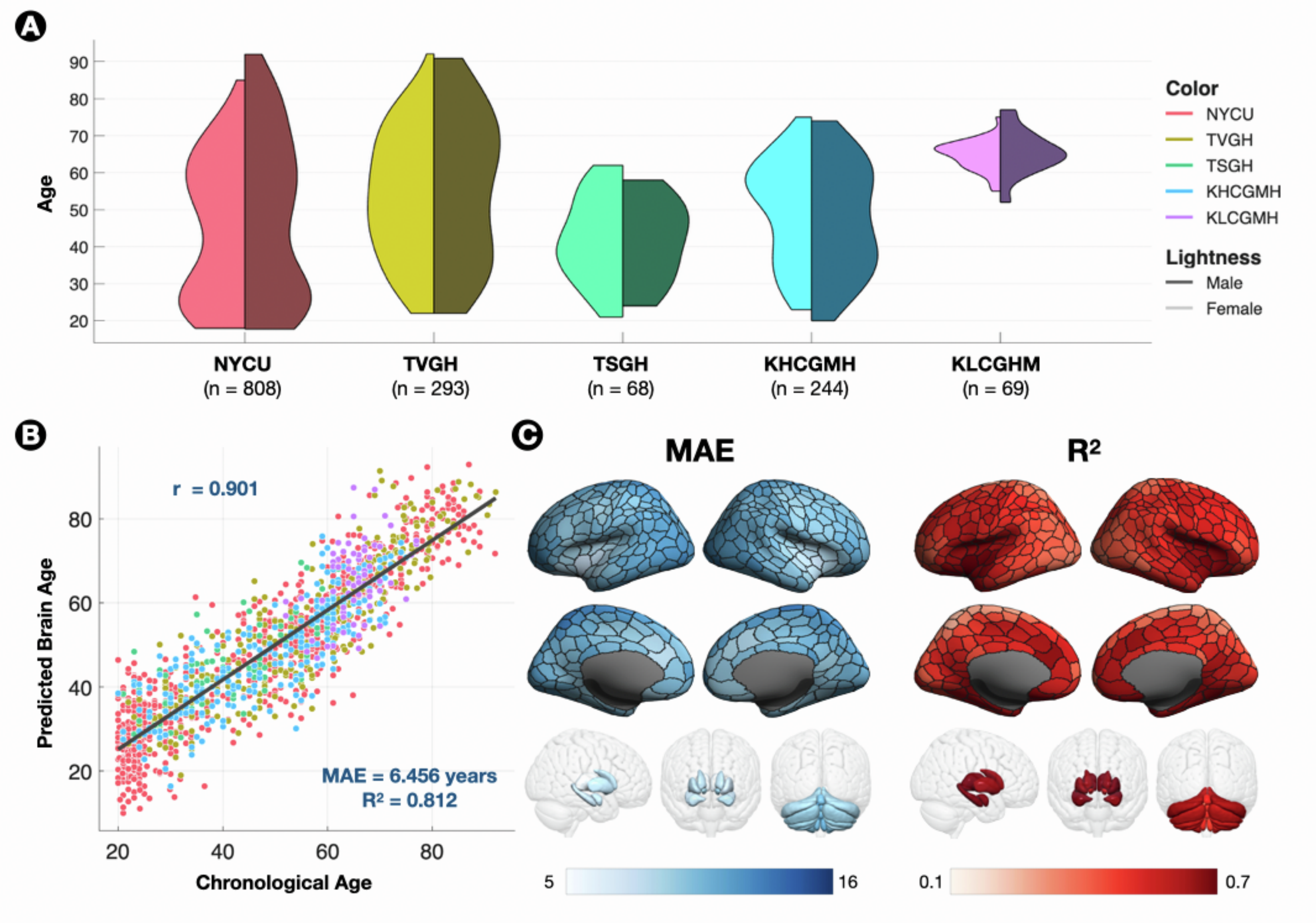

Figure S4. Brain Age Prediction Model Performance. Related to STAR Methods.

Characteristics of the training dataset and performance of the brain age model. (A) Age and sex distribution of the training cohort (n=1,482 healthy participants from 5 research sites). (B) Global brain-predicted age was highly associated with chronological age (r = 0.901, P < 0.001; MAE = 6.46 years and R2 = 0.812). (C) Regional brain-age prediction models also demonstrated moderate to good prediction (MAE range from 5.772 to 13.598 years and R2 range from 0.234 to 0.847).

MAE = mean absolute error; r = correlation coefficient; R2 = coefficient of determination.

Table S1. Baseline characteristics of study samples. Related to Figure 1.

	Cross-sectional samples (n=1240)			p-value
	**Overall**	**Alive**	**Death**	(alive vs. death)
Demographic				
N	1240	1120	120	-
Age, years	63.1 ± 8.8	62.2 ± 8.3	70.9 ± 9.7	<0.001
Female, n (%)	656 (52.9%)	614 (54.8%)	42 (35.0%)	<0.001
Education, years	6.8 ± 5.0	7.1 ± 5.0	4.4 ± 4.9	<0.001
Follow-up time, years	8.66 ± 1.30	8.92 ± 0.82	6.21 ± 2.12	-
Lifestyle and Risk factors				
Smoking , n (%)	210 (16.9%)	171 (15.3%)	39 (32.5%)	<0.001
Alcohol drinking, n (%)	442 (35.6%)	405 (36.2%)	37 (30.8%)	0.271
Hypertension, n (%)	478 (38.5%)	402 (35.9%)	76 (63.3%)	<0.001
Diabetes mellitus, n (%)	188 (15.2%)	157 (14.0%)	31 (25.8%)	<0.001
Physical examination				
Handgrip, kg	29.0 ± 9.5	29.2 ± 9.5	26.8 ± 9.1	<0.001[a]
Gait speed, m/s	1.6 ±0.5	1.6 ± 0.5	1.3 ± 0.5	<0.001[a]
Cognitive performance, scores				
Mini-Mental State Examination	26.2 ± 3.6	26.4 ± 3.5	24.1 ± 4.6	0.041[b]
10-min CVVLT	6.6 ± 2.1	6.7 ± 2.0	5.5 ± 2.5	0.084[b]
Clock drawing test	7.7 ± 2.5	7.8 ± 2.4	6.3 ± 2.9	0.067[b]
Taylor Complex Figure test	30.6 ± 7.1	31.0 ± 6.7	27.0 ± 9.3	0.119[b]
Boston Naming test	12.4 ± 2.5	12.5 ± 2.4	11.3 ± 2.9	0.670[b]
Backward Digit test	3.6 ± 2.0	3.7 ± 1.9	2.7 ± 1.9	0.301[b]
Neuroimaging measurement				
Total intracranial volume, mL	1336 ± 118	1334 ± 119	1358 ± 113	0.471[a]
Gray-matter volume, mL	629 ± 61	631 ± 59	603 ± 69	<0.001[c]
White-matter volume, mL	416 ± 50	417 ± 49	403 ± 53	0.017[c]
Cerebrospinal fluid volume, mL	292 ± 73	285 ± 70	352 ± 66	<0.001[c]
Global brain age gap, years	1.06 ± 9.33	0.63 ± 9.28	5.11 ± 8.89	<0.001[c]

[a]ANCOVA test, adjusted for age and sex.

[b]ANCOVA test, adjusted for age, sex and education years.

[c]ANCOVA test, adjusted for age, sex and total intracranial volume.

Abbreviations: CVVLT = Chinese version Verbal Learning Test

Table S4. Spearman correlations among global BAG and the three optimal regional BAGs (n=1,240). Related to Figure 3 and Table 2.

	Global BAG	Regional BAG in left precentral gyrus	Regional BAG in left parahippocampal gyrus	Regional BAG in right cerebellum I-IV
**Global BAG**	-	0.44	0.43	0.37
**Regional BAG in left precentral gyrus**	0.44	-	0.35	0.29
**Regional BAG in left parahippocampal gyrus**	0.43	0.35	-	0.37
**Regional BAG in right cerebellum I-IV**	0.37	0.29	0.37	-

All correlations $p<0.001$.

Table S5. Association between brain age metrics and multi-domain biomarker. Related to Figure 4.

Biomarker category	Biomarker Abbr.	Biomarker	Global BAG		Regional BAG in precentral gyrus		Regional BAG in parahippocampal gyrus		Regional BAG in cerebellum I-IV	
			rho	p-value	rho	p-value	rho	p-value	rho	p-value
Metabolic Regulation	TC	Total Cholesterol	-0.032	0.268	-0.026	0.362	-0.058	0.040	-0.044	0.119
Metabolic Regulation	TG	Triglycerides	0.024	0.391	0.053	0.065	-0.005	0.860	0.005	0.857
Metabolic Regulation	A1c	Glycated Hemoglobin	0.079	0.005	0.133	0.000	0.076	0.008	0.051	0.072
Metabolic Regulation	HDL	High-Density Lipoprotein	0.005	0.862	-0.007	0.817	-0.009	0.760	0.011	0.701
Metabolic Regulation	LDL	Low-Density Lipoprotein	-0.051	0.071	-0.053	0.064	-0.051	0.074	-0.049	0.083
Metabolic Regulation	Insulin	Insulin	0.024	0.390	0.053	0.064	0.062	0.028	0.035	0.218
Endocrine	GH	Growth Hormone	0.030	0.290	0.003	0.921	0.041	0.150	0.062	0.029
Endocrine	Testo	Testosterone	-0.016	0.579	-0.039	0.175	0.037	0.190	-0.019	0.504
Endocrine	SHBG	Sex Hormone Binding Globulin	0.033	0.252	-0.013	0.648	-0.018	0.536	-0.008	0.786
Endocrine	FT	Free Testosterone	-0.038	0.177	-0.034	0.233	0.006	0.828	-0.047	0.095
Endocrine	FAI	Free Androgen Index	-0.048	0.092	-0.035	0.217	-0.004	0.882	-0.043	0.133
Endocrine	IGF	Insulin-like Growth Factor	-0.088	0.002	-0.061	0.031	-0.033	0.247	-0.042	0.143
Endocrine	DHEA	Dehydroepiandrosterone	-0.015	0.594	-0.043	0.131	0.011	0.691	-0.048	0.088
Endocrine	iPTH	Intact Parathyroid Hormone	0.000	0.998	0.015	0.601	-0.018	0.523	0.022	0.449
Inflammation & Immune	hsCRP	High-sensitivity C-Reactive Protein	-0.009	0.756	0.008	0.800	0.045	0.136	0.051	0.090
Inflammation & Immune	HCY	Homocystein	0.084	0.003	0.059	0.039	0.079	0.006	0.083	0.004
Inflammation & Immune	WBC	White Blood Cell count	0.087	0.002	0.084	0.003	0.077	0.007	0.011	0.687
Inflammation & Immune	NEUT	Neutrophil	0.096	0.001	0.038	0.180	0.102	0.000	0.029	0.303
Inflammation & Immune	LYMPH	Lymphocytes	-0.109	0.000	-0.055	0.053	-0.101	0.000	-0.032	0.256
Inflammation & Immune	Mono	Monocytes	0.032	0.266	0.058	0.043	0.045	0.114	0.044	0.126
Inflammation & Immune	Eo	Eosinophil	-0.027	0.347	-0.018	0.525	-0.077	0.006	-0.035	0.214
Inflammation & Immune	Baso	Basophil	-0.020	0.483	-0.028	0.327	-0.035	0.223	-0.038	0.184
Hepatic	AST	Aspartate Aminotransferase	-0.024	0.404	-0.023	0.413	0.053	0.065	0.031	0.282
Hepatic	ALT	Alanine Aminotransferase	-0.017	0.554	-0.017	0.562	0.074	0.009	0.022	0.431
Hepatic	ALP	Alkaline Phosphatase	-0.021	0.451	-0.043	0.127	0.028	0.323	0.044	0.121
Hepatic	GGT	Gamma-Glutamyl Transferase	0.066	0.021	0.083	0.004	0.133	0.000	0.109	0.000
Hepatic	ALB	Albumin	-0.027	0.345	0.006	0.824	0.004	0.899	-0.001	0.964
Hepatic	GLO	Globulin	0.013	0.652	-0.003	0.917	0.039	0.172	0.020	0.476

Hepatic	TP	Total Protein	-0.006	0.840	0.005	0.862	0.042	0.141	0.025	0.371
Renal	BUN	Blood Urea Nitrogen	-0.042	0.143	0.011	0.693	-0.036	0.205	0.000	0.999
Renal	UA	Uric Acid	0.038	0.176	0.017	0.546	0.065	0.022	0.101	0.000
Renal	Scr	Serum Creatinine	0.012	0.676	0.033	0.247	0.033	0.240	0.068	0.017
Renal	Ucr	Urine Creatinine	-0.016	0.568	0.041	0.146	0.000	0.993	-0.041	0.148
Renal	Na	Sodium	-0.061	0.032	-0.050	0.081	-0.061	0.031	-0.006	0.827
Renal	K	Potassium	-0.019	0.499	0.000	0.992	0.034	0.226	-0.005	0.867
Renal	mALB	Microalbuminuria	0.109	0.000	0.126	0.000	0.115	0.000	0.070	0.015
Nutritional	B12	Vitamin B12	-0.042	0.138	-0.017	0.556	0.024	0.400	-0.017	0.541
Nutritional	FOL	Folic acid	-0.055	0.053	-0.051	0.073	-0.050	0.077	-0.034	0.230
Nutritional	VitD3	Vitamin D3	0.015	0.600	-0.029	0.314	0.032	0.257	0.031	0.268
Hematologic	RBC	Red Blood Cell count	-0.081	0.005	-0.078	0.006	-0.068	0.017	-0.056	0.051
Hematologic	Hb	Hemoglobin	-0.082	0.004	-0.017	0.554	-0.045	0.112	-0.085	0.003
Hematologic	HCT	Hematocrit	-0.087	0.002	-0.016	0.578	-0.049	0.083	-0.075	0.008
Hematologic	MCV	Mean Corpuscular Volume	0.013	0.658	0.091	0.001	0.035	0.220	0.014	0.612
Hematologic	MCH	Mean Corpuscular Hemoglobin	0.006	0.828	0.060	0.035	0.036	0.202	-0.011	0.703
Hematologic	MCHC	Mean Corpuscular Hemoglobin Concentration	0.040	0.158	0.045	0.111	0.090	0.001	0.016	0.580
Hematologic	PLT	Platelet count	0.048	0.089	0.065	0.022	0.057	0.045	0.018	0.536
Physical function	Grip	Handgrip strength	-0.130	0.000	-0.130	0.129	-0.090	0.002	-0.105	0.000
Physical function	Speed	Gait speed	-0.056	0.051	-0.056	0.347	-0.139	0.000	-0.028	0.332
Physical function	RASM	Relative Appendicular Skeletal Muscle mass	-0.026	0.365	-0.026	0.634	0.023	0.416	0.039	0.172
Cognitive function	MMSE	Mini-Mental State Examination	-0.076	0.008	-0.076	0.115	-0.105	0.000	-0.076	0.007
Cognitive function	CVVLT	Chinese version Verbal Learning Test	-0.036	0.210	-0.036	0.781	-0.065	0.022	-0.040	0.163
Cognitive function	CDT	Clock drawing test	-0.104	0.000	-0.104	0.013	-0.088	0.002	-0.043	0.128
Cognitive function	TCF	Taylor Complex Figure test	-0.130	0.000	-0.130	0.001	-0.112	0.000	-0.106	0.000
Cognitive function	BNT	Boston Naming test	-0.131	0.000	-0.131	0.001	-0.085	0.003	-0.081	0.004
Cognitive function	BDT	Backward Digit test	-0.089	0.002	-0.089	0.028	-0.098	0.001	-0.066	0.020

Partial spearman correlation analysis adjusted age, sex and TIV

Table S6. List of proteins significantly associated with brain age metrics. Related to Figure 4.

Protein	rho	p-value	Protein description
Global BAG			
IGKV2D-30	0.102	0.026	immunoglobulin kappa variable 2D-30
AQR	0.130	0.027	aquarius intron-binding spliceosomal factor
IGLV2-23	-0.094	0.022	immunoglobulin lambda variable 2-23
IGLV3-19	0.096	0.018	immunoglobulin lambda variable 3-19
IGHG2	0.087	0.032	immunoglobulin heavy constant gamma 2 (G2m marker)
APOA2	0.089	0.030	apolipoprotein A2
APOB	-0.103	0.012	apolipoprotein B
IGF1	-0.095	0.023	insulin like growth factor 1
APOD	-0.103	0.012	apolipoprotein D
C8G	-0.102	0.012	complement C8 gamma chain
PLEK	0.083	0.046	pleckstrin
IGLL1	0.080	0.050	immunoglobulin lambda like polypeptide 1
PGD	0.090	0.049	phosphogluconate dehydrogenase
CRISP3	0.095	0.024	cysteine rich secretory protein 3
GRIN2A	0.169	0.013	glutamate ionotropic receptor NMDA type subunit 2A
Q8NEJ1	0.092	0.023	uncharacterized protein
PGLYRP2	0.086	0.036	peptidoglycan recognition protein 2
CYP8B1	0.104	0.047	cytochrome P450 family 8 subfamily B member 1
FCGBP	0.104	0.050	Fc fragment of IgG binding protein
Regional BAG in precentral gyrus			
IGLV10-54	0.121	0.025	immunoglobulin lambda variable 10-54
IGHV2-70D	-0.085	0.043	immunoglobulin heavy variable 2-70D
IGKV2-24	0.110	0.046	immunoglobulin kappa variable 2-24
F12	0.084	0.040	coagulation factor XII
IGKV1-17	0.082	0.045	immunoglobulin kappa variable 1-17
IGLV1-44	-0.089	0.030	immunoglobulin lambda variable 1-44
IGLV3-19	0.081	0.047	immunoglobulin lambda variable 3-19
APOA2	0.102	0.012	apolipoprotein A2
ALB	-0.082	0.044	albumin
APOB	-0.090	0.027	apolipoprotein B
LCAT	-0.081	0.046	lecithin-cholesterol acyltransferase
HRG	0.126	0.002	histidine rich glycoprotein
IGLV7-43	-0.101	0.023	immunoglobulin lambda variable 7-43
IGF1	-0.089	0.033	insulin like growth factor 1
APOD	-0.083	0.041	apolipoprotein D
CLEC3B	-0.126	0.002	C-type lectin domain family 3 member B
IGKV2-30	0.111	0.013	immunoglobulin kappa variable 2-30
PLEK	0.092	0.026	pleckstrin
C4A,C4B	-0.086	0.034	Complement C4A, C4B
IGHV3-38-3	-0.093	0.022	immunoglobulin heavy variable 3-38-3
CPN1	-0.094	0.021	carboxypeptidase N subunit 1
IGLL1	0.082	0.044	immunoglobulin lambda like polypeptide 1
CD44	0.147	0.009	CD44 molecule (Indian blood group)
C4BPB	0.100	0.020	complement component 4 binding protein beta

GPX3	-0.094	0.021	glutathione peroxidase 3
IGFBP4	0.133	0.045	insulin like growth factor binding protein 4
BLVRB	-0.092	0.025	biliverdin reductase B
IGFALS	-0.103	0.011	insulin like growth factor binding protein acid labile subunit
KRT20	-0.103	0.018	keratin 20
NPEPPS	-0.135	0.011	aminopeptidase puromycin sensitive
RAP1B	-0.095	0.020	RAP1B, member of RAS oncogene family
ACTA1	-0.111	0.007	actin alpha 1, skeletal muscle
GRIN2A	0.139	0.042	glutamate ionotropic receptor NMDA type subunit 2A
Q5EBM2	0.084	0.041	uncharacterized protein
FRMPD3	-0.133	0.028	FERM and PDZ domain containing 3
Q6DHW4	0.087	0.036	uncharacterized protein
PI16	-0.113	0.005	peptidase inhibitor 16
ZSWIM9	0.095	0.020	zinc finger SWIM-type containing 9
C1RL	0.090	0.027	complement C1r subcomponent like
SERPINA10	-0.105	0.010	serpin family A member 10
<u>Regional BAG in parahippocampal gyrus</u>			
IGLV3-12	0.115	0.013	immunoglobulin lambda variable 3-12
IGLV5-45	0.088	0.038	immunoglobulin lambda variable 5-45
IGHV3-49	0.085	0.036	immunoglobulin heavy variable 3-49
CFB	0.080	0.048	complement factor B
IGLV1-40	0.090	0.028	immunoglobulin lambda variable 1-40
IGHG2	0.090	0.027	immunoglobulin heavy constant gamma 2 (G2m marker)
C1QA	0.089	0.028	complement C1q A chain
RBP4	0.112	0.006	retinol binding protein 4
VTN	0.086	0.034	vitronectin
ANXA1	0.086	0.037	annexin A1
APOB	-0.086	0.035	apolipoprotein B
IGLV7-43	-0.097	0.029	immunoglobulin lambda variable 7-43
A1BG	0.110	0.007	alpha-1-B glycoprotein
GAPDH	0.103	0.012	glyceraldehyde-3-phosphate dehydrogenase
CAPNS1	0.121	0.003	calpain small subunit 1
CFI	-0.109	0.008	complement factor I
VIM	0.081	0.049	vimentin
SERPINF2	0.087	0.032	serpin family F member 2
NME1	0.143	0.001	NME/NM23 nucleoside diphosphate kinase 1
PROZ	-0.088	0.040	protein Z, vitamin K dependent plasma glycoprotein
BLK	-0.114	0.022	BLK proto-oncogene, Src family tyrosine kinase
PGD	0.104	0.022	phosphogluconate dehydrogenase
CRISP3	0.098	0.020	cysteine rich secretory protein 3
LYZ	0.109	0.007	lysozyme
B2M	0.104	0.011	beta-2-microglobulin
RAN	0.105	0.022	RAN, member RAS oncogene family
TPM4	0.081	0.049	tropomyosin 4
CAP1	0.089	0.043	cyclase associated actin cytoskeleton regulatory protein 1
LGALS3BP	0.088	0.032	galectin 3 binding protein
ITIH4	0.096	0.018	inter-alpha-trypsin inhibitor heavy chain 4

PCOLCE	0.109	0.025	procollagen C-endopeptidase enhancer
C6orf132	-0.119	0.016	chromosome 6 open reading frame 132
DKFZp686C11235	0.111	0.010	uncharacterized protein
PRG4	0.082	0.045	proteoglycan 4
PGLYRP2	0.081	0.046	peptidoglycan recognition protein 2
GLIPR2	0.098	0.021	GLI pathogenesis related 2
Regional BAG in cerebellum I-IV			
IGKV1D-8	-0.095	0.021	immunoglobulin kappa variable 1D-8
IGHV1-3	-0.110	0.007	immunoglobulin heavy variable 1-3
ATP8B3	0.315	0.004	ATPase phospholipid transporting 8B3
CST3	0.084	0.040	cystatin C
IGLV2-23	-0.106	0.009	immunoglobulin lambda variable 2-23
IGLV2-8	-0.088	0.030	immunoglobulin lambda variable 2-8
IGLV3-27	-0.086	0.035	immunoglobulin lambda variable 3-27
APOA2	0.086	0.036	apolipoprotein A2
AMBP	0.081	0.048	alpha-1-microglobulin/bikunin precursor
TF	0.098	0.016	transferrin
IGLV7-43	-0.093	0.035	immunoglobulin lambda variable 7-43
CLEC3B	-0.092	0.025	C-type lectin domain family 3 member B
SAA2	0.150	0.001	serum amyloid A2
PZP	0.112	0.017	PZP alpha-2-macroglobulin like
ARHGDIB	0.159	0.000	Rho GDP dissociation inhibitor beta
INHBC	0.128	0.010	inhibin subunit beta C
ANTXR2	-0.102	0.039	ANTXR cell adhesion molecule 2
B2M	0.093	0.023	beta-2-microglobulin
ITIH3	-0.080	0.049	inter-alpha-trypsin inhibitor heavy chain 3
LGALS3BP	0.093	0.023	galectin 3 binding protein
Q569I7	0.104	0.010	uncharacterized protein
SBSN	-0.232	0.035	suprabasin
PUM2	0.127	0.040	pumilio RNA binding family member 2
SH3BGRL3	0.096	0.019	SH3 domain binding glutamate rich protein like 3

Partial spearman correlation analysis adjusted age, sex and TIV

Table S8. List of proteins with significant difference between accelerated aging and delayed aging groups. Related to Figure 5.

Protein	t-value	p-value	Protein description
Delay aging group > Accelerated aging group			
IGLV4_60	3.063	0.003	immunoglobulin lambda variable 4-60
IGHV4_4	2.262	0.027	immunoglobulin heavy variable 4-4
IGLC7	2.137	0.037	immunoglobulin lambda constant 7
IGKV2_29	2.031	0.046	immunoglobulin kappa variable 2-29
SDPR	2.802	0.011	serum deprivation-response protein
HP	2.312	0.024	haptoglobin
IGKV3_20	2.141	0.036	immunoglobulin kappa variable 3-20
IGLV3_19	2.394	0.019	immunoglobulin lambda variable 3-19
SAA2	2.118	0.038	serum amyloid A2
PTGFR	2.029	0.047	prostaglandin F receptor
CRISP3	2.106	0.039	cysteine rich secretory protein 3
TPM4	2.022	0.047	tropomyosin 4
SMCO1	2.279	0.027	single-pass membrane protein with coiled-coil domains 1
Q6DHW4	2.021	0.047	uncharacterized protein
Q6PJG0	2.539	0.014	uncharacterized protein
Q7Z2U7	2.513	0.014	uncharacterized protein
ZSWIM9	2.322	0.023	zinc finger SWIM-type containing 9
ABCC2	2.158	0.035	ATP binding cassette subfamily C member 2
TAS2R4	2.052	0.045	taste 2 receptor member 4
Delay aging group < Accelerated aging group			
IGHV1_69D	-3.526	0.001	immunoglobulin heavy variable 1-69D
IGHV1_69_2	-2.077	0.042	immunoglobulin heavy variable 1-69-2
C4BPA	-2.176	0.033	complement component 4 binding protein alpha
VCAM1	-2.318	0.024	vascular cell adhesion molecule 1
KRT20	-3.341	0.001	keratin 20
TSC2	-2.216	0.034	TSC complex subunit 2

ANCOVA model adjusted baseline age & sex

Table S9. Coverage analysis of the MS-based proteomic panel compared with reference plasma proteome. Related to Discussion

Location/Protein type		Current study	HPA reference	FDR
Part A. Subcellular location distribution.				
	Extracellular Space	209 (42.6%)	727 (17.0%)	5.50E-35
	Cytoplasm	108 (22.0%)	2035 (47.5%)	2.00E-28
	Nucleus	27 (5.5%)	585 (13.7%)	3.70E-08
	Plasma Membrane	56 (11.4%)	815 (19.0%)	1.90E-05
	Other	92 (18.7%)	123 (2.9%)	9.00E-37
Part B. Protein type distribution.				
1	enzyme	74 (15.1%)	1104 (25.8%)	4.60E-07
2	translation regulator	0 (0%)	64 (1.5%)	0.01
3	kinase	9 (1.8%)	165 (3.9%)	0.07
4	transcription regulator	8 (1.6%)	144 (3.4%)	0.09
5	transporter	39 (7.9%)	239 (5.6%)	0.09
6	phosphatase	4 (0.8%)	78 (1.8%)	0.26
7	peptidase	35 (7.1%)	250 (5.8%)	0.42
8	transmembrane receptor	16 (3.3%)	187 (4.4%)	0.42
9	ion channel	4 (0.8%)	27 (0.6%)	0.72
10	growth factor	6 (1.2%)	61 (1.4%)	0.99
11	cytokine	5 (1.0%)	46 (1.1%)	1
12	G-protein coupled receptor	2 (0.4%)	20 (0.5%)	1
13	other	289 (58.9%)	1900 (44.3%)	1.40E-08

Current study includes 492 proteins mapped to IPA categories.

HPA reference includes 4,285 plasma proteins detected by MS from the Human Protein Atlas.

FDR values were computed using Fisher's exact tests followed by Benjamini–Hochberg correction.

Table S10. Comparison of baseline characteristics between return and non-return participants. Related to STAR Methods.

	**Return subset** [Participants in longitudinal analysis] **(n=444)**	**Non-return subset** [Attrition participants] **(n=796)**	**p-value**	**Overall (n=1,240)**
Demographic				
Age, years	59.2 ± 6.3	65.2 ± 9.3	<0.001	63.1 ± 8.8
Female, n (%)	238 (53.6%)	418 (52.5%)	0.712	656 (52.9%)
Education, years	9.1 ± 4.5	5.6 ± 4.8	<0.001	6.8 ± 5.0
Lifestyle and Risk factors				
Smoking, n (%)	53 (11.9%)	157 (19.7%)	<0.001	210 (16.9%)
Alcohol drinking, n (%)	170 (38.3%)	272 (34.2%)	0.147	442 (35.6%)
Hypertension, n (%)	134 (30.2%)	344 (43.2%)	<0.001	478 (38.5%)
Diabetes mellitus, n (%)	46 (10.4%)	142 (17.8%)	<0.001	188 (15.2%)
Neuroimaging measurement				
Total intracranial volume, mL	1339 ± 120	1334 ± 117	0.791[a]	1336 ± 118
Global brain age gap, years	-0.60 ± 9.34	1.99 ± 9.20	0.004[b]	1.06 ± 9.33
Physical examination				
Handgrip, kg	30.7 ± 9.9	28.0 ± 9.2	0.349[a]	29.0 ± 9.5
Gait speed, m/s	1.7 ± 0.5	1.5 ± 0.4	<0.001[a]	1.6 ±0.5
Cognitive performance, scores				
Mini-Mental State Examination	27.5 ± 2.5	25.5 ± 4.0	0.393[c]	26.2 ± 3.6

[a]ANCOVA test, adjusted for age and sex.

[b]ANCOVA test, adjusted for age, sex and total intracranial volume.

[c]ANCOVA test, adjusted for age, sex and education years.

Table S11. Sensitivity analysis about the effect of MRI upgrading in BAG change. Related to STAR Methods.

	**No upgrade** TrioTim	**Upgrade** TrioTim to PrismaFit	**p-value**
N	253	191	
Baseline age, years	59.0 ± 6.2	59.4 ± 6.4	0.486
Female, n (%)	138 (54.5%)	100 (52.4%)	0.701
Baseline TIV, mL	1338 ± 115	1340 ± 126	0.951a
Follow-up TIV, mL	1338 ± 117	1395 ± 139	<0.001b
Baseline BAG, years	-0.60 ± 9.65	-0.60 ± 8.94	0.946c
Follow-up BAG, years	1.29 ± 9.57	3.06 ± 8.57	0.026d
Annual BAG change (ΔBAG), years	0.27 ± 0.54	0.42 ± 0.58	0.007a

[a]ANCOVA test, adjusted for baseline age and sex.

[b]ANCOVA test, adjusted for follow-up age and sex.

[c]ANCOVA test, adjusted for baseline age, sex and baseline total intracranial volume.

[d]ANCOVA test, adjusted for follow-up age, sex and follow-up total intracranial volume.

Table S12. Comparison of baseline characteristics between participants with and without proteomic data. Related to STAR Methods.

	Proteomic subset (n=606)	Non-proteomic subset (n=634)	p-value	Overall (n=1,240)
Demographic				
Age, years	62.7 ± 8.6	63.4 ± 9.0	0.175	63.1 ± 8.8
Female, n (%)	327 (54.0%)	329 (51.9%)	0.466	656 (52.9%)
Education, years	7.2 ± 5.0	6.5 ± 5.0	0.012	6.8 ± 5.0
Lifestyle and Risk factors				
Smoking, n (%)	94 (15.5%)	116 (18.3%)	0.191	210 (16.9%)
Alcohol drinking, n (%)	210 (34.7%)	232 (36.6%)	0.476	442 (35.6%)
Hypertension, n (%)	220 (36.6%)	258 (40.7%)	0.112	478 (38.5%)
Diabetes mellitus, n (%)	93 (15.3%)	95 (15.0%)	0.859	188 (15.2%)
Neuroimaging measurement				
Total intracranial volume, mL	1336 ± 117	1336 ± 119	0.633[a]	1336 ± 118
Global brain age gap, years	0.63 ± 9.23	1.48 ± 9.42	0.174[b]	1.06 ± 9.33
Physical examination				
Handgrip, kg	29.2 ± 9.4	28.8 ± 9.6	0.141[a]	29.0 ± 9.5
Gait speed, m/s	1.7 ± 0.5	1.5 ± 0.4	<0.001[a]	1.6 ±0.5
Cognitive performance, scores				
Mini-Mental State Examination	26.4 ± 3.2	26.0 ± 4.0	0.468[c]	26.2 ± 3.6

[a]ANCOVA test, adjusted for age and sex.

[b]ANCOVA test, adjusted for age, sex and total intracranial volume.

[c]ANCOVA test, adjusted for age, sex and education years.

Table S14. Data sources for brain age training cohort. Related to STAR Methods.

Cohort	N	Age mean±SD [range]	Sex male/female	Scanner	Coil	Sequence	Protocol parameters
National Yang Ming Chiao Tung University (NYCU)	808	46.2±20.4 [18-92]	407/401	Siemens Tim Trio (3T)	12-channel / 32-channel phased-array head coil	MPRAGE	TR/ TE/ TI = 3500/3.5/ 1100 msec; flip angle = 7°; NEX = 1; FOV = 256 x 256 mm^2; matrix size = 256 x 256; 192 sagittal slices; and voxel size = 1.0 x 1.0 x 1.0 mm^3
Taipei Veteran General Hospital (TVGH)	293	54.4±17.8 [22-92]	94/199	GE Discovery MR750 (3T)	8-channel phased-array head coil	IR-FSPGR	TR/TE/TI = 9.4/4.0/450 msec; flip angle = 12°; NEX = 1; FOV = 256 x 256 mm^2; matrix size = 256 x 256; 172 axial slices; and voxel size = 1.0 x 1.0 x 1.0 mm^3
						BRAVO	TR/TE/TI = 9.2/3.7/450 msec; flip angle = 12°; NEX = 1; FOV = 256 x 256 mm^2; matrix size = 256 x 256; 168 axial slices; and voxel size = 1.0 x 1.0 x 1.0 mm^3
Tri-Service General Hospital (TSGH)	68	42.0±11.1 [21-62]	21/47	GE Discovery MR750 (3T)	8-channel phased-array head coil	BRAVO	TR/TE/TI = 10.17/4.16/450 msec; flip angle = 12°; NEX = 1; FOV = 256 × 256 mm^2; matrix size = 256 × 256; 172 axial slices; and voxel size = 1.0 x 1.0 x 1.0 mm^3
Kaohsiung Chang Gung Memorial Hospital (KHCGMH)	244	48.5±14.3 [20-75]	127/117	Siemens Skyra (3T)	20-channel phased-array head/neck coil	MPRAGE	TR/ TE/TI = 1800/2.7/900 msec; flip angle = 9°; NEX = 1; FOV = 230 x 230 mm^2; matrix size = 192 x 192; 144 sagittal slices; and voxel size = 1.2 x 1.2 x 1.2 mm^3
				GE Signa Excite (3T)	8-channel phased-array head coil	IR-FSPGR	TR/ TE/ TI = 9.5/3.9/450 msec; flip angle = 15°; NEX = 1; FOV = 240 × 240 mm^2; matrix size = 512 × 512; 110 axial slices; and voxel size = 0.47 × 0.47 × 1.3 mm^3
Keelung Chang Gung Memorial Hospital (KLCGMH)	69	65.0±5.1 [52-77]	32/37	Siemens Skyra (3T)	20-channel phased-array head/neck coil	MPRAGE	TR/TE/TI = 2,200/2.45/900 msec; flip angle = 8°, NEX=1; FOV = 256 x 256 mm^2; matrix size = 256 x 256; 176 sagittal slices; and voxel size = 1.0 x 1.0 x 1.0 mm^3

Abbreviations: TR, Repetition time; TE, echo time; TI, inversion time; NEX, number of excitations; FOV, field of view;
